# Frailty as an Effect Modifier in Randomized Controlled Trials: A Systematic Review

**DOI:** 10.1007/s11606-024-08732-8

**Published:** 2024-04-09

**Authors:** Aaron Yao, Linhui Gao, Jiajun Zhang, Joyce M. Cheng, Dae Hyun Kim

**Affiliations:** 1 VillageMD Research Institute, Chicago, IL USA; 2 Virginia Commonwealth University, Richmond, VA USA; 3 FastHSR, Glen Allen, VA USA; 4 Qingdao Municipal Hospital, Qingdao, Shandong China; 5 The Johns Hopkins University School of Medicine, Baltimore, MD USA; 6 Hinda and Arthur Marcus Institute for Aging Research, Hebrew SeniorLife, Boston, MA USA; 7 Division of Gerontology, Department of Medicine, Beth Israel Deaconess Medical Center, Harvard Medical School, Boston, MA USA

**Keywords:** frailty, randomized controlled trials, effect modifier

## Abstract

**Background:**

The effect of clinical interventions may vary by patients’ frailty status. Understanding treatment effect heterogeneity by frailty could lead to frailty-guided treatment strategies and reduce overtreatment and undertreatment. This systematic review aimed to examine the effect modification by frailty in randomized controlled trials (RCTs) that evaluate pharmacological, non-pharmacological, and multicomponent interventions.

**Methods:**

We searched PubMed, Web of Science, EMBASE, and ClinicalTrial.gov, from their inception to 8 December 2023. Two reviewers independently extracted trial data and examined the study quality with senior authors.

**Results:**

Sixty-one RCTs that evaluated the interaction between frailty and treatment effects in older adults were included. Frailty was evaluated using different tools such as the deficit accumulation frailty index, frailty phenotype, and other methods. The effect of several pharmacological interventions (e.g., edoxaban, sacubitril/valsartan, prasugrel, and chemotherapy) varied according to the degree of frailty, whereas other treatments (e.g., antihypertensives, vaccinations, osteoporosis medications, and androgen medications) demonstrated consistent benefits across different frailty levels. Some non-pharmacological interventions had greater benefits in patients with higher (e.g., chair yoga, functional walking, physical rehabilitation, and higher dose exercise program) or lower (e.g., intensive lifestyle intervention, psychosocial intervention) levels of frailty, while others (e.g., resistance-type exercise training, moderate-intensive physical activity, walking and nutrition or walking) produced similar intervention effects. Specific combined interventions (e.g., hospital-based disease management programs) demonstrated inconsistent effects across different frailty levels.

**Discussion:**

The efficacy of clinical interventions often varied by frailty levels, suggesting that frailty is an important factor to consider in recommending clinical interventions in older adults.

**Registration:**

PROSPERO registration number CRD42021283051.

**Supplementary Information:**

The online version contains supplementary material available at 10.1007/s11606-024-08732-8.

## BACKGROUND

Frailty is a clinical state of reduced physiologic reserve and increased vulnerability to poor health outcomes. The prevalence of frailty is 12 to 24% in community-dwelling older adults^1^ and almost 50% in hospitalized patients^2^ and nursing home residents.^3^ In the United States, 15% of the older population was frail and 45% was pre-frail.^4^ Those with frailty are predisposed to adverse health events, including falls, disability, dementia, hospitalization, institutionalization in long-term care, and death.^5, 6^ Compared with a robust group, pre-frail and frail older adults incurred more healthcare costs.^7^

Clinicians increasingly consider frailty in treatment decision-making due to its association with poor treatment outcomes.^1^ Nonetheless, our understanding on how the benefits and risks of clinical interventions vary by patients’ frailty remains limited. A more nuanced understanding of treatment effect heterogeneity by frailty could lead to frailty-guided treatment strategies and reduce overtreatment and undertreatment, which could lead to improved health outcomes, better quality of life, and more targeted use of healthcare resources. For instance, a robust patient might tolerate a more aggressive treatment regimen potentially leading to improved disease control or even cure. In contrast, a frail patient with depleted physiologic reserve might benefit more from a less invasive, more supportive approach focused on symptom management. To answer this question, frailty subgroup analyses of randomized controlled trials (RCTs) are increasingly conducted to investigate treatment effect heterogeneity by frailty.^8^

This systematic review was conducted to synthesize the findings from RCTs that assessed treatment effects stratified by participants’ frailty levels. We examined how frailty was assessed in RCTs and whether the efficacy and safety of interventions varied by frailty category.

## METHODS

We followed the PRISMA (Preferred Reporting Items for Systematic reviews and Meta-Analyses) statement to conduct a systematic review.^9^ The protocol was registered in the PROSPERO international prospective register of systematic reviews (registration number CRD42021283051, https://www.crd.york.ac.uk/PROSPERO/display_record.php?RecordID=283051) on 2 November 2021.

Our review addressed a key question: Did frailty modify intervention effects in RCTs of pharmacological, non-pharmacological, and multicomponent interventions?

### Data Sources and Searches

A literature search of PubMed, Web of Science, and EMBASE was performed. ClinicalTrial.gov was also searched to identify related trials. Our search included articles published up until December 8, 2023, and we restricted our search to articles published in English. Two authors (JZ, LG) independently reviewed titles, abstracts, and full-text articles to identify eligible studies. Any discrepancies were discussed and resolved with another author (AY). Moreover, we reviewed reference lists of relevant articles and studies potentially meeting our inclusion criteria to minimize retrieval bias. In certain instances, authors indicated in their papers that frailty modifies intervention effects but did not present these findings in tables, figures, or supplemental files. We made attempts to contact all such authors to collect the relevant data.

### Study Selection

We included studies that involved older adults; encompassed RCTs that utilized pharmacological, non-pharmacological, or multicomponent interventions; were published in English; and stratified participants by frailty or conducted an interaction effect or subgroup analysis of frailty and the intervention. We excluded protocols, reviews, editorials, narrative reviews, case reports, case series, animal studies, duplicate publications, and articles without an available full text. Furthermore, studies that did not measure frailty or use it as a stratification variable were also excluded.

### Data Extraction and Quality Assessment

We extracted the following variables from each study: first author’s name, year of publication, country of study, patient characteristics (number and disease types), intervention type (pharmacological, non-pharmacological, and multicomponent), control measures, frailty measurements, frailty sub-groups, endpoints, and the modifying effects of frailty. The extracted data were organized into three tables based on the frailty measurement tools used (i.e., deficit accumulation frailty index [range, 0 to 1; higher scores indicate more severe frailty], frailty phenotype [robust, pre-frail, and frail categories], and other frailty assessments). Two authors (JZ, LG) conducted this abstraction by reviewing each article. Any disagreements were resolved through discussion with senior authors (AY, DHK).

Cochrane Risk of Bias Tool for randomized trials was used to assess the included studies.^10^ The following domains were included: sequence generation, allocation concealment, blinding of participants and personnel, blinding of outcome assessors, incomplete outcome data, selective outcome reporting, and other sources of bias. Two authors (LG, JZ) independently evaluated each domain as having a high, low, or unclear risk of bias. Conflicts were resolved by discussion with another author (AY).

### Data Synthesis and Analysis

We did not perform meta-analysis due to heterogeneity of frailty tools and intervention types. Instead, we qualitatively synthesized the findings from studies based on the frailty tools used: trials that used (1) deficit accumulation frailty index, (2) frailty phenotype, or (3) other frailty measurement tools. Studies within each category were further grouped into intervention types, which included pharmacological, non-pharmacological, or multicomponent intervention. Two authors (JZ, LG) independently and in duplicate rated the certainty of evidence and resolved disagreements by discussion and consultation with senior authors (AY, DHK).

### Role of the Funding Source

This study was supported by the National Institute on Aging of the National Institutes of Health. The funding source had no role in the design, collection, analysis, or interpretation of the data, or the decision to submit the manuscript for publication.

## RESULTS

### Study Selection and Characteristics of the Included Trials

**Figure 1:**
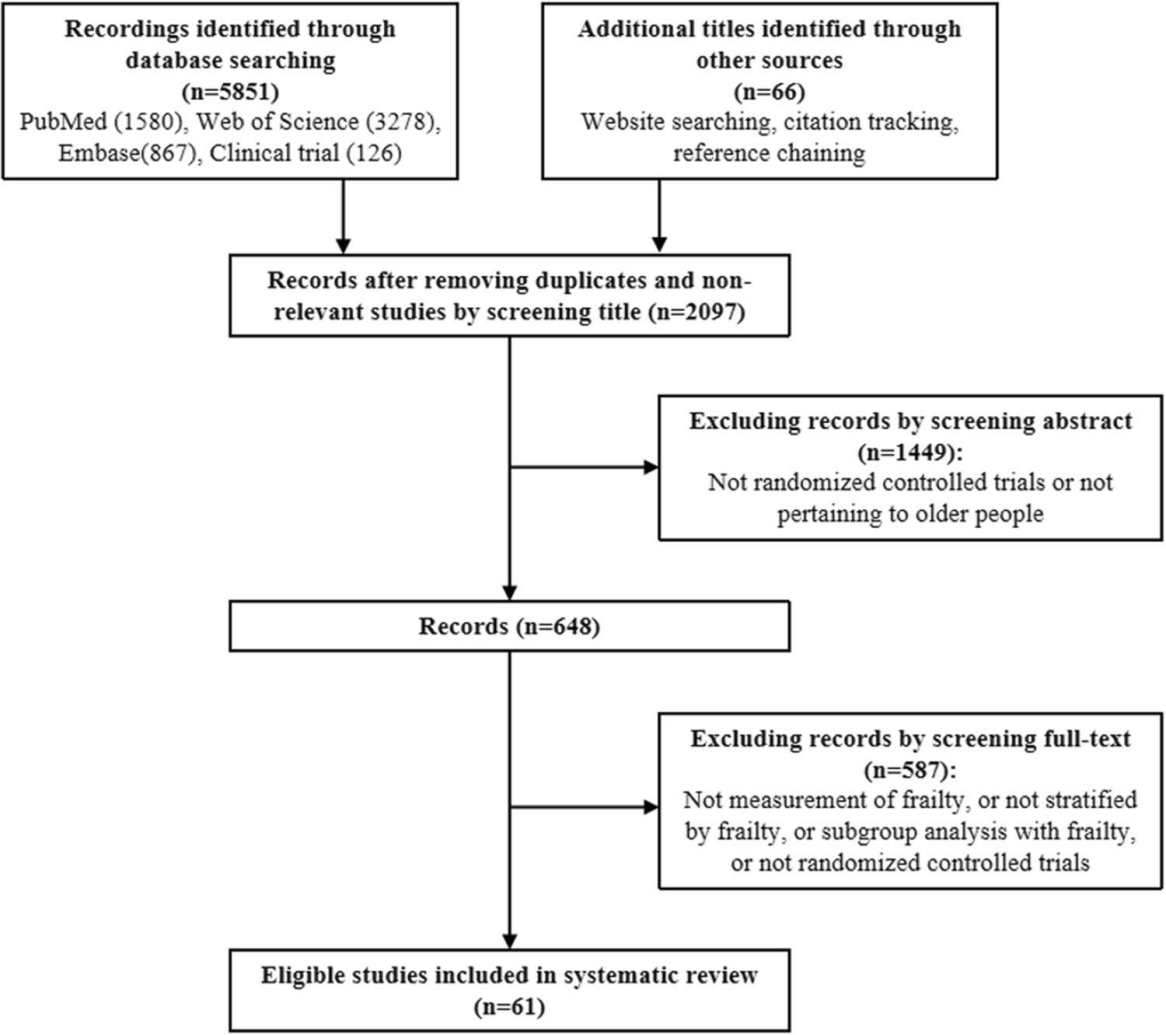
Evidence search and selection.

Figure 1 details study selection. Our database searches yielded 5917 references, of which 61 articles^11–71^ met the inclusion criteria. The included trials are summarized by the type of frailty assessment: frailty index (24 trials) (Table 1), frailty phenotype (17 trials) (Table 2), and other assessments (20 trials) (Table 3). Further details of each trial can be found in Appendix Table. The mean age of trial populations ranged from 58.7^11^ to 87.1^12^ years and the proportion of women ranged from 23.4^29^ to 93.3%^14^ (in particular, one RCT was 100% male^15^ and the other 100% female^16^). There were 26 trials evaluating pharmacological interventions (sample size 40^17^ to 31,989^18^), 27 trials on non-pharmacological interventions (sample size 30^14, 18^ to 5145),^11^ and 8 trials on multicomponent interventions (sample size 173^19^ to 1464).^20^

**Table 1:** Results from 24 Randomized Controlled Trials using Deficit Accumulation Frailty Index

Intervention, population, reference	Frailty assessment	Frailty-specific results	Interpretation
Pharmacological intervention — anticoagulants			
**Intervention:** edoxaban 60 mg vs edoxaban 30 mg vs Warfarin **Population:** 20,867 adults with atrial fibrillation (86.8% were 60 years or older) **Follow-up:** 34 months **Reference:** Wilkinson, 2020, 46 countries^21^	40-item frailty index (range, 0–1) A: frailty index (0 to < 0.12) B: frailty index (0.12 to < 0.24) C: frailty index (0.24 to < 0.36) D: frailty index (0.36 to 1.0)	**Stroke or systemic embolism** (Edoxaban 60 mg vs warfarin) A: HR 1.03 (0.71, 1.49) B: HR 0.82 (0.66, 1.01) C: HR 0.84 (0.61, 1.15) D: HR 0.54 (0.20, 1.50) (Edoxaban 30 mg vs warfarin) A: HR 1.04 (0.71, 1.50) B: HR 1.18 (0.97, 1.43) C: HR 1.17 (0.87, 1.56) D: HR 0.30 (0.08, 1.11) **Major bleeding** (Edoxaban 60 mg vs warfarin) A: HR 0.96 (0.71, 1.30) B: HR 0.76 (0.64, 0.90) C: HR 0.75 (0.57, 0.98) D: HR 0.60 (0.29, 1.26) (Edoxaban 30 mg vs warfarin) A: HR 0.42 (0.28, 0.62) B: HR 0.46 (0.38, 0.56) C: HR 0.47 (0.35, 0.64) D: HR 0.74 (0.36, 1.52)	There was no evidence that the effect of edoxaban compared with warfarin on stroke or systemic embolism was different across the frailty spectrum (*p*-for-interaction = NR) Edoxaban was associated with lower rates of major bleeding compared with warfarin in patients with frailty index 0.12 to < 0.36 (edoxaban 60 mg) and patients with frailty index < 0.36 (edoxaban 30 mg) (*p*-for-interaction = NR)
Pharmacological intervention — antihypertensives			
**Intervention:** antihypertensive medication reduction vs usual care^*^ **Population:** 569 adults with hypertension aged 80 years and older **Follow-up:** 12 weeks **Reference:** Sheppard, 2020, UK^22^	36-item frailty index (range, 0–1) A: frailty index (0 to ≤ 0.12) B: frailty index (0.12 < to 1)	**Systolic blood pressure control** A: RR 0.94 (0.85, ∞) B: RR 1.01 (0.93, ∞) **Change in systolic blood pressure** A: MD 5.14 (1.40, 8.87) B: MD 2.26 (− 0.90, 5.42)	There was no evidence that the effect of antihypertensive medication reduction compared with usual care on systolic blood pressure control (*p*-for-interaction = 0.35) and change in systolic blood pressure (*p*-for-interaction = 0.25) was different across the frailty spectrum
**Intervention:** intensive systolic blood pressure control vs standard control **Population:** 9361 adults aged 50 or older with hypertension; 2636 persons aged 75 years or older with hypertension but without diabetes **Follow-up:** 39 or 38 months **Reference:** Sink, 2018, US^23^; Williamson, 2016, US^25^	36-item frailty index (range, 0–1) A: frailty index (0 to ≤ 0.21) B: frailty index (0.21 < to 1) 37-item frailty index (range, 0–1) A: frailty index (0 to 0.10) B: frailty index (0.10 < to 0.21) C: frailty index (0.21 < to 1)	**Syncope:** NR **Hypotension:** NR **Falls:** NR **Cardiovascular events** A: HR 0.47 (0.13, 1.39) B: HR 0.63 (0.43, 0.91) C: HR 0.68 (0.45, 1.01) **All-cause mortality** A: HR 0.95 (0.27, 3.15) B: HR 0.48 (0.29, 0.78) C: HR 0.64 (0.41, 1.01)	There was no evidence that the effect of intensive treatment compared with standard treatment on syncope (*p*-for-interaction > 0.7), hypotension (*p*-for-interaction > 0.7), falls (*p*-for-interaction > 0.7), cardiovascular events (*p*-for-interaction = 0.84) and all-cause mortality (*p*-for-interaction = 0.52) was different across the frailty spectrum
**Intervention:** indapamide ± perindopril vs placebo **Population:** 3845 adults aged 80 and over with hypertension **Follow-up:** 48 months **Reference:** Warwick, 2015, Europe, China, Australia, Tunisia^24^	60-item frailty index (range, 0–1) A: frailty index 0.1 B: frailty index 0.2 C: frailty index 0.3 D: frailty index 0.4 E: frailty index 0.5 F: frailty index 0.6	**Stroke** A: HR 0.75 (0.40, 1.38) B: HR 0.66 (0.43, 1.01) C: HR 0.59 (0.36, 0.96) D: HR 0.52 (0.25, 1.09) E: HR 0.47 (0.16, 1.33) F: HR 0.41 (0.10, 1.65) **Cardiovascular events** A: HR 0.62 (0.42, 0.92) B: HR 0.60 (0.45, 0.78) C: HR 0.57 (0.42, 0.79) D: HR 0.55 (0.34, 0.89) E: HR 0.53 (0.26, 1.06) F: HR 0.50 (0.20, 1.27) **All-cause mortality** A: HR 0.89 (0.63, 1.25) B: HR 0.84 (0.66, 1.07) C: HR 0.80 (0.61, 1.04) D: HR 0.76 (0.50, 1.14) E: HR 0.72 (0.40, 1.29) F: HR 0.68 (0.32, 1.48)	There was no evidence that the effect of indapamide ± perindopril compared with placebo on stroke (*p*-for-interaction = 0.52), cardiovascular events (*p*-for-interaction = 0.73), and all-cause mortality (*p*-for-interaction = 0.61) was different across the frailty spectrum
**Intervention:** Intensive blood pressure target vs Standard blood pressure target **Population:** 547 adults with hypertension and increased cardiovascular risk but free of diabetes or dementia **Follow-up:** 4 years **Reference:** Dolui, 2022, US^26^	36-item frailty index (range, 0–1) A: frailty index (0 to ≤ 0.10) B: frailty index (0.10 < to ≤ 0.21) C: frailty index (0.21 < to 1)	**Whole Brain Cerebral Blood Flow** A: difference in change 4.50 (0.02, 8.99) B: difference in change 3.18 (0.49, 5.86) C: difference in change − 1.11 (− 5.13, 2.90)	There was no evidence that the effect of intensive treatment compared with standard treatment on whole brain cerebral blood flow (*p*-for-interaction = 0.13) was different across the frailty spectrum
Pharmacological intervention — heart failure medications			
**Intervention:** sacubitril/valsartan vs valsartan **Population:** 4795 patients with HFpEF **Follow-up:** 48 months **Reference:** Butt, 2022(Sep), UK^28^	41-item frailty index (range, 0–1) A: frailty index (0 to 0.210) B: frailty index (0.211 to 0.310) C: frailty index (0.311 to 1)	**HF hospitalizations or cardiovascular death** A: RR 0.98 (0.76, 1.27) B: RR 0.92 (0.76, 1.12) C: RR 0.69 (0.51, 0.95) **HF hospitalizations** A: RR 0.95 (0.71, 1.29) B: RR 0.93 (0.75, 1.16) C: RR 0.64 (0.45, 0.91)	Sacubitril/valsartan was associated with lower rates of heart failure hospitalization or cardiovascular death (*p*-for-interaction = 0.002) and heart failure hospitalization (*p*-for-interaction = 0.001) compared with valsartan in patients with frailty index 0.311 to 1, but not in patients with frailty index < 0.311
**Intervention:** spironolactone 15–45 mg daily vs Placebo **Population:** 1767 adults (mean age = 71.5 years) with HFpEF **Follow-up:** 40 months **Reference:** Sanders, 2018, US, Canada, Brazil, Argentina^27^	39-item frailty index (range, 0–1)^†^ A: frailty index (0 to < 0.3) B: frailty index (0.3 to < 0.4) C: frailty index (0.4 to < 0.5) D: frailty index (0.5 to 1)	**HF hospitalization or cardiovascular death**^†^ A: HR 0.78 (0.52, 1.16) B: HR 0.79 (0.58, 1.06) C: HR 1.00 (0.73, 1.39) D: HR 0.74 (0.51, 1.07)	There was no evidence that the effect of spironolactone compared with placebo on heart failure hospitalization or cardiovascular death was different across the frailty spectrum (*p*-for-interaction = 0.55)
**Intervention:** dapagliflozin vs placebo **Population:** 4742 patients with symptomatic heart failure with a left ventricular ejection fraction of 40% or less and elevated natriuretic peptide **Follow-up:** 18.2 (median) months **Reference:** Butt, 2022(Apr), 20 countries^29^	32-item frailty index (range, 0–1) A: frailty index (0 to 0.210) B: frailty index (0.211 to 0.310) C: frailty index (0.311 to 1)	**18-month worsening HF event or cardiovascular death** A: HR 0.72 (0.59, 0.89) B: HR 0.77 (0.62, 0.97) C: HR 0.71 (0.54, 0.93) **18-month HF hospitalization** A: HR 0.63 (0.48, 0.83) B: HR 0.79 (0.59, 1.07) C: HR 0.68 (0.49, 0.94) **18-month Cardiovascular death** A: HR 0.74 (0.56, 0.98) B: HR 0.83 (0.62, 1.10) C: HR 0.97 (0.67, 1.40) **Refer to **Appendix Table	Dapagliflozin was associated with lower rates of cardiovascular death (*p*-for-interaction = NR) and all-cause death (*p*-for-interaction = NR) compared with placebo in patients with frailty index ≤ 0.210 Dapagliflozin was associated with lower rates of HF hospitalization (*p*-for-interaction = NR) compared with placebo in patients with frailty index ≤ 0.210 or frailty index ≥ 0.311 There was no evidence that the effect of dapagliflozin compared with placebo on worsening HF event or cardiovascular death, HF hospitalization or cardiovascular death, noncardiovascular death, new-onset type 2 diabetes was different across the frailty spectrum (*p*-for-interaction = NR)
**Intervention:** dapagliflozin vs placebo **Population:** 6258 patients with HF and mildly reduced or preserved left ventricular ejection fraction **Follow-up:** 36 months **Reference:** Butt, 2022 (Oct), UK^30^	41-item frailty index (range, 0–1) A: frailty index (0 to 0.210) B: frailty index (0.211 to 0.310) C: frailty index (0.311 to 1)	**HF hospitalizations or cardiovascular death** A: HR 0.85 (0.68, 1.06) B: HR 0.89 (0.74, 1.08) C: HR 0.74 (0.61, 0.91)	There was no evidence that the effect of dapagliflozin compared with placebo on heart failure hospitalization or cardiovascular death was different across the frailty spectrum (*p*-for-interaction = 0.40)
**Intervention:** dapagliflozin (10 mg/day) vs placebo **Population:** 4303 adults with CKD, with/without type 2 diabetes, with an estimated glomerular filtration rate of 25–75 mL/min/1.73 m^2^, and urinary albumin-to-creatinine ratio 200–5000 mg/g **Follow-up:** 2 (median) years **Reference:** Vart, 2023, 21 countries^31^	32-item frailty index (range, 0–1) A: frailty index (0 to 0.210) B: frailty index (0.211 to 0.310) C: frailty index (0.311 to 1)	**eGFR decline ≥ 50%, end-stage kidney disease, or kidney or cardiovascular death** A: HR 0.50 (0.33, 0.76) B: HR 0.62 (0.45, 0.85) C: HR 0.64 (0.49, 0.83) **Kidney composite outcome: eGFR decline ≥ 50%, end-stage kidney disease or kidney death** A: HR 0.42 (0.27, 0.67) B: HR 0.62 (0.44, 0.87) C: HR 0.57 (0.41, 0.9) **Cardiovascular outcome: hospitalization for heart failure or cardiovascular death** A: HR 1.02 (0.43, 2.41) B: HR 0.70 (0.40, 1.24) C: HR 0.67 (0.49, 0.92) **All-cause mortality** A: HR 1.03 (0.45, 2.34) B: HR 0.56 (0.34, 0.90) C: HR 0.69 (0.50, 0.96)	There was no evidence that the effect of dapagliflozin compared with placebo on eGFR decline ≥ 50%, end-stage kidney disease, or kidney or cardiovascular death (*p*-for-interaction = 0.667), kidney composite outcome (*p*-for-interaction = 0.437), cardiovascular outcome (*p*-for-interaction = 0.627), all-cause mortality (*p*-for-interaction = 0.417) was different across the frailty spectrum
Pharmacological intervention — anti-interleukin 1 monoclonal antibody			
**Intervention:** canakinumab (50, 100, or 300 mg) vs placebo **Population:** 9942 patients with stable post-myocardial infarction **Follow-up:** 60 months **Reference:** Orkaby, 2023, 39 countries^13^	34-item frailty index (range, 0–1) A: frailty index (0 to < 0.1) B: frailty index (0.1 ≤ to < 0.2) C: frailty index (0.2 ≤ to 1)	**Incident MACE** A: HR 0.89 (0.73, 1.10) B: HR 0.86 (0.74, 1.00) C: HR 0.87 (0.71, 1.08)	There was no evidence that the effect of canakinumab compared with placebo on incident MACE was different across the frailty spectrum (*p*-for-interaction = NR)
Pharmacological intervention — vaccinations			
**Intervention:** adjuvanted recombinant zoster vaccine vs placebo **Population:** 29,305 adults aged 50 years and older **Follow-up:** 36 months **Reference:** Curran, 2020, 17 countries^32^	41-item frailty index^*^ (range, 0–1) A: frailty index (0 to 0.08) B: frailty index (0.08 < to 0.25) C: frailty index (0.25 < to 1.0)	**Vaccine efficacy**^†^ A: 95.8% B: 90.4% C: 90.2%	There was no evidence that the effect of recombinant zoster vaccine compared with placebo on herpes zoster was different across the frailty spectrum (*p*-for-interaction = NR)
**Intervention:** 23-valent polysaccharide vaccine vs 23-valent polysaccharide vaccine with 7-valent pneumococcal conjugate vaccine **Population:** 312 frail hospitalized adults aged 60 years and older **Follow-up:** 12 months **Reference:** Maclntyre 2014, Australia^33^ Maclntyre 2019, Australia^34^	40-item frailty index (range, 0–1) A: frailty index (0 to 0.250) B: frailty index (0.250 < to 0.375) C: frailty index (0.375 < to 1)	**Serotype 4 IgG (mg/mL)** (12 months^†^) A: 1.0 vs 1.5 B/C: 0.8 vs 0.6 (72 months) A: 1.6 vs 1.7 B/C: 1.2 vs 0.6 **Serotype 18C IgG (mg/mL)** (12 months^†^) A: 5.0 vs 5.4 B/C: 2.9 vs 2.7 (72 months) A: 6.6 vs 5.9 B/C: 5.5 vs 2.3 **Serotype 19F IgG (mg/mL)** (12 months^†^) A: 2.8 vs 3.9 B/C: 2.3 vs 1.8	There was no evidence that the response of 23-valent polysaccharide vaccine with 7-valent pneumococcal conjugate vaccine compared with 23-valent polysaccharide vaccine was different across the frailty spectrum (*p*-for-interaction = NR)
Non-pharmacological intervention — diabetes management			
**Intervention:** intensive lifestyle intervention vs diabetes support and education **Population:** 5145 adults aged 45–76 years with type 2 diabetes and overweight or obesity **Follow-up:** 118 months **Reference:** Simpson, 2021, US^11^	38-item frailty index (range, 0–1) A: frailty index (0 to < 0.178) B: frailty index (0.178 ≤ to < 0.230) C: frailty index (0.230 ≤ to 1)	**Cardiovascular events:** A: RR 0.73 (0.55, 0.98) B: RR 0.97 (0.72, 1.17) C: RR 1.15 (0.94, 1.42)	Intensive lifestyle intervention was associated with lower cardiovascular events compared with diabetes support and education in patients with frailty index < 0.178, not in patients with frailty index ≥ 0.178 (*p*-for-interaction = 0.01)
Non-pharmacological intervention — physical activity and exercise			
**Intervention:** chair yoga vs health education **Population:** 112 adults aged 65 years or older with lower extremity osteoarthritis **Follow-up:** 8 weeks **Reference:** Park, 2020, US^35^	82-item frailty index (range, 0–1) A: quartile 1 (mean frailty index 0.39) B: quartile 2 (mean frailty index 0.43) C: quartile 3 (mean frailty index 0.47) D: quartile 4 (mean frailty index 0.57)	**WOMAC pain score:** NR **Pain interference:** NR	Chair yoga was associated with lower WOMAC pain score (*p*-for-interaction = 0.02) and pain interference (*p*-for-interaction = 0.01) compared with health education in patients with frailty index quartiles 3 and 4, not in patients with frailty index quartiles 1 and 2
**Intervention:** aerobic exercise training vs usual care **Population:** 2130 stable patients with HFrEF **Follow-up:** 36 months **Reference:** Pandey, 2022, United States, Canada, and France^37^	36-item frailty index (range, 0–1) A: frailty index (0 to ≤ 0.21) B: frailty index (0.21 < to 1)	**Composite of all-cause hospitalization or all-cause mortality** A: HR 1.04 (0.87, 1.25) B: HR 0.83 (0.72, 0.95)	Aerobic exercise training was associated with lower rates of composite of all-cause hospitalization or all-cause mortality compared with usual care in patients with frailty index > 0.21 (*p*-for-interaction = NR)
**Intervention:** Intensive exercise intervention vs Usual care **Population:** 323 adults admitted to an acute care ward **Follow-up:** 58 months **Reference:** Pérez-Zepeda, 2022, Spain^12^	63-item frailty index (range, 0–1) A: frailty index (0 to < 0.2) B: frailty index (0.2 ≤ to < 0.29) C: frailty index (0.3 ≤ to 1)	**Mortality** A: HR 0.9 (0.18, 4.3) B: HR 0.4 (0.08, 2.6) C: HR 0.8 (0.2, 2.9) **Refer to **Appendix Table	There was no evidence that the effect of intensive exercise compared with usual care on mortality was different across the frailty spectrum (*p*-for-interaction = NR)
**Intervention:** physical activity vs Health education **Population:** 1635 community-dwelling participants **Follow-up:** 2 years **Reference:** Quach, 2022, US^38^	44-item frailty index A: frailty index centered in 0.05 B: frailty index centered in 0.10 C: frailty index centered in 0.15 D: frailty index centered in 0.20 E: frailty index centered in 0.25 F: frailty index centered in 0.30 G: frailty index centered in 0.35 H: frailty index centered in 0.40 I: frailty index centered in 0.45	**Major mobility disability** A: HR 0.87 (0.64, 1.17) B: HR 0.84 (0.66, 1.06) C: HR 0.81 (0.67, 0.98) D: HR 0.78 (0.66, 0.92) E: HR 0.75 (0.62, 0.90) F: HR 0.72 (0.58, 0.90) G: HR 0.70 (0.53, 0.92) H: HR 0.67 (0.47, 0.95) I: HR 0.65 (0.43, 0.98)	Physical activity was associated with lower rates of major mobility disability compared with health education in patients with centered frailty index ≥ 0.15 (*p*-for-interaction = NR)
Non-pharmacological intervention — others			
**Intervention:** frailty screening vs frailty screening + nurse-led care program vs usual care **Population:** 3092 adults aged 60 and older **Follow-up:** 12 months **Reference:** Bleijenberg, 2016, Netherlands^36^	50-item frailty index (range, 0–1) A: frailty index (0 to < 0.2) B: frailty index (0.2 ≤ to 1)	**Modified Katz-15 score:** NR	There was no evidence that the effect of frailty screening alone or with nurse-led care program compared with usual care on modified Katz-15 score was difference across the frailty spectrum (*p*-for-interaction = NR)
Multicomponent intervention			
**Intervention:** GA-intervention^a^ vs usual-care **Population:** 541 patients with incurable cancer and impairment on ≥ 1 GA domain **Follow-up:** NR **Reference:** Gilmore, 2021, US^39^	50-item frailty index (range, 0–1) A: frailty index (0 ≤ to < 0.2) B: frailty index (0.2 ≤ to < 0.35) C: frailty index (0.35 ≤ to 1)	**Conversations** A: adjusted mean difference 3.27 (1.68, 4.86) B: adjusted mean difference 3.75 (2.32, 5.18) C: adjusted mean difference 4.03 (2.50, 5.56) **Concerns** **Acknowledged** A: adjusted mean difference 2.08 (1.04, 3.12) B: adjusted mean difference 1.87 (0.97, 2.77) C: adjusted mean difference 2.27 (1.29, 3.25) **Concerns** **Addressed** A: adjusted mean difference 2.03 (0.87, 3.19) B: adjusted mean difference 2.13 (1.05, 3.21) C: adjusted mean difference 2.19 (1.07, 3.31)	There was no evidence that the effect of GA-intervention compared with usual care on conversations (*p*-for-interaction = 0.6111), concerns acknowledged (*p*-for-interaction = 0.7397), concerns addressed (*p*-for-interaction = 0.9403) was different across the frailty spectrum
**Intervention:** pharmacist-led deprescribing intervention^b^ vs usual care **Population:** 363 older adults living in the community **Follow-up:** 6 months **Reference:** Jamieson, 2023, New Zealand^40^; Nishtala, 2023, New Zealand^41^	15-item frailty index (range, 0–1) A: low-frailty B: medium-frailty C: high-frailty	**Changes in DBI 0.5 or more** A: difference 9.85% (− 3.94%, 23.48%) B: difference − 5.45% (− 17.25%, 6.35%) C: difference − 5.13% (− 22.08%, 11.82%) **ACB** A: mean change − 0.02 (− 0.65, 0.18) B: mean change 0.05 (− 0.28, 0.38) C: mean change 0.08 (− 0.40, 0.56)	There was no evidence that the effect of pharmacist-led deprescribing intervention compared with usual care on changes in DBI 0.5 or more, ACB was different across the frailty spectrum (*p*-for-interaction = NR)

**Abbreviations:** *HR*, hazard ratio; *NR*, not reported; *RR*, relative risk; *MD*, mean difference; *HFpEF*, HF with preserved ejection fraction; *HF*, heart failure; *WOMAC*, Western Ontario and McMaster Universities; *CKD*, chronic kidney disease; *eGFR*, estimated glomerular filtration rate; *MACE*, major adverse cardiovascular events; *HFrEF*, heart failure with reduced ejection fraction; *GA*, geriatric assessment; *DBI*, Drug Burden Index; *ACB*, anticholinergic cognitive burden

^*^Data were extracted from supplemental files

^†^Data were extracted by GetData Graph Digitizer software

^a^The geriatric assessment (GA) is a validated multidisciplinary evaluation of the functional, psychosocial, physical, and cognitive abilities of older adults, as well as their comorbidities and medication use. Only patients and oncologists in the GA-intervention arm received a summary of the GA plus a list of GA-guided recommendations to address specific impairments (i.e., GA-interventions)

^b^The pharmacist recorded all medications and supplements the person was taking. However, the pharmacist did not have access to the participants’ clinical notes or medication records and all clinical decision-making, including prescribing, remained with the GP

**Table 2:** Results from 17 Randomized Controlled Trials using Frailty Phenotype

Intervention, population, reference	Frailty assessment	Frailty-specific results	Interpretation
Pharmacological intervention — antiplatelet medications			
**Intervention:** prasugrel vs clopidogrel **Population:** 9326 adults aged 65 years or older with unstable angina **Follow-up:** 3 months **Reference:** White, 2016, 52 countries^42^	Fried frailty criteria (range, 0–5) A: frailty phenotype (0) B: frailty phenotype (1 to 2) C: frailty phenotype (3 to 5)	**Cardiovascular death, myocardial infarction, or Stroke** A: HR 0.90 (0.77, 1.06) B: HR 1.34 (1.04, 1.73) C: HR 0.89 (0.54, 1.46)	Prasugrel was associated with higher rates of composite of cardiovascular death, myocardial infarction, or stroke compared with clopidogrel in patients with 1–2 components of the frailty phenotype (*p*-for-interaction = 0.032)
Pharmacological intervention — anticoagulants			
**Intervention:** very-low-dose edoxaban vs placebo **Population:** 984 patients with atrial fibrillation aged 80 years or older **Follow-up:** 4 months **Reference:** Akashi, 2022, Japan^43^	Fried frailty criteria (range, 0–5) A: frailty phenotype (0 to < 3) B: frailty phenotype (3 to 5)	**Stroke or systemic embolism** A: HR 0.24 (0.10, 0.59) B: HR 0.35 (0.14, 0.87) **All-cause death** A: HR 1.46 (0.85, 2.51) B: HR 0.72 (0.45, 1.18) **Net clinical composite outcome** A: HR 0.99 (0.65, 1.51) B: HR 0.77 (0.50, 1.17)	There was no evidence that the effect of edoxaban compared with placebo on stroke or systemic embolism (*p*-for-interaction = 0.55), all-cause death (*p*-for-interaction = 0.06), and net clinical composite outcome was different across the frailty spectrum (*p*-for-interaction = 0.42)
Pharmacological intervention — osteoporosis medications			
**Intervention:** strontium ranelate vs placebo **Population:** 5082 older women with osteoporotic **Follow-up:** 3 years **Reference:** Rolland, 2010, 12 countries^16^	Fried frailty criteria (range, 0–5) A: frailty phenotype (0) B: frailty phenotype (1 to 2) C: frailty phenotype (3 to 5)	**Vertebral fracture** A: HR 0.70 (0.57, 0.86) B: HR 0.55 (0.46, 0.67) C: HR 0.42 (0.24, 0.74)	There was no evidence that the effect of strontium ranelate compared with placebo on vertebral fracture was different across the frailty spectrum (*p*-for-interaction = 0.11)
Pharmacological intervention — androgen medications			
**Intervention:** testosterone gel vs placebo **Population:** 262 men aged 65 years or older **Follow-up:** 6 months **Reference:** Srinivas-Shankar, 2010, UK^15^	Fried frailty criteria (range, 0–5) A: frailty phenotype (1) B: frailty phenotype (2) C: frailty phenotype (3) D: frailty phenotype (4)	**IME-PT** Adjusted difference: 9.48 (− 4.05, 23.02)	There was no evidence that testosterone gel compared with placebo on IME-PT was different across the frailty spectrum (*p*-for-interaction = 0.68)
Non-pharmacological intervention — physical activity and exercise			
**Intervention:** individual training (Nintendo Wii Fit Plus) vs physical activity education **Population:** 30 older adults aged 71–92 years **Follow-up:** 30 days **Reference:** Gomes, 2018, Brazil^14^	Fried frailty criteria (range, 0–5) A: frailty phenotype, 1–2 B: frailty phenotype, ≥ 3	**Mini-BESTest score at final and follow-up assessments**^†^ (Final) A: mean 17.6 (14.8, 20.5) vs mean 19.1 (17.4, 20.9) B: mean 19.6 (16.6, 22.6) vs mean 15.8 (13.6, 18.0) (Follow-up) A: mean 16.9 (14.1, 19.7) vs mean 19.0 (17.2, 20.8) B: mean 17.6 (14.3, 20.9) vs mean 15.7 (13.0, 18.3) **FGA at final and follow-up assessments**^**†**^ (Final) A: mean 19.5 (17.2, 21.9) vs mean 18.2 (16.2, 20.3) B: mean 18.0 (16.2, 19.8) vs mean 15.8 (13.5, 18.0) (Follow-up) A: mean 19.9 (17.4, 22.4) vs mean 19.8 (17.8, 21.7) B: mean 19.0 (17.1, 20.9) vs mean 17.6 (15.4, 19.9)	There was no evidence that the effect of individual training (Nintendo Wii Fit Plus) compared with physical activity education on Mini-BESTest score (*p*-for-interaction > 0.05), and FGA (*p*-for-interaction > 0.05) was different across the frailty spectrum
**Intervention:** LSRT vs HSRT vs control **Population:** 60 adults aged 60 years and older **Follow-up:** NR **Reference:** Coelho-junior, 2021, Brazil^45^	Fried frailty criteria (range, 0–5) A: Frailty phenotype (1 to 2) B: Frailty phenotype (3 to 5)	Refer to Appendix Table	There was no evidence that the effect of LSRT and HSRT compared with control on physical performance was different across the frailty spectrum (*p*-for-interaction = NR)
**Interaction:** functional walking vs usual pattern of activities **Population:** 278 adults aged 63–98 years **Follow-up:** 52 weeks **Reference:** Faber, 2006, Netherland^46^	Fried frailty criteria (range, 0–5) A: frailty phenotype (0) B: frailty phenotype (1 to 2) C: frailty phenotype (3 to 5)	**POMA** B: mean 1.1 (0.4, 1.8) C: mean 0.5 (− 0.6, 1.7) **PPS** B: mean 0.7 (0.3, 1.2) C: mean − 0.7 (− 1.3, − 0.0) **Time to first fall** B: HR 0.62 (0.29, 1.33) C: HR 2.95 (1.64, 5.32)	There was no evidence that the effect of functional walking compared with usual pattern of activities on PPS was different across the frailty spectrum (*p*-for-interaction = NR) Functional walking was associated with higher risks of fall compared with usual pattern of activities in patients with 3 or more frailty phenotypes, but not in patients with 1–2 components of the frailty phenotype (*p*-for-interaction = 0.002)
**Intervention:** tailored, progressive physical rehabilitation vs usual care **Population:** 349 adults aged 60 years or older with heart failure **Follow-up:** 3 months **Reference:** Kitzman, 2021, US^47^	Modified Fried frailty criteria (range, 0–5) A: frailty phenotype (0) B: frailty phenotype (1 to 2) C: frailty phenotype (3 to 5)	**SPPB score** (between-group difference in mean score, 95% CI) A or B: 0.7 (− 0.1, 1.5) C: 2.1 (1.3, 2.8)	There was no evidence that the effect of tailored, progressive physical rehabilitation compared with usual care on SPPB score was different across the frailty spectrum (*p*-for-interaction = NR)
**Intervention:** physical rehabilitation intervention vs attention control **Population:** 337 patients 60 years and older hospitalized for acute decompensated heart failure **Follow-up:** 3 months **Reference:** Pandey, 2023, US^37^	Modified Fried frailty criteria (range, 0–5) A: frailty phenotype (1 to 2) B: frailty phenotype (3 to 5)	**SPPB score** A: effect size 0.8 (− 0.1, 1.6) B: effect size 2.1 (1.3, 2.9)	Physical rehabilitation intervention was associated with greater improvement in SPPB score compared with attention control in patients with 3 or more frailty phenotypes, but not in patients with 1–2 components of the frailty phenotype (*p*-for-interaction = 0.03)
**Intervention:** physical exercise vs usual care **Population:** 299 adults aged 65 years and older **Follow-up:** 24 months **Reference:** Suikkanen, 2020, Finland^49^	Modified Fried frailty criteria (range, 0–5) A: frailty phenotype (1 to 2) B: frailty phenotype (3 to 5)	**Days at home** A: IRR 1.03 (0.96, 1.11) B: IRR 1.04 (0.96, 1.12)	There was no evidence that the effect of physical exercise compared with usual care on days at home was different across the frailty spectrum (*p*-for-interaction = NR)
**Intervention:** resistance-type exercise training vs no exercise training **Population:** 127 adults aged 65 years or older with pre-frail or frail **Follow-up:** 24 weeks **Reference:** Tieland, 2015, Netherland^50^	Fried frailty criteria (range, 0–5) A: Frailty phenotype (1 to 2) B: Frailty phenotype (3 to 5)	**Dominant handgrip strength** NR	There was no evidence that the effect of resistance-type exercise training compared with no exercise training on dominant handgrip strength was different across the frailty spectrum (*p*-for-interaction > 0.05)
**Intervention:** moderate-intensity physical activity vs health education **Population:** 1635 adults aged 70–89 years with functional limitations **Follow-up:** 2 years **Reference:** Trombetti, 2018, US^51^	Study of Osteoporotic Fractures Index (range, 0–3) A: frailty phenotype (0 to < 2) B: frailty phenotype (2 to 3)	**MMD**^‡^ A vs B: HR 0.92 vs HR 0.96 **PBD**^‡^ A vs B: HR 0.92 vs HR 0.94	There was no evidence that the effect of moderate-intensive physical activity compared with health education on MMD (*p*-for-interaction = 0.91) and PBD (*p*-for-interaction = 0.64) was different across the frailty spectrum
**Intervention:** walking and nutrition vs walking vs control **Population:** 227 adults aged 65 years and older **Follow-up:** 6 months **Reference:** Yamada, 2015, Japan^52^	Modified Cardiovascular Health Study criteria (range, 0–5) A: frailty phenotype (0 to 2) B: frailty phenotype (3 to 5)	**SMI** A: 1.02% vs 1.11% vs − 0.86% B: 3.16% vs 0.64% vs − 3.87% **IGF-1** A: 21.4% vs 22.5% vs 8.6% B: 31.8% vs 14.5% vs 9.4% **DHEA-S** A: 26.6% vs 18.1% vs 8.4% B: 15.9% vs 19.7% vs − 0.8% **25(OH)D** A: 39.9% vs 32.0% vs 6.1% B: 45.2% vs 33.6% vs − 5.6%	There was no evidence that the effect of walking and nutrition or walking compared with control on SMI, IGF-1, DHEA-S, and 25(OH)D was different across the frailty spectrum (*p*-for-interaction = NR)
Non-pharmacological intervention — diabetes management			
**Intervention:** multimodal intervention^a^ vs usual care **Population:** 964 adults aged over 70 years older adults with type 2 diabetes mellitus and functional impairment **Follow-up:** 12 months **Reference:** Rodriguez-Manas, 2019, 7 European countries^44^	Fried frailty criteria (range, 0–5) A: frailty phenotype (1 to 2) B: frailty phenotype (3 to 5)	**Changes in physical function**: NR	There was no evidence that the effect of multimodal intervention compared with usual care on physical function was different across the frailty spectrum (*p*-for-interaction = 0.49)
Multicomponent intervention			
**Intervention:** ①MI^b^ + n3 PUFA^c^ vs placebo ②n3 PUFA vs Placebo ③MI + placebo vs placebo **Population:** 1680 adults aged 70 years and older without dementia but with subjective memory complaints **Follow-up:** 3 years **Reference:** Tabue-teguo, 2018, France^20^	Fried frailty criteria (range, 0–5) A: frailty phenotype (0) B or C: frailty phenotype (1 to 5)	**3-year TMTA** **Difference of score change** ①Pre-frail, − 4.029 Non-frail, 1.134 ②Pre-frail, 0.246 Non-frail, 3.478 ③Pre-frail, 2.268 Non-frail, 2.951 **3-year TMTB** **Difference of score change** ①Pre-frail, − 4.093 Non-frail, − 1.466 ②Pre-frail, − 6.313 Non-frail, 1.798 ③Pre-frail, 12.891 Non-frail, − 4.297 Refer to Appendix Table for more outcomes	There was no evidence that the effect of MI and n3 PUFA program compared with placebo on cognitive function was different across the frailty spectrum (*p*-for-interaction > 0.05)
**Intervention:** multifactorial, interdisciplinary intervention^d^ vs usual care **Population:** 241 adults aged 70 years and older without severe cognitive impairment **Follow-up:** 1 year **Reference:** Fairhall, 2012, Australia^53^ Fairhall, 2014, Australia^54^	Modified CHS frailty criteria (range 0–5) A: frailty phenotype (3) B: frailty phenotype (4 to 5)	**Life Space Assessment score**^**e**^ (3 months) A: 9.0 (5.3, 12.7) B: 2.0 (− 2.8, 6.8) (12 months) NR **Gait speed** (3 months) NR (12 months) A: 0.01 m/s (− 0.05, 0.07) B: 0.13 m/s (0.04, 0.22) **SPPB**: NR **PPA**: NR	Multifactorial, interdisciplinary intervention was associated with greater life space compared with usual care in patients with 3 components of the frailty phenotype at 3 months (*p*-for-interaction = 0.03), but no longer effective at 12 months (*p*-for-interaction = 0.4) Multifactorial, interdisciplinary intervention was associated with greater gait speed compared with usual care in patients with 4–5 components of the frailty phenotype at 12 months (*p*-for-interaction = 0.03), but not effective at 3 months (*p*-for-interaction = 0.9) There was no evidence that multifactorial, interdisciplinary intervention compared with usual care on SPPB and PPA was different across frailty spectrum (*p*-for-interaction = NR)

**Abbreviations:** *HR*, hazard ratio; *NR*, not reported; *IME-PT*, isometric knee extension peak torque; *MMD*, major mobility disability; *PBD*, persistent mobility disability; *POMA*, performance-oriented mobility assessment; *PPS*, physical performance score; *SPPB*, Short Physical Performance Battery; *LSRT*, low-speed resistance training; *HSRT*, high-speed resistance training; *FGA*, functional gait assessment; *SMI*, skeletal muscle mass index; *IGF-1*, insulin-like growth factor; *DHEA-S*, dehydroepiandrosterone sulfate; *25 (OH)D*, 25-hydroxy vitamin D; *MI*, multidomain intervention; *n3 PUFA*, omega-3 polyunsaturated fatty acids; *CHS*, Cardiovascular Health Study; *PPA*, Physiological Profile Assessment

*p*-for-interaction: an indicator used to infer whether the effect of an intervention on outcome measures is significant at different levels of frailty

^*^Data were extracted from supplemental files

^†^Data were extracted by GetData Graph Digitizer software

^‡^Calculations are based on published data in the literature

^a^A multimodal intervention composed of (i) an individualized and progressive resistance exercise program for 16 weeks; (ii) a structured diabetes and nutritional educational program over seven sessions; and (iii) investigator-linked training to ensure optimal diabetes care

^b^The multidomain intervention (MI) consisted of 2-h group sessions focusing on three domains (cognitive stimulation, physical activity, and nutrition) and a preventive consultation (at baseline, 12 months, and 24 months) and for omega-3 polyunsaturated fatty acids supplementation, participants took two capsules of either placebo or polyunsaturated fatty acids daily

^c^The n-3 PUFA supplementation group consumed a daily dose of DHA (800 mg) and EPA (a maximum amount of 225 mg) for 3 years

^d^Intervention was coordinated by two physiotherapists with extensive relevant experience and delivered by an interdisciplinary team comprising the physiotherapists, a dietician, a geriatrician, a rehabilitation physician and a nurse. Intervention was delivered primarily in the participants’ homes, with additional outpatient appointments (for example, dietician, continence clinic), occupational therapy, and community exercise programs offered as indicated

^e^Mobility during the preceding month was quantified in terms of distance and frequency of travel and degree of independence using the University of Alabama at Birmingham Life Space Assessment. Scored on a continuous scale from 0 to 120, a higher score illustrates greater life space

**Table 3:** Results from 20 Randomized Controlled Trials using Other Frailty Assessment Tools

Intervention, population, reference	Frailty assessment	Frailty-specific results	Interpretation
Pharmacological intervention — myeloma medications			
**Intervention:** lenalidomide vs placebo **Population:** 40 adults with myeloma **Follow-up:** 12.9 months (median) **Reference:** Brioli, 2019, Germany^17^	International Myeloma Working Group geriatric score (range, 0–5) A: class 1 (0) B: class 2 (1) C: class 3 (2 to 5)	**PFS**: NR **OS**: NR	There was no evidence that the effect of lenalidomide compared with placebo on PFS and OS was different across the frailty spectrum (*p*-for-interaction = NR)
**Intervention:** Rd continuous vs Rd18 vs fixed-duration MPT **Population:** 1618 transplant-ineligible adults with newly diagnosed multiple myeloma **Follow-up:** 90 months **Reference:** Facon, 2019, Europe, North America, and the Asia–Pacific region^59^	Simplified ECOG-based frailty assessment (range, 0–7) A: class 1 (0 to 1) B: class 2 (2 to 7)	**PFS** A: HR 0.60 (0.49, 0.75) B: HR 0.75 (0.61, 0.91) **OS** A: HR 0.69 (0.54, 0.88) B: HR 0.84 (0.68, 1.04)	Rd continuous was potentially associated with more prolonged PFS and OS compared with MPT in patients with Simplified ECOG-based frailty assessment < 1 (*p*-for-interaction = NR)
**Intervention:** daratumumab plus lenalidomide/dexamethasone vs lenalidomide/dexamethasone **Population:** 737 patients with transplant-ineligible newly diagnosed multiple myeloma **Follow-up:** 36.4 (median) months **Reference:** Facon, 2022, North America, Europe, the Middle East, and the Asia–Pacific region^60^	Simplified ECOG-based frailty assessment (range, 0–7) A: Class 1 (0 to 1) B: Class 2 (2 to 7)	**Progression-free survival** A: HR 0.48 B: HR 0.003	There was no evidence that the effect of daratumumab plus lenalidomide/dexamethasone compared with lenalidomide/dexamethasone on progression-free survival was different across the frailty spectrum (*p*-for-interaction = NR)
**Intervention:** MPR vs CPR vs Rd (low-dose) **Population:** 654 adults aged 50–89 years with multiple myeloma **Follow-up:** 60 months, 70 months **Reference:** Bringhen, 2020, Italian and Czech Republic^61^ Magarotto, 2016, Italy and Czech Republic^62^	International Myeloma Working Group geriatric score (range, 0–5) A: class 1 (0) B: class 2 (1) C: class 3 (2 to 5)	**PFS** (MPR vs CPR) 60 months A: HR 0.740 (0.506, 1.082) B: HR 0.966 (0.637, 1.466) C: HR 0.731 (0.452, 1.182) 70 months A: HR 0.72 (0.52, 1.00) B: HR 0.80 (0.56, 1.14) C: HR 0.88 (0.57, 1.34) (MPR vs Rd) 60 months A: HR 0.671 (0.461, 0.976) B: HR 0.850 (0.550, 1.313) C: HR 1.030 (0.632, 1.680) 70 months A: HR 0.72 (0.52, 0.99) B: HR 0.82 (0.56, 1.20) C: HR 1.18 (0.78, 1.80)	MPR was potentially associated with more prolonged PFS compared with Rd in fit patients (*p*-for-interaction = NR)
**Intervention:** ixazomib vs placebo **Population:** 706 adults aged 42–90 years with multiple myeloma **Follow-up:** NR **Reference:** Dimopoulos, 2020, 34 countries^64^	Frailty assessment based on 4 components^a^ A: fit/B: unfit/C: frail (NR)	**PFS** A: HR 0.530 (0.387, 0.727) B: HR 0.746 (0.526, 1.058) C: HR 0.733 (0.481, 1.117)	Ixazomib was potentially associated with more prolonged PFS compared with placebo in fit patients, but not in unfit and frail patients (*p*-for-interaction = NR)
Pharmacological intervention — vaccinations			
**Intervention:** high-dose inactivated influenza vaccine vs standard-dose vaccine **Population:** 31,989 medically stable adults **Follow-up:** 6–8 months **Reference:** DiazGranados, 2015, US and Canada^18^	Frailty-associated conditions (0–14 conditions^b^) A: class 1 (0) B: class 2 (1) C: class 3 (2) D: class 4 (3 to 14)	**Laboratory-confirmed influenza caused by any viral type/subtype (regardless of similarity to the vaccine)** A: 34.0 (− 7.9, 60.2) B: 27.5 (0.4, 47.4) C: 23.9 (− 9.0, 47.2) D: 16.0 (− 16.3, 39.4)	There was no evidence that the effect of high-dose inactivated influenza vaccine compared with standard-dose vaccine on laboratory-confirmed influenza caused by any viral type/subtype (regardless of similarity to the vaccine) was different across the frailty spectrum (*p*-for-interaction = 0.838)
Non-pharmacological intervention — radiation therapy			
**Intervention:** 1-week course radiation therapy vs 3-week course radiation **Population:** 61 adults aged 65 years and older with glioblastoma **Follow-up:** 2.5 years **Reference:** Guedes de Castro, 2017, 10 countries^65^	Karnofsky Performance Status (range, 50–100%) A: class 1 (80–100%) B: class 2 (50–70%)	**OS** A: 8.0 months (5.9, 10.0) vs 8.0 months (5.3, 10.3) B: 7.5 months (5.3, 9.7) vs 6.7 months (4.5, 8.9)	There was no evidence that the effect of short-course radiation therapy compared with 3-week course radiation on OS was different across the frailty spectrum (*p*-for-interaction = NR)
Non-pharmacological intervention — surgical procedures			
**Intervention:** anterior minimally invasive hemiarthroplasty vs lateral Hardinge hemiarthroplasty **Population:** 190 adults aged 60 years and older with femoral neck fractures **Follow-up:** 12 months **Reference:** Saxer, 2018, Switzerland^66^	(18-(Functional Independence Measure-18)/6 + Charlson Index + Medication score)/36 (range, 0–1) A: frailty index (0 to ≤ 0.25) B: frailty index (0.25 < to 1)	**Timed up and go duration:** NR	There was no evidence that anterior minimally invasive hemiarthroplasty compared with lateral Hardinge hemiarthroplasty on timed up and go duration was different across the frailty spectrum (*p*-for-interaction = NR)
Non-pharmacological intervention — physical activity and exercise			
**Intervention:** high-dose exercise program vs low-dose exercise program vs no exercise **Population:** 110 adults aged over 65 years with sedentary behavior **Follow-up:** 12 weeks **Reference:** Kaushal, 2019, Canada^67^	Combined Fried frailty phenotype, modified Physical Performance Test, and frailty index Frail: meeting at least 2 of the 3 methods	**HR-QOL capacity**: NR	Higher dose exercise program was associated with greater improvement in capacity HR-QOL compared with no exercise in patients meeting at least 2 of the 3 frailty assessments (*p*-for-interaction = 0.037)
**Intervention:** physical activity vs health education **Population:** 1635 adults aged 70–89 years **Follow-up:** 3.5 years **Reference:** Newman, 2016, US^68^	SPPB score (range, 0–12) A: class 1 (0 to < 8) B: class 2 (8 to 9)	**Total cardiovascular disease event rates** A: HR 0.76 (0.52, 1.10) B: HR 1.59 (1.09, 2.30)	Physical activity was associated with higher total cardiovascular event rates compared with successful aging in patients with SPPB score 8 to 9, but not in patients with SPPB < 8 (*p*-for-interaction = 0.006)
**Intervention:** physical activity vs health education program **Population:** 1623 older persons with mobility limitations **Follow-up:** 24 months **Reference:** Custodero, 2023, US^63^	Study of Osteoporotic Fractures frailty index (range, 0–3 criteria) A: class 1 (0 to 1) B: class 2 (2 to 3)	**400-m gait-speed** (6-month) A: mean difference 0.029 (0.017, 0.041) B: mean difference 0.027 (− 0.001, 0.055) (12-month) A: mean difference 0.023 (0.011, 0.035) B: mean difference 0.014 (− 0.015, 0.042) (24-month) A: mean difference 0.023 (0.010, 0.035) B: mean difference 0.010 (− 0.020, 0.039) **4-m gait-speed** (6-month) A: mean difference − 0.004 (− 0.018, 0.011) B: mean difference − 0.011 (− 0.041, 0.018) (12-month) A: mean difference − 0.002 (− 0.016, 0.013) B: mean difference − 0.008 (− 0.039, 0.012) (24-month) A: mean difference − 0.001 (− 0.014, 0.016) B: mean difference − 0.010 (− 0.022, 0.41)	There was no evidence that the effect of physical activity compared with health education on 400-m gait-speed, 4-m gait-speed was different across the frailty spectrum (*p*-for-interaction = NR)
Non-pharmacological intervention — psychosocial intervention			
**Intervention:** psychosocial intervention vs standard educational material on stroke recovery **Population:** 291 adults aged over 45 years with stroke **Follow-up:** 6 months (47 months for mortality) **Reference:** Ertel, 2007, US^69^	Summary frailty index^c^ (range, 0–5): A: frailty index (0 to ≤ 3) B: frailty index (4 to 5)	**Instrumental activities of daily living score** (range, 0–14) A: MD 1.11 (*p* = 0.01) B: MD − 0.94 (*p* = 0.09) **Physical performance score** (range, 1–25) A: MD 1.41 (*p* = 0.11) B: MD − 1.56 (*p* = 0.10) **Global cognitive function score (standardized):** A: MD 0.09 (*p* = 0.25) B: MD 0.01 (*p* = 0.93) **All-cause mortality:** A: HR 0.40 (*p* = 0.03) B: HR 1.34 (*p* = 0.27)	Psychosocial intervention was associated with improvement in instrumental activities of daily living (*p*-for-interaction < 0.01), possible improvement in physical performance (*p*-for-interaction = 0.02), and reduced mortality (*p*-for-interaction = 0.01) compared with standard educational material in patients with frailty index 0–3, not in patients with frailty index 4–5
Non-pharmacological intervention — others			
**Intervention:** home-based physiotherapy vs hospital-based treatment rehabilitation **Population:** 451 adults aged 62–81 years with stroke **Follow-up:** 6 months **Reference:** Gladman, 1995, UK^70^	An operational definition of frailty based on: (1) Age > 80 years (2) Living alone (3) Previous disability (4) Previous Functional Ambulation Category score < 5 (5) Not scoring above 6/10 on the AMTS (6) Hospital stay > 1 month or discharge Barthel < 15/20 Frail: 2 or more criteria	**Function measures** (median Barthel score; median improvement in Barthel score; being able to walk outside): NR	There was no evidence that the effect of home-based physiotherapy compared with hospital-based treatment rehabilitation on function measures was different across frailty spectrum (*p*-for-interaction = NR)
**Intervention:** embrace^d^ vs usual care **Population:** 1456 adults aged 75 years and older **Follow-up:** 12 months **Reference:** Spoorenberg, 2018, Netherlands^55^ Uittenbroek, 2017, Netherlands^56^	INTERMED-E-SA (range, 0–20) and GFI (range, 0–15) A: class 1 (INTERMED-E-SA: 16 to 20) B: class 2 (INTERMED-E-SA: 0 to < 16 and GFI: 5 to 15) C: class 3 (INTERMED-E-SA: 0 to < 16 and GFI: 0 to < 5)	**EQ-5D-3L** A: effect size *d* = 0.07 B: effect size *d* = 0.16 C: effect size *d* = 0.03 **PAIEC total score** A: 0.44 (0.01, 0.87) B: B 0.89 (0.42, 1.37) C: B 0.13 (− 0.07, 0.33) Refer to Appendix Table for additional measures	There was no evidence that the effect of embrace compared with usual care on the domains of health and wellbeing was different across the frailty spectrum (*p*-for-interaction = NR) Embrace was associated greater improvement in quality of life compared with usual care in patients with INTERMED-E-SA ≥ 16, and patients with INTERMED-E-SA < 16 and GFI 5 to 15 (*p*-for-interaction = NR)
**Intervention:** home-based intervention program^e^ vs educational program **Population:** 188 adults aged 75 years and older with physical frailty **Follow-up:** 1 year **Reference:** Gill, 2002, US^58^	Rapid gait test and chair stand test A: class 1 (meeting 1 of 2 tests) B: class 2 (meeting both tests)	**Disability score (adjusted)** (range, 0–16 points) A: change 53% (*p* = 0.005) B: change 16% (*p* = 0.5)	Home-based intervention program was associated with lower disability score compared with educational program in patients meeting 1 of 2 frailty assessments (*p*-for-interaction < 0.001 at 7 months and *p*-for-interaction = 0.005 at 12 months)
**Intervention:** HF program + home telemedicine^f^ vs HF program **Population:** 178 patients aged 40–92 years with heart failure **Follow-up:** 6 months **Reference:** Comin-Colet, 2015, Spain^71^	Frailty defined as: age ≥ 90 years OR age 85–89 needing caregiver OR moderate to severe dependency for ADL (Barthel Index < 90) at any age OR moderate to severe cognitive impairment according to the Pfeiffer test at any age	**Number of non-fatal HF events** No frailty: HR 0.33 (0.17, 0.64) Frailty: HR 0.41 (0.16, 1.06)	There was no evidence that the effect of HF program and home telemedicine compared with HF program on non-fatal HF events was different across the frailty spectrum (*p*-for-interaction = 0.838)
Multicomponent intervention			
**Intervention:** DMP vs usual care **Population:** 173 adults aged over 70 years with heart failure **Follow-up:** 2 years **Reference:** Pulignano, 2010, Italy^19^	Modified frailty score^g^ (range, 1–6) A: class 1 (1) B: class 2 (2) C: class 3 (3) D: class 4 (4 to 6)	**Death and/or heart failure admissions** A: HR 2.506 (0.459, 13.687) B: HR 0.441 (0.226, 0.859) C: HR 0.426 (0.195, 0.932) D: HR 0.369 (0.153, 0.891) **All-cause admissions** A: HR 1.905 (0.723, 5.024) B: HR 0.490 (0.268, 5.894) C: HR 0.285 (0.124, 0.650) D: HR 0.467 (0.200, 1.089)	There was no evidence that the effect of DMP compared with usual care on death and/or heart failure admissions was different across the frailty spectrum (*p*-for-interaction = 0.208) DMP was associated with lower all-cause admissions compared with usual care in patients with greater frailty scores (*p*-for-interaction = 0.0178)
**Intervention:** POC approach^h^ vs usual care **Population:** 346 community dwelling frail adults aged 70 years and older **Follow-up:** 24 months **Reference:** Metzelthin, 2013, Netherland^57^	GFI (range, 0–15) A: class 1 (5 to 6) B: class 2 (7 to 14)	**Groningen Activity Restriction Scale (range total scale 18–78) at 6, 12, and 24 months** NR	There was no evidence that the effect of POC approach compared with usual care on Groningen Activity Restriction Scale was different across the frailty spectrum (*p*-for-interaction > 0.05)

**Abbreviations:** *HR*, hazard ratio; *NR*, not reported; *MPR*, melphalan-prednisone-lenalidomide; *CPR*, cyclophosphamide-prednisone-lenalidomide; *Rd*, lenalidomide and dexamethasone; *Rd18*, lenalidomide and dexamethasone for 18 cycles; *MPT*, melphalan + prednisone + thalidomide; *ECOG*, Eastern Cooperate Oncology Group; *PFS*, progression-free survival; *OS*, overall survival; *RT*, radiation therapy; *HR-QOL*, health-related quality of life; *SPPB*, Short Physical Performance Battery; *INTERMED-E-SA*, INTERMED for the Elderly Self-Assessment; *GFI*, Groningen Frailty Indicator; *EQ-5D-3L*, EuroQol-5D three-level version; *PAIEC*, Patient Assessment of Chronic Illness Care; *HF*, heart failure; *ADL*, activity of daily living; *AMTS*, a mental test score; *POC*, prevention of care; *DMP*, hospital-based disease management programs

**Notes:**

^*^Data were extracted from supplemental files

^†^Data were extracted by GetData Graph Digitizer software

^a^Patients’ frailty status was classified as fit, unfit, or frail on the basis of four components: age, the Katz Index of Independence in Activities of Daily Living, the Lawton Instrumental Activities of Daily Living Scale, and the Charlson Comorbidity Index Scoring System

^b^Vision loss, hearing loss, impaired mobility, difficulty toileting, difficulty bathing, difficulty dressing, difficulty grooming, difficulty going out, skin problems, resting tremor, changes in sleep, urinary complaints, gastrointestinal problems, and hypertension

^c^A summary frailty index combing measures of physical and mental functioning: depressive symptoms, Mini-Mental State Exam, NIH Stroke Scale, rehabilitation days, and number of pre-existing conditions

^d^Embrace (in Dutch: SamenOud [ageing together]) is a person-centered and integrated care service for community-living older adults. A multidisciplinary care team consisting of the older adults’ GP, a nursing home physician, and two case managers (district nurse and social worker) provides care and support to older adults

^e^A physical therapist assessed each participant for potential impairments in physical abilities and assessed the participant’s home environment. Detailed algorithms and decision rules were developed to link the results of the assessment with the recommended interventions. The program was designed to include an average of 16 visits over a 6-month period

^f^A comprehensive solution for the care and monitoring of chronic patients, modelled and tested in patients with CHF that enables the provision of multichannel service and patient tracking through patient monitoring of biometric data (weight, heart rate, and blood pressure), symptoms reporting (seven questions to capture worsening symptoms of the cardiac condition, mainly worsening heart failure, and one question to capture general worsening), generation and management of warning alarms (biometrics out of range), and alerts (information related to the function of the household devices)

^g^Combining five domains of functioning (age over 80 years, cognitive impairment defined as a MMSE score 24 or less, reduced mobility, urinary incontinence, and physical impairment defined as a NYHA functional class III–IV) into six stages of increasing impairment. Frailty score 1 included patients less than 80 years in NYHA class II and without impairments in mobility, continence, or cognitive function. Frailty scores 2–6 included patients with the presence of one, two, three, four, or all of the specific items, respectively

^h^Within the PoC approach, the general practitioner and practice nurse cooperate closely with occupational and physical therapists. If needed, other inpatient and outpatient healthcare professionals, such as a pharmacist or a geriatrician, are involved as well

### Quality of the Included Trials

Most of the included articles have low likelihood of bias. Risk of bias related to blinding of participants and personnel and incomplete outcome data were identified in 18 and 12 trials, respectively. The risk of bias for each included trial is shown in both the Appendix Figure and Table.

### Evaluation of Treatment Effect by Deficit Accumulation Frailty Index

#### Pharmacologic Interventions

The included 15 trials assessed whether the effect of the following treatments was different by frailty levels: anticoagulants,^21^ antihypertensives,^22–26^ heart failure (HF) medication,^27–31^ anti-interleukin 1 monoclonal antibody,^13^ and vaccinations.^32–34^

##### Anticoagulants

In a trial of 20,867 adults with atrial fibrillation conducted over 34 months across 46 countries,^21^ edoxaban was associated with lower rates of major bleeding compared with warfarin in patients with frailty index 0.12 to <0.36 (edoxaban 60 mg) and patients with frailty index <0.36 (edoxaban 30 mg), but not in patients with frailty index <0.12 (edoxaban 60 mg) and frailty index 0.36 to <1.0 (edoxaban 30 mg or 60 mg) (*p*-for-interaction=not reported [NR]). There was a significant treatment effect of edoxaban compared with warfarin on stroke or systemic embolism, but the treatment effect was not different across the frailty spectrum (*p*-for-interaction=NR).

##### Antihypertensives

There was a significant treatment effect of antihypertensive medication reduction compared with usual care on systolic blood pressure control,^22^ indapamide ± perindopril compared with placebo on stroke and cardiovascular events.^24^ However, there was no significant treatment effect of intensive systolic blood pressure control compared with standard control on syncope and falls,^23^ indapamide ± perindopril compared with placebo on all-cause mortality^24^. Furthermore, five trials^22–26^ have concluded that the effects of antihypertensive medication reduction,^22^ intensive blood pressure control,^23, 25, 26^ and indapamide ± perindopril^24^ compared with control treatment, were similar across frailty levels for cardiovascular events,^24, 25^ stroke,^24^ all-cause mortality,^24, 25^ systolic blood pressure control,^22^ change in systolic blood pressure,^22^ syncope,^23^ hypotension,^23^ falls,^23^ and cerebral blood flow.^26^

##### HF Medications

Sacubitril/valsartan was associated with lower rates of HF hospitalization or cardiovascular death (*p*-for-interaction = 0.002) and HF hospitalization (*p*-for-interaction = 0.001) in patients with a higher frailty index (0.311 to 1), but not in patients with frailty index <0.311.^28^ In a trial of 4742 patients with HF with reduced ejection fraction and elevated natriuretic peptide,^29^ dapagliflozin was associated with lower rates of cardiovascular death (*p*-for-interaction=NR) and all-cause death (*p*-for-interaction=NR) compared with placebo in patients with frailty index ≤0.210, but not in patients with frailty index >0.210. Besides, dapagliflozin was associated with lower rates of cardiovascular outcome (*p*-for-interaction=NR) compared with placebo in patients with frailty index ≤0.210 or frailty index ≥0.311,^29, 31^ but not in patients with frailty 0.210 to <0.311. Another trial found dapagliflozin was associated with lower rates of HF hospitalization or cardiovascular death (*p*-for-interaction=NR) compared with placebo in patients with frailty index <0.311, but not in patients with frailty index ≥0.311.^30^ There was significant treatment effect of spironolactone compared with placebo^27^ on HF hospitalization or cardiovascular death, but the treatment effect was not different across frailty spectrum (*p*-for-interaction = 0.40).

##### Vaccinations

There was significant treatment effect of adjuvanted recombinant zoster vaccine compared with placebo,^32^ 23-valent polysaccharide vaccine compared with 23-valent polysaccharide vaccine with 7-valent pneumococcal conjugate vaccine on serotype 19F IgG (mg/mL)^34^. There was no significant treatment effect of 23-valent polysaccharide vaccine compared with 23-valent polysaccharide vaccine with 7-valent pneumococcal conjugate vaccine on serotype 4 IgG (mg/mL) and serotype 18C IgG (mg/mL)^33^. Vaccine efficacy does not differ across frailty levels for two specific interventions (*p*-for-interaction=NR): adjuvanted recombinant zoster vaccine compared with placebo^32^ and 23-valent polysaccharide vaccine compared with 23-valent polysaccharide vaccine with 7-valent pneumococcal conjugate vaccine.^33, 34 ^

##### Anti-interleukin 1 Monoclonal Antibody

Canakinumab had a significant treatment effect on incident major adverse cardiovascular events compared with placebo, but the effect was not different across frailty levels (*p*-for-interaction=NR).^13^

#### Non-pharmacological Intervention

Six trials assessed whether the effect of following treatment was different by frailty levels: diabetes management,^11^ physical activity and exercise,^12, 35, 37, 38^ and other.^36^

##### Diabetes Management

In a trial of 5145 adults with type 2 diabetes and overweight or obesity conducted over 118 months in the US,^11^ intensive lifestyle intervention was associated with lower cardiovascular events compared with diabetes support and education in patients with frailty index <0.178, not in patients with frailty index ≥0.178 (*p*-for-interaction = 0.01).

##### Physical Activity and Exercise

Increasing frailty index was associated with greater reductions in pain score (*p*-for-interaction = 0.02) and pain interference (*p*-for-interaction = 0.01) with chair yoga compared with health education.^35^ Aerobic exercise training was associated with lower rates of composite of all-cause hospitalization or all-cause mortality compared with usual care in patients with frailty index >0.21, but not in patients with frailty index ≤0.21 (*p*-for-interaction=NR).^37^ Similarly, physical activity was associated with lower rates of major mobility disability compared with health education in patients with frailty index ≥0.15, but not in patients with frailty index <0.15 (*p*-for-interaction=NR)^38^. There was significant treatment effect of intensive exercise compared with usual care on mortality, but the effect was not different across the frailty spectrum (*p*-for-interaction=NR).^12^

##### Other

In a trial conducted in the Netherlands,^36^ involving 3092 adults aged 60 and older over a 12-month follow-up, there was evidence to suggest that the effect of frailty screening alone or when combined with a nurse-led care program compared with usual care was beneficial on the modified Katz-15 function score, but the effect was different across the frailty spectrum (*p*-for-interaction=NR).

#### Multicomponent Intervention

We identified three trials that assessed the effects of multicomponent interventions among frailty subgroups.

##### Geriatric Assessment

In a trial of 541 patients with incurable cancer and impairment in one or more geriatric assessment domains,^39^ the effect of geriatric assessment compared with usual care on conversations (*p*-for-interaction = 0.611), concerns acknowledged (*p*-for-interaction = 0.740), and concerns addressed (*p*-for-interaction = 0.940) was beneficial, but the effect was similar across the frailty spectrum.

##### Pharmacist-Led Deprescribing Intervention

In a trial of 363 older adults living in the community conducted over 6 months in the US, the effect of pharmacist-led deprescribing intervention compared with usual care on changes in drug burden index 0.5 or more, anticholinergic cognitive burden was beneficial, but the effect was not different across the frailty spectrum (*p*-for-interaction=NR).^40, 41^

### Evaluation of Treatment Effect by Frailty Phenotype

#### Pharmacology Intervention

Four trials assessed whether the effect of the following treatments was different by frailty levels: antiplatelet medications,^42^ anticoagulants,^43^ osteoporosis medications,^16^ and androgen medications.^15^

##### Antiplatelet Medications

In a trial^42^ conducted across 52 countries with 9326 adults aged 65 or older suffering from unstable angina, prasugrel was associated with higher rates of the composite outcome of cardiovascular death, myocardial infarction, or stroke compared to clopidogrel in patients with one to two components of the frailty phenotype, but not in patients with zero or three to five components of frailty phenotype (*p*-for-interaction = 0.032).

##### Other

The remaining three interventions (including anticoagulants,^43^ osteoporosis medications,^16^ and androgen medications)^15^ were beneficial, but all concluded that intervention effect was not different across the frailty spectrum. Specifically, the effect of edoxaban, strontium, or testosterone gel compared with placebo on stroke or systemic embolism (*p*-for-interaction = 0.55), all-cause death (*p*-for-interaction = 0.06), net clinical composite outcome (*p*-for-interaction = 0.42), vertebral fracture (*p*-for-interaction = 0.11), and isometric knee extension peak torque (*p*-for-interaction = 0.68) was not different across the frailty spectrum.

#### Non-pharmacological Intervention

The included 10 trials assessed whether the effects of the following treatments were different by frailty levels: diabetes management^44^ and physical activity and exercise.^14, 37, 45, 46, 47, 49, 50, 51, 52^

##### Diabetes Management

In a trial conducted^44^ by Rodriguez-Manas in 2019 across 7 European countries, involving 964 adults aged over 70 with type 2 diabetes mellitus and functional impairment, multimodal intervention was compared with usual care over a 12-month follow-up. The results indicated diabetes management was beneficial, but no evidence of a differential effect of the multimodal intervention on Short Physical Performance Battery (SPPB) score across the frailty spectrum (*p*-for-interaction = 0.49).

##### Physical Activity and Exercise

There was significant effect of functional walking compared with usual pattern of activities on persistent mobility disability,^46^ physical rehabilitation intervention compared with attention control on SPPB score.^45,46^ The majority of the trials revealed no significant differences across the frailty spectrum concerning the effects of various physical interventions. These interventions, when compared to controls or usual care, consistently improved outcomes such as physical performance,^37, 47^ Mini-BESTest score,^14^ functional gait assessment,^14^ SPPB score,^47^ days at home,^49^ dominant handgrip strength,^50^ major mobility disability,^51^ persistent mobility disability^51^, skeletal muscle mass index,^52^ insulin-like growth factor,^52^ dehydroepiandrosterone sulfate,^52^ and 25-hydroxy vitamin D^52^ (*p*-for-interaction = NR or >0.05). Exceptions to this pattern were identified in two areas: (1) functional walking interventions were associated with higher risks of falls in patients with three to five components of the frailty phenotype compared to usual activities (*p*-for-interaction = 0.002);^46^ (2) physical rehabilitation intervention was associated with greater improvement in SPPB score in patients with three to five components of the frailty phenotype (*p*-for-interaction = 0.03).^37^

#### Multicomponent Intervention

We identified three trials that assessed the effects of multicomponent interventions among frailty subgroups.

##### Multifactorial, Interdisciplinary Intervention

Two articles^53, 54^ from the same RCT illustrated varying effects of multifactorial, interdisciplinary interventions (details shown in the footnote of Table 2) compared to usual care on patients with three to five components of the frailty phenotype, but not in patients with zero to two components of the frailty phenotype. A significant association between multidomain intervention and usual care was observed with greater Life Space Assessment score in patients with three components of the frailty phenotype at 3 months (*p*-for-interaction = 0.03), but not in patients with four to five components of the frailty phenotype and this effect attenuated by 12 months (*p*-for-interaction = 0.4). There was an increase in gait speed in patients with four to five components of the frailty phenotype at 12 months, but not in patients with three components of the frailty phenotype (*p*-for-interaction = 0.03). No significant differences were found in the effects of the intervention on SPPB and Physiological Profile Assessment across the frailty spectrum (*p*-for-interaction=NR).

##### Multidomain Intervention and Omega-3 Polyunsaturated Fatty Acids (n3 PUFA)

In the trial^20^ conducted by Tabue-teguo in 2018 in France, involving three different interventions (multicomponent + n3 PUFA vs multicomponent alone vs n3 PUFA alone, details shown in the footnote of Table 2) on a population of 1680 older adults over a 3-year follow-up, partial measures of cognitive function (Trail-Making Test A and B) showed significant improvement. However, no significant differences in cognitive function across the frailty spectrum were found (*p*-for-interaction > 0.05).

### Evaluation of Treatment Effect by Other Frailty Tools

Of the included trials, 20 trials measured frailty using other frailty assessment tools, for example, INTERMED for the Elderly Self-Assessment (INTERMED-E-SA),^55, 56^ Groningen Frailty Indicator (GFI),^36, 55–57^ frailty-associated conditions,^18^ modified frailty score,^19^ two tests of physical abilities,^58^ simplified Eastern Cooperate Oncology Group (ECOG)–based frailty assessment,^59, 60^ International Myeloma Working Group geriatric score,^17, 61, 62^ and the Study of Osteoporotic Fractures frailty index.^63^

#### Pharmacology Intervention

Seven trials assessed whether the effect of the following treatments were different by frailty levels: anti-neoplastic therapy^17, 59–62,64^ and vaccinations.^18^

##### Anti-neoplastic Therapy for Multiple Myeloma

Six trials assessed the effects of various treatments on progression-free survival (PFS) and overall survival (OS) in patients with different frailty levels. Four trials found that their interventions, including melphalan-prednisone-lenalidomide,^59^ ixazomib,^62^ and lenalidomide and dexamethasone^57^ were potentially associated with more prolonged PFS and OS in fit patients or patients with lower levels of frailty. Two trials found significant effect of lenalidomide compared to placebo,^17^ or daratumumab plus lenalidomide/dexamethasone compared with lenalidomide/dexamethasone^58^ on PFS^58^ or OS,^17^ but the effect was not different across the frailty spectrum (measured by International Myeloma Working Group geriatric score, simplified ECOG-based frailty assessment, or frailty assessment based on four components, separately) (*p*-for-interaction=NR in all trials).

##### Vaccinations

There was significant treatment effect of high-dose inactivated influenza vaccine compared with a standard-dose vaccine on laboratory-confirmed influenza, but the treatment effect was not different across the frailty spectrum measured by frailty-associated conditions (details shown in the footnotes of Table 3) (*p*-for-interaction = 0.838).^18^

#### Non-pharmacological Intervention

The included 11 trials assessed whether the effects of the following treatment were different by frailty levels: radiation therapy,^65^ surgical procedures,^66^ physical activity and exercise,^63, 67, 68^ psychosocial intervention,^69^ and others.^55, 56, 58, 70, 71^

##### Radiation Therapy

There was no significant treatment effect of 1-week course radiation therapy compared with a 3-week course on OS,^65^ and the effect was not different across the frailty spectrum measured by the Karnofsky Performance Status (*p*-for-interaction=NR).

##### Surgical Procedures

There was no significant treatment effect of anterior minimally invasive hemiarthroplasty compared with lateral Hardinge hemiarthroplasty on timed up and go duration,^66^ and the impact was not different across the frailty spectrum measured by the Functional Independence Measure, Charlson Index, and Medication score (*p*-for-interaction=NR).

##### Physical Activity and Exercise

A higher dose exercise program led to a greater improvement in health-related quality of life compared to no exercise in patients meeting at least two of three frailty assessments (including Combined Fried frailty phenotype, modified Physical Performance Test, and 7-point Clinical Frailty Scale) (*p*-for-interaction = 0.037).^67^ Additionally, physical activity was associated with higher total cardiovascular event rates compared to health education in patients with an SPPB score of 8 to 9, but not in those with an SPPB score <8 (*p*-for-interaction = 0.006).^68^ However, there was no evidence that the effect of physical activity compared with health education on 400-m gait-speed, 4-m gait-speed was different across the frailty spectrum (*p*-for-interaction=NR).^63^

##### Psychosocial Intervention

One psychosocial intervention was associated with improvement in instrumental activities of daily living (*p*-for-interaction < 0.01), potential enhancement in physical performance (*p*-for-interaction = 0.02), and a reduction in mortality (*p*-for-interaction = 0.01) in patients with a summary frailty index ≤3, but not in patients with a summary frailty index 4 to 5 (details shown in the footnotes of Table 3). However, these effects were not observed in patients with a frailty index of 4–5, indicating that the benefits of psychosocial intervention may be specific to lower levels of frailty.^69^

##### Others

No evidence that the effects of various interventions (for HF, stroke, physical frailty, or others), such as home telemedicine,^71^ home-based physiotherapy,^58^ and embrace program (details shown in the footnotes of Table 3),^55, 56^ differed across the frailty spectrum for non-fatal HF events, function measures, and various domains of health and well-being (*p*-for-interaction=NR or >0.05). However, a home-based intervention program was associated with a lower disability score compared to an educational program in patients meeting one of two frailty assessments (rapid gait test and chair stand test), but not in patients meeting both tests (*p*-for-interaction < 0.001 at 7 months and *p*-for-interaction = 0.005 at 12 months).^58^ Additionally, embrace was linked to greater improvements in quality of life in patients with INTERMED-E-SA ≥ 16, and patients with INTERMED-E-SA <16 and GFI 5 to 15, but not in patients with INTERMED-E-SA 16 to 20, or INTERMED-E-SA<16 and GFI <5 (*p*-for-interaction=NR).^55, 56^

#### Multicomponent Intervention

We identified two trials^19, 57^ that assessed the effect of multicomponent interventions among frailty subgroups.

##### Hospital-Based Disease Management Programs (DMP)

In one RCT^19^, 173 older patients with HF were randomly assigned to DMP or the usual care group. During a 2-year follow-up, a statistically significant effect of DMP compared to usual care was found in lowering all-cause admissions in patients with a higher modified frailty score, but not in patients with a lower frailty score (*p*-for-interaction = 0.018). However, the effect on death and/or HF admissions was not different across the frailty spectrum (*p*-for-interaction = 0.208).

##### Prevention of Care (POC) Approach

Metzelthin^57^ compared the POC approach with usual care in community dwelling frail older people and found no treatment effect on the Groningen Activity Restriction Scale, and the effect was not different across frailty subgroups measured by GFI (*p*-for-interaction > 0.05).

## DISCUSSION

We found several RCTs that examined whether the effects of pharmacological, non-pharmacological, and multicomponent interventions varied by frailty levels as measured using a deficit accumulation frailty index, frailty phenotype, or other frailty tools. For most pharmacological interventions including flu vaccines, the effect was consistent across frailty levels with a few exceptions: some interventions (e.g., edoxaban,^21^ prasugrel,^42^ chemotherapy for multiple myeloma^59, 61, 62^) were more effective or safer in robust patients, while the benefit of sacubitril/valsartan^28^ was greater in frail patients. Intensive lifestyle changes^11^ and exercise interventions^14, 35, 44, 45, 47, 49–52, 67^ seem to benefit frail older adults as much as or more than non-frail older adults. The effect of complex, multicomponent interventions depended on the type of the intervention. A multicomponent intervention delivered by an interdisciplinary team of physiotherapists, a dietician, a geriatrician, a rehabilitation physician, and a nurse resulted in a greater improvement in life space and gait speed among patients with frailty.^53, 54^ Similarly, a DMP for HF patients led to a greater reduction in all-cause admissions in frail patients.^19^ In contrast, frailty screening in routine primary care settings^36, 57^ or a multicomponent program including n3 PUFA supplementation in patients with memory complaints^20^ failed to demonstrate the benefit compared to usual care, regardless of frailty levels. Psychosocial support was more effective in functional recovery after stroke in less frail patients.^69^

The evaluation of treatment effect heterogeneity by patients’ frailty levels offers great potential to allow healthcare providers to optimize medical interventions in older adults. In scenarios where frailty significantly modifies treatment effects, healthcare providers can individualize the delivery of medical interventions that maximizes the benefit and minimizes the risks based on patients’ frailty levels. A common misconception in practice is that having frailty is conflated with decreased treatment efficacy, which may lead to under-treatment of frail patients as demonstrated by the delayed uptake of newly approved medications.^72–74^ By showing that treatment benefits do not vary by frailty status, under-treatment of frail patients can be avoided. Likewise, over-treatment can be minimized for frail patients near the end-of-life who may not gain benefit from treatments.

In applying the findings of our review to fully realize the potential of frailty-guided clinical management, there are important caveats to consider.^8^ First, the adoption of a standardized, validated frailty assessment tool is essential. Frailty assessment tools used in RCTs were often created post hoc using the items only available in the trial settings or were modified from the original tool, resulting in measurement error (i.e., misclassifying patients’ frailty levels). It also limits the applicability of the study findings. Adoption of a brief, standardized, validated frailty screening is imperative to translate the frailty-specific effects from RCTs into clinical practice. The Clinical Frailty Scale is a clinical judgment–based assessment that can be completed within 3 min and has been validated in various clinical settings, including primary care, emergency department, inpatient, and preoperative settings.^75, 76^ Second, post hoc analysis of RCTs should be considered hypothesis generating until further independent confirmation. Secondary analysis of RCTs that is not pre-specified is subject to confounding, type I error (false positive findings from multiple testing), and type II error (false negative findings due to inadequate power). Future adequately powered RCTs are needed to test for heterogeneity of treatment effects by the spectrum of frailty. Third, enrolling more older adults with frailty in RCTs is needed. Given the challenges in recruiting patients with frailty and assessing frailty in RCTs, pragmatic clinical trial or hybrid effectiveness and implementation study design in routine care settings should be considered. Lastly, the mechanisms by which frailty influences response to treatment are unknown. Further research is warranted to elucidate the biological mechanisms. Moreover, it remains uncertain whether improving frailty prior to treatment can change response to treatments by enhancing the benefits and mitigating the harms associated with treatments.

### Limitations

Our review has a few limitations. Despite our effort to identify RCTs that assessed the frailty subgroup-specific treatment effect in multiple electronic databases, we did not include publications in non-English literature and the studies that did not report frailty-specific effect estimates, raising the possibility of publication bias. The heterogeneity of frailty assessment tools, type of interventions, and outcomes precluded meta-analysis.

## CONCLUSION

This systematic review of post hoc analysis of RCTs suggests that the effectiveness and safety of certain pharmacological interventions can vary according to patients’ frailty levels and that the lifestyle or exercise interventions may benefit patients with frailty as much as or more than those without frailty. Although this preliminary evidence supports the potential utility of frailty assessment before treatment decisions, it also reveals the current challenges in delivering frailty-guided clinical care based on the post hoc analyses of RCTs. We call for further investigation into frailty-specific treatment effects by adopting a validated frailty assessment in RCTs of medical interventions involving older adults.

### Supplementary Information

Below is the link to the electronic supplementary material.

Supplementary file1 (DOCX 2283 KB)
