# Supplementary material for: Frailty as an Effect Modifier in Randomized Controlled Trials: A Systematic Review
Source: J Gen Intern Med. 2024 Apr 9;39(8):1452–73. doi: 10.1007/s11606-024-08732-8 (PMC11169165; doi:10.1007/s11606-024-08732-8)
Supplement: Supplementary file 1 — Supplementary file1 (DOCX 2283 KB) [file 11606_2024_8732_MOESM1_ESM.docx]

# Appendix Content

Frailty as An Effect Modifier in Randomized Controlled Trials: A Systematic Review

Aaron Yao, PhD; Linhui Gao, MS; Jiajun Zhang, MS; Joyce M. Cheng, BS; Dae Hyun Kim, Sc.D., M.D.

**Appendix 1.** Systematic database search strategies

**Appendix 2.** Risk of bias for included trials

**Appendix 3**. Forest plot of included trials

**Appendix 4**. Results from pharmacological, non-pharmacological, or multicomponent interventions

**Appendix 5.** Detailed summary of included trials

## Appendix 1. Systematic database search strategies

| Search ID | Strategy |
| --- | --- |
| **PubMed** | |
| #1 | "frail elderly"[MeSH Major Topic] |
| #2 | "frail elderly"[MeSH Terms] |
| #3 | "frail*"[Title/Abstract] |
| #4 | "prefrail*"[Title/Abstract] OR "pre frail*"[Title/Abstract] |
| #5 | #1 OR ... OR #4 |
| #6 | #5 Filters: Randomized Controlled Trial, English |
| **Web of Science** | |
| #1 | (TS=(frailty)) OR TS=(frail) |
| #2 | (((TS=(prefrail)) OR TS=(prefrailty)) OR TS=(pre-frail)) OR TS=(pre-frailty) |
| #3 | ((((TS=(older)) OR TS=(elder)) OR TS=(aged)) OR TS=(old age)) OR TS=(old aged) |
| #4 | ((TS=(randomized controlled trial)) OR TS=(randomized-controlled trial)) OR TS=(randomized-controlled trials) |
| #5 | #1 OR #2 |
| #6 | #3 AND #4 AND #5 AND English(Languages) |
| **Cochrane Library** | |
| #1 | MeSH descriptor: [Frailty] explode all trees |
| #2 | ("frailty"):ti,ab,kw OR ("frail"):ti,ab,kw OR ("pre-frail"):ti,ab,kw OR ("pre-frailty"):ti,ab,kw |
| #3 | ("prefrailty"):ti,ab,kw OR ("prefrail"):ti,ab,kw |
| #4 | ("older"):ti,ab,kw OR ("elder"):ti,ab,kw OR ("aged"):ti,ab,kw OR ("old age"):ti,ab,kw OR ("old aged"):ti,ab,kw |
| #5 | ("randomized controlled trial"):ti,ab,kw OR ("randomized-controlled trial"):ti,ab,kw OR ("randomized-controlled trials"):ti,ab,kw |
| #6 | #1 AND #2 AND #3 |
| #7 | #4 AND #5 AND #6 |
| **Embase** | |
| #1 | Frailty:ti,ab,kw |
| #2 | frail:ti,ab,kw |
| #3 | prefrail:ti,ab,kw |
| #4 | prefrailty:ti,ab,kw |
| #5 | 'older':ti,ab,kw OR 'elder':ti,ab,kw OR 'aged':ti,ab,kw OR 'old age':ti,ab,kw OR 'old aged':ti,ab,kw |
| #6 | 'randomized controlled trial':ti,ab,kw OR 'randomized-controlled trial':ti,ab,kw OR 'randomized-controlled trials':ti,ab,kw |
| #7 | #1 OR #2 OR #3 OR #4 |
| #8 | #5 AND #6 AND #7 |

## Appendix 2. Risk of bias for included trials *


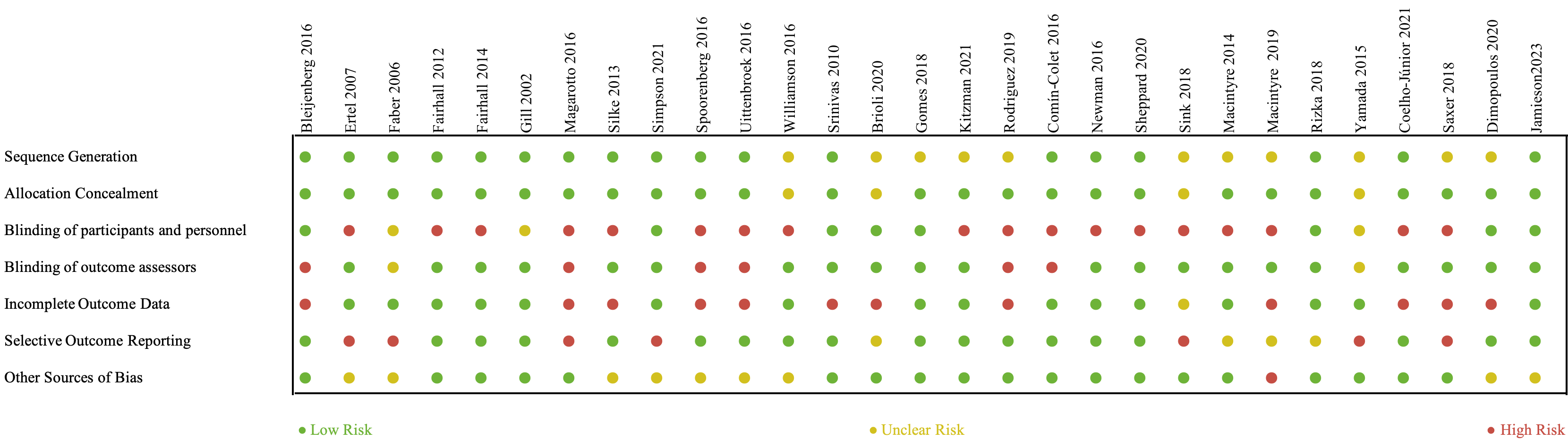


* Secondary analyses of RCT data, as presented in several articles, have not been evaluated for risk of bias.

## Appendix 3. Forest Plot of Included Trials*

*Forest plot showing the comparison of HR effect sizes and confidence intervals across multiple trials

Figure 1A. Forest Plot of Randomized Controlled Trials using Deficit Accumulation Frailty Index


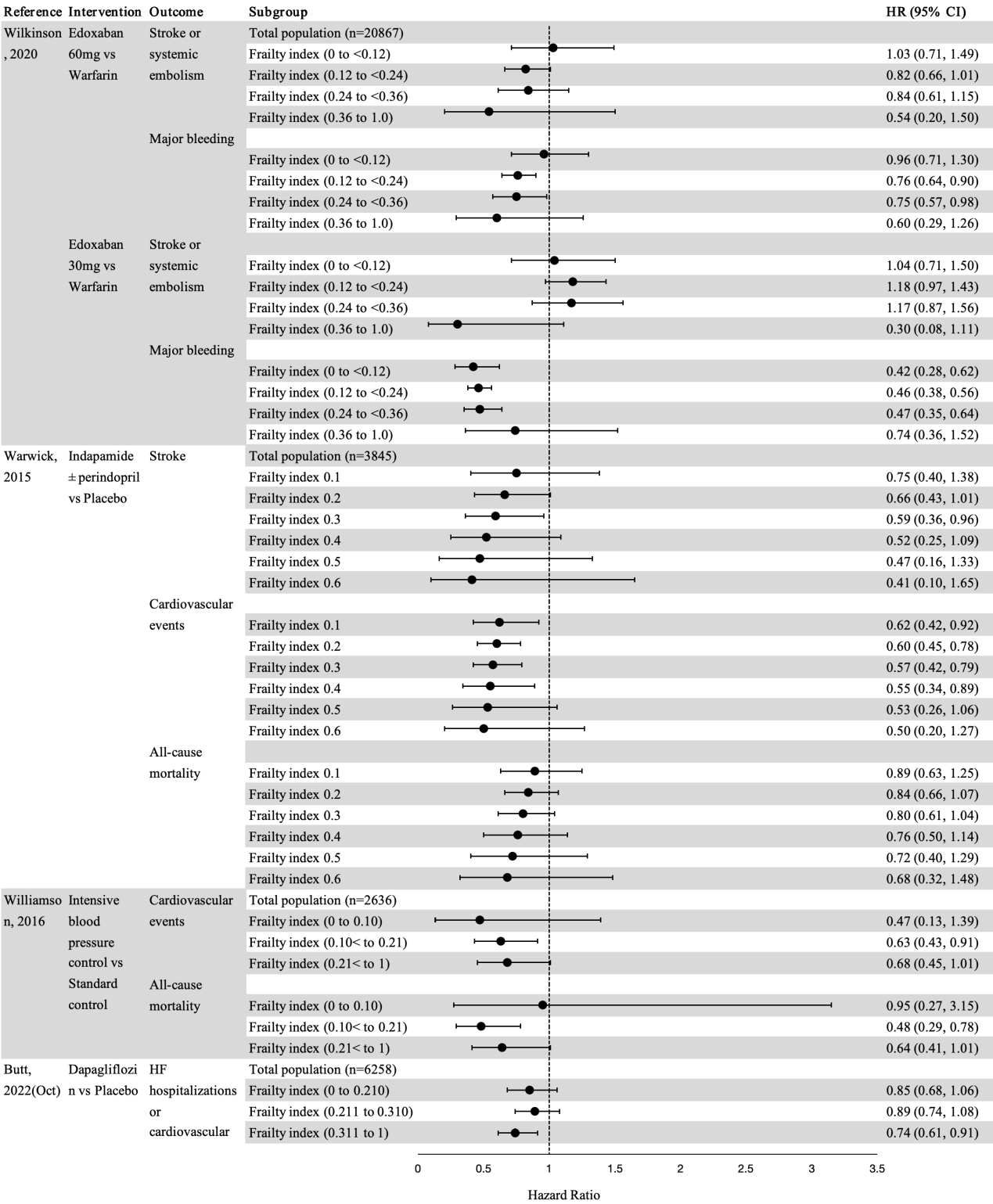


Figure 1B. Forest Plot of Randomized Controlled Trials using Deficit Accumulation Frailty Index


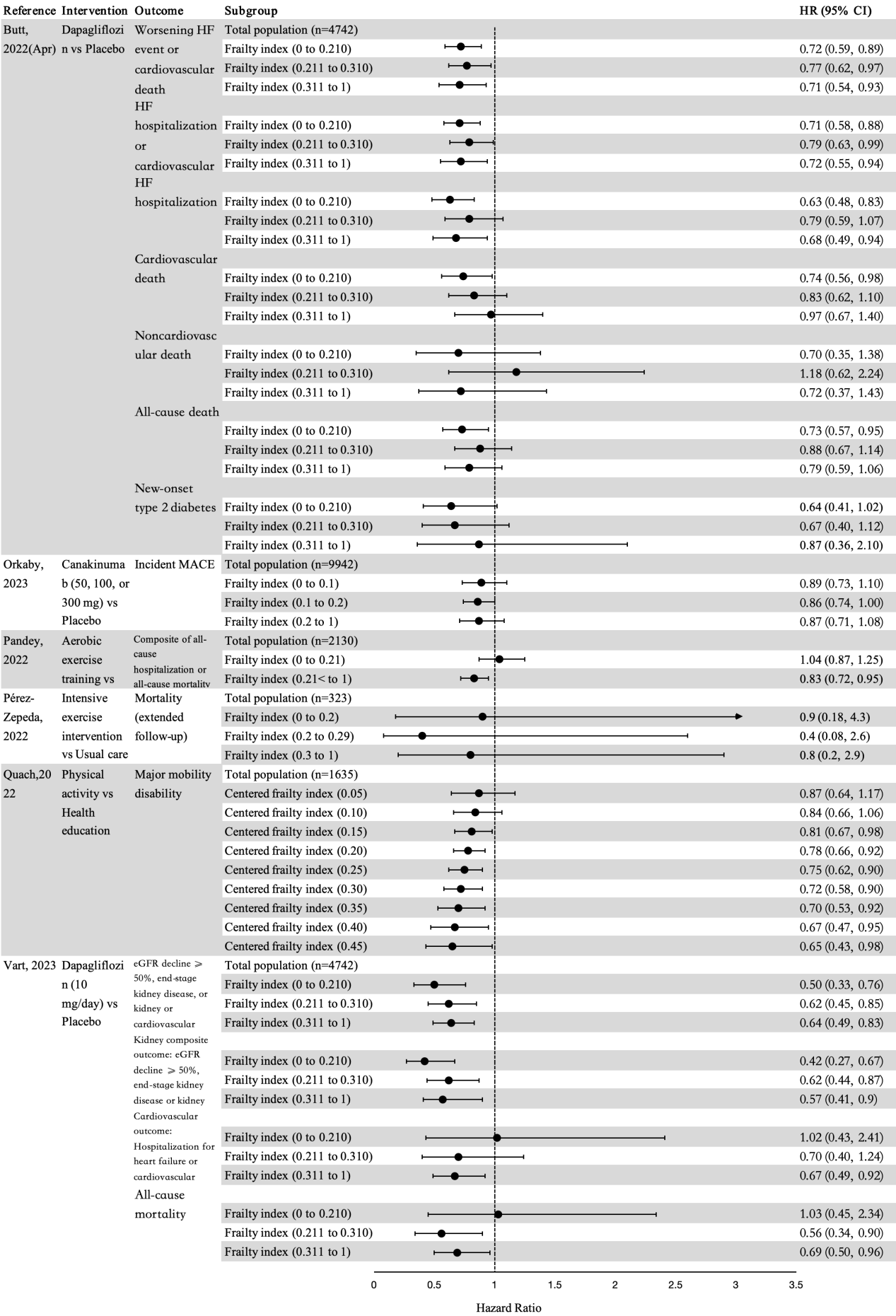


Figure 2. Forest Plot of Randomized Controlled Trials using Frailty Phenotype


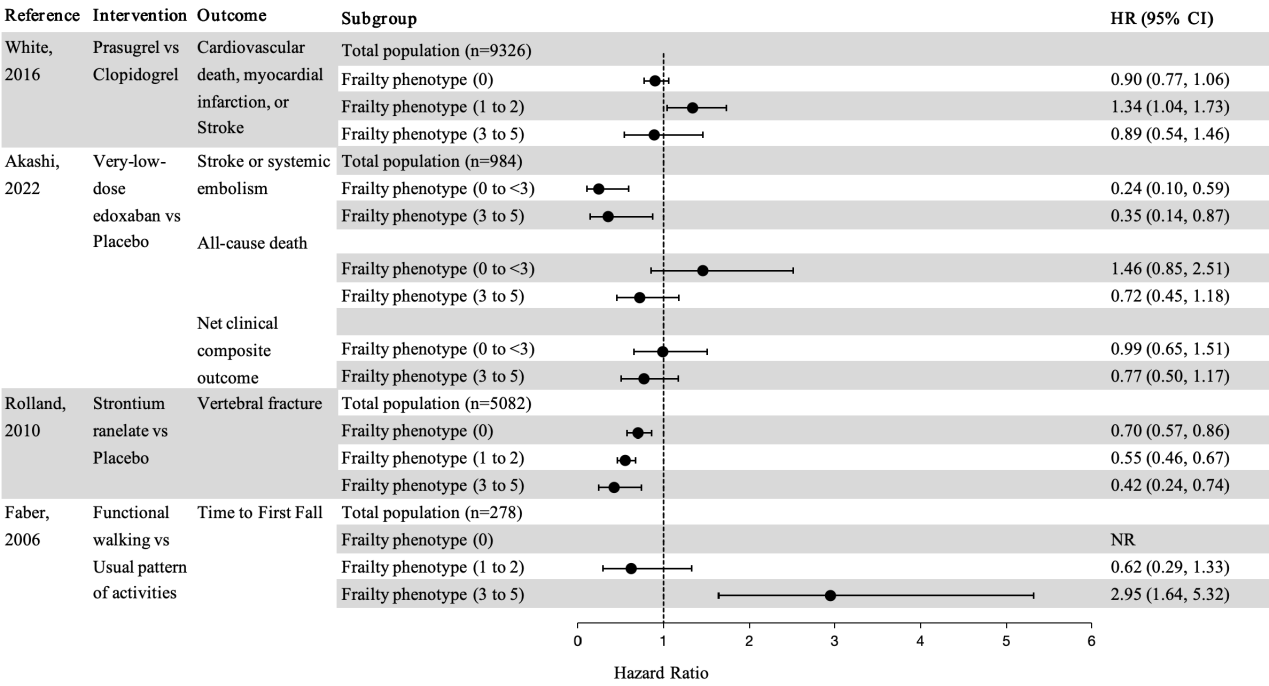


Figure 3. Forest Plot of Randomized Controlled Trials using Other Frailty Assessment Tools


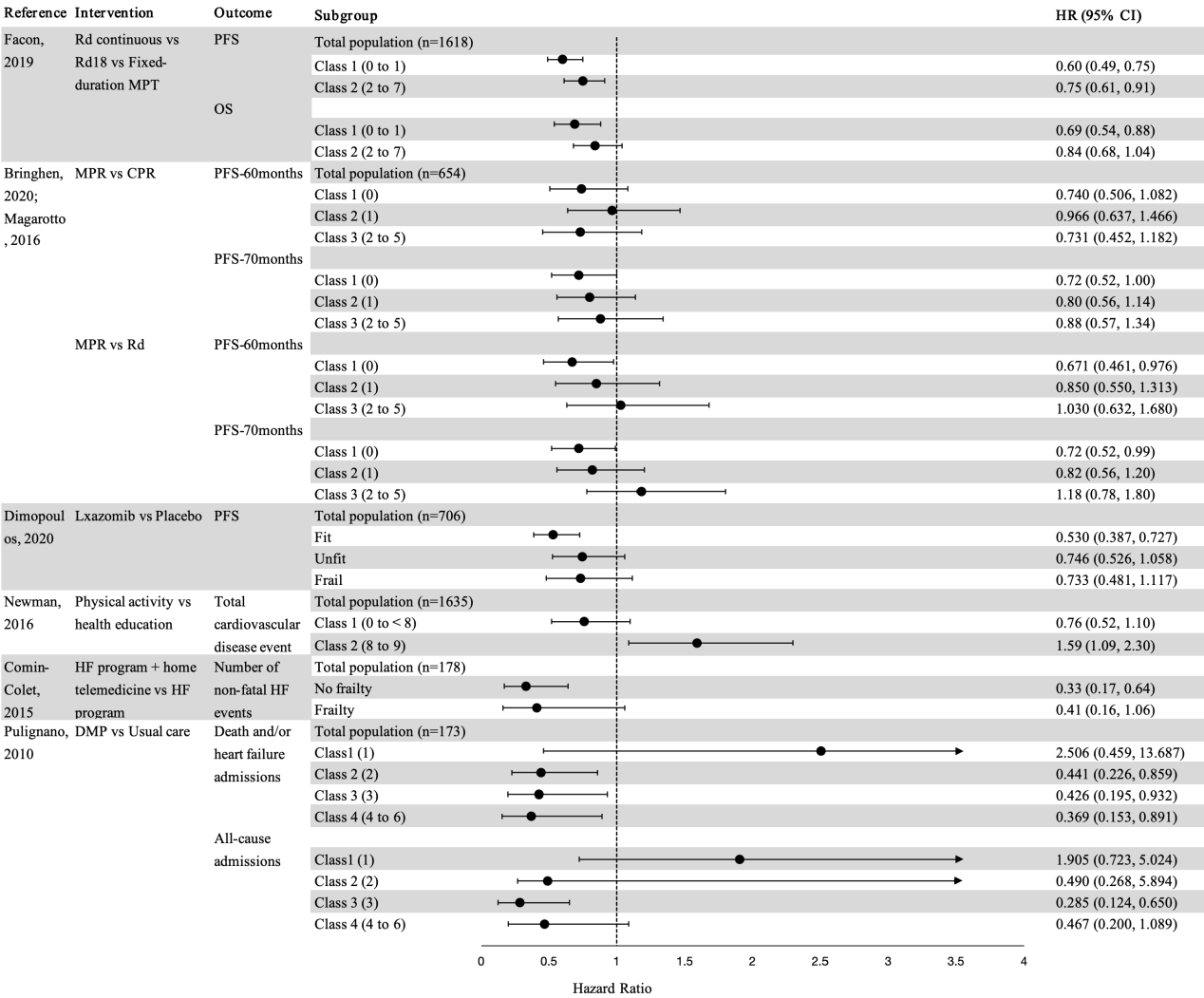


## Appendix 4. Results from pharmacological, non-pharmacological, or multicomponent interventions

### Table 1. Results from 26 pharmacological intervention

| **Intervention, Population,**  **Reference** | **Frailty assessment** | | **Frailty-Specific Results** | **Interpretation** |
| --- | --- | --- | --- | --- |
| **Pharmacological intervention - anticoagulants** | | | | |
| **Intervention:** Edoxaban 60 mg vs Edoxaban 30 mg vs Warfarin  **Population:** 20,867 adults with atrial fibrillation (86.8% were 60 years or older)  **Follow-up:** 34 months  **Reference:** Wilkinson, 2020, 46 countries^21^ | 40-item frailty index (range: 0-1)  A: Frailty index (0 to <0.12)  B: Frailty index (0.12 to <0.24)  C: Frailty index (0.24 to <0.36)  D: Frailty index (0.36 to 1.0) | | **Stroke or systemic embolism**  (edoxaban 60mg vs warfarin)  A: HR 1.03 (0.71, 1.49)  B: HR 0.82 (0.66, 1.01)  C: HR 0.84 (0.61, 1.15)  D: HR 0.54 (0.20, 1.50)  (edoxaban 30mg vs warfarin)  A: HR 1.04 (0.71, 1.50)  B: HR 1.18 (0.97, 1.43)  C: HR 1.17 (0.87, 1.56)  D: HR 0.30 (0.08, 1.11)  **Major bleeding**  (edoxaban 60mg vs warfarin)  A: HR 0.96 (0.71, 1.30)  B: HR 0.76 (0.64, 0.90)  C: HR 0.75 (0.57, 0.98)  D: HR 0.60 (0.29, 1.26)  (edoxaban 30mg vs warfarin)  A: HR 0.42 (0.28, 0.62)  B: HR 0.46 (0.38, 0.56)  C: HR 0.47 (0.35, 0.64)  D: HR 0.74 (0.36, 1.52) | There was no evidence that the effect of edoxaban compared with warfarin on stroke or systemic embolism was different across the frailty spectrum (p-for-interaction=NR).  Edoxaban was associated with lower rates of major bleeding compared with warfarin in patients with frailty index 0.12 to <0.36 (edoxaban 60mg) and patients with frailty index <0.36 (edoxaban 30mg) (p-for-interaction=NR). |
| **Intervention:** Very-low-dose edoxaban vs Placebo  **Population:** 984 patients with atrial fibrillation aged 80 years or older  **Follow-up:** 4 months  **Reference:** Akashi, 2022, Japan^41^ | Fried frailty criteria (range: 0-5)  A: Frailty phenotype (0 to <3)  B: Frailty phenotype (3 to 5) | | **Stroke or systemic embolism**  A: HR 0.24 (0.10, 0.59)  B: HR 0.35 (0.14, 0.87)  **All-cause death**  A: HR 1.46 (0.85, 2.51)  B: HR 0.72 (0.45, 1.18)  **Net clinical composite outcome**  A: HR 0.99 (0.65, 1.51)  B: HR 0.77 (0.50, 1.17) | There was no evidence that the effect of edoxaban compared with placebo on stroke or systemic embolism (p-for-interaction=0.55), all-cause death (p-for-interaction=0.06), and net clinical composite outcome was different across the frailty spectrum (p-for-interaction=0.42). |
| **Pharmacological intervention – antihypertensives** | | | | |
| **Intervention:** Antihypertensive medication reduction vs Usual care^*^  **Population:** 569 adults with hypertension aged 80 years and older  **Follow-up:** 12 weeks  **Reference:** Sheppard, 2020, UK^22^ | 36-item frailty index (range: 0-1)  A: Frailty index (0 to ≤0.12)  B: Frailty index (0.12< to 1) | | **Systolic blood pressure control**  A: RR 0.94 (0.85, ∞)  B: RR 1.01 (0.93, ∞)  **Change in systolic blood pressure**  A: MD 5.14 (1.40, 8.87) B: MD 2.26 (-0.90, 5.42) | There was no evidence that the effect of antihypertensive medication reduction compared with usual care on systolic blood pressure control (p-for-interaction=0.35) and change in systolic blood pressure (p-for-interaction=0.25) was different across the frailty spectrum. |
| **Intervention:** Intensive systolic blood pressure control vs Standard control  **Population:** 9361 adults aged 50 or older with hypertension  **Follow-up:** 39 months  **Reference:** Sink, 2018, US^23^ | 36-item frailty index (range 0-1)  A: Frailty index (0 to ≤0.21)  B: Frailty index (0.21< to 1) | | **Syncope:** NR  **Hypotension:** NR  **Falls:** NR | There was no evidence that the effect of intensive treatment compared with standard treatment on syncope (p-for-interaction>0.7), hypotension (p-for-interaction>0.7), and falls (p-for-interaction>0.7) was different across the frailty spectrum. |
| **Intervention:** Indapamide ± perindopril vs Placebo  **Population:** 3845 adults aged 80 and over with hypertension  **Follow-up:** 48 months  **Reference:** Warwick, 2015, Europe, China, Australia, Tunisia^24^ | 60-item frailty index (range: 0-1)  A: Frailty index 0.1  B: Frailty index 0.2 C: Frailty index 0.3  D: Frailty index 0.4  E: Frailty index 0.5 F: Frailty index 0.6 | | **Stroke**  A: HR 0.75 (0.40, 1.38)  B: HR 0.66 (0.43, 1.01) C: HR 0.59 (0.36, 0.96)  D: HR 0.52 (0.25, 1.09)  E: HR 0.47 (0.16, 1.33) F: HR 0.41 (0.10, 1.65)  **Cardiovascular events**  A: HR 0.62 (0.42, 0.92)  B: HR 0.60 (0.45, 0.78)  C: HR 0.57 (0.42, 0.79)  D: HR 0.55 (0.34, 0.89)  E: HR 0.53 (0.26, 1.06)  F: HR 0.50 (0.20, 1.27)  **All-cause mortality**  A: HR 0.89 (0.63, 1.25)  B: HR 0.84 (0.66, 1.07)  C: HR 0.80 (0.61, 1.04)  D: HR 0.76 (0.50, 1.14)  E: HR 0.72 (0.40, 1.29)  F: HR 0.68 (0.32, 1.48) | There was no evidence that the effect of indapamide ± perindopril compared with placebo on stroke (p-for-interaction=0.52), cardiovascular events (p-for-interaction=0.73), and all-cause mortality (p-for-interaction=0.61) was different across the frailty spectrum. |
| **Intervention:** Intensive blood pressure control vs Standard control    **Population:** 2636 persons aged 75 years or older with hypertension but without diabetes  **Follow-up:** 38 months  **Reference:** Williamson, 2016, US^25^ | 37-item frailty index (range: 0-1)  A: Frailty index (0 to 0.10) B: Frailty index (0.10< to 0.21) C: Frailty index (0.21< to 1) | | **Cardiovascular events**  A: HR 0.47 (0.13, 1.39)  B: HR 0.63 (0.43, 0.91)  C: HR 0.68 (0.45, 1.01)  **All-cause mortality**  A: HR 0.95 (0.27, 3.15)  B: HR 0.48 (0.29, 0.78)  C: HR 0.64 (0.41, 1.01) | There was no evidence that the effect of intensive treatment compared with standard treatment on cardiovascular events (p-for-interaction=0.84) and all-cause mortality (p-for-interaction=0.52) was different across the frailty spectrum. |
| **Intervention:** Intensive blood pressure target vs Standard blood pressure target  **Population:** 547 adults with hypertension and increased cardiovascular risk but free of diabetes or dementia  **Follow-up:** 4 years  **Reference:** Dolui, 2022, ^26^US | 36-item frailty index (range: 0-1)  A: Frailty index (0 to ≤0.10)  B: Frailty index (0.10< to ≤0.21)  C: Frailty index (0.21< to 1) | | **Whole Brain Cerebral Blood Flow**  A: difference in change 4.50 (0.02, 8.99)  B: difference in change 3.18 (0.49, 5.86)  C: difference in change -1.11 (-5.13, 2.90) | There was no evidence that the effect of intensive treatment compared with standard treatment on whole brain cerebral blood flow (p-for-interaction=0.13) was different across the frailty spectrum. |
| **Pharmacological intervention – heart failure medications** | | | | |
| **Intervention:** Sacubitril/valsartan vs Valsartan  **Population:** 4795 patients with HFpEF  **Follow-up:** 48 months  **Reference:** Butt, 2022(Sep), UK^28^ | 41-item frailty index (range: 0-1)  A: Frailty index (0 to 0.210)  B: Frailty index (0.211 to 0.310)  C: Frailty index (0.311 to 1) | | **HF hospitalizations or cardiovascular death**  A: RR 0.98 (0.76, 1.27)  B: RR 0.92 (0.76, 1.12)  C: RR 0.69 (0.51, 0.95)  **HF hospitalizations**  A: RR 0.95 (0.71, 1.29)  B: RR 0.93 (0.75, 1.16)  C: RR 0.64 (0.45, 0.91) | Sacubitril/valsartan was associated with lower rates of heart failure hospitalization or cardiovascular death (p-for-interaction=0.002) and heart failure hospitalization (p-for-interaction=0.001) compared with valsartan in patients with frailty index 0.311 to 1, but not in patients with frailty index <0.311. |
| **Intervention:** Spironolactone 15–45 mg daily vs Placebo  **Population:** 1767 adults (mean age=71.5 years) with HFpEF  **Follow-up:** 40 months  **Reference:** Sanders, 2018, US, Canada, Brazil, Argentina^27^ | 39-item frailty index (range: 0-1)^†^  A: Frailty index (0 to <0.3)  B: Frailty index (0.3 to <0.4)  C: Frailty index (0.4 to <0.5)  D: Frailty index (0.5 to 1) | | **HF hospitalization or cardiovascular death**^†^  A: HR 0.78 (0.52, 1.16)  B: HR 0.79 (0.58, 1.06)  C: HR 1.00 (0.73, 1.39)  D: HR 0.74 (0.51, 1.07) | There was no evidence that the effect of spironolactone compared with placebo on heart failure hospitalization or cardiovascular death was different across the frailty spectrum (p-for-interaction=0.55). |
| **Pharmacological intervention - inhibitors** | | | | |
| **Intervention:** Dapagliflozin vs Placebo  **Population:** 4742 patients with symptomatic heart failure with a left ventricular ejection fraction of 40% or less and elevated natriuretic peptide  **Follow-up:** 18.2 (median) months  **Reference:** Butt, 2022(Apr), 20 countries^29^ | 32-item frailty index (range: 0-1)  A: Frailty index (0 to 0.210)  B: Frailty index (0.211 to 0.310)  C: Frailty index (0.311 to 1) | | **Refer to Appendix Table** | Dapagliflozin was associated with lower rates of cardiovascular death (p-for-interaction=NR) and all-cause death (p-for-interaction=NR) compared with placebo in patients with frailty index ≤0.210.  Dapagliflozin was associated with lower rates of HF hospitalization (p-for-interaction=NR) compared with placebo in patients with frailty index ≤0.210 or frailty index ≥0.311.  There was no evidence that the effect of dapagliflozin compared with placebo on worsening HF event or cardiovascular death, HF hospitalization or cardiovascular death, noncardiovascular death, new-onset type 2 diabetes was different across the frailty spectrum (p-for-interaction=NR). |
| **Intervention:** Dapagliflozin vs Placebo  **Population:** 6258 patients with HF and mildly reduced or preserved left ventricular ejection fraction  **Follow-up:** 36 months  **Reference:** Butt, 2022(Oct), UK^30^ | 41-item frailty index (range: 0-1)  A: Frailty index (0 to 0.210)  B: Frailty index (0.211 to 0.310)  C: Frailty index (0.311 to 1) | | **HF hospitalizations or cardiovascular death**  A: HR 0.85 (0.68, 1.06)  B: HR 0.89 (0.74, 1.08)  C: HR 0.74 (0.61, 0.91) | There was no evidence that the effect of dapagliflozin compared with placebo on heart failure hospitalization or cardiovascular death was different across the frailty spectrum (p-for-interaction=0.40). |
| **Intervention:** Dapagliflozin (10 mg/day) vs Placebo  **Population:** 4303 adults with CKD, with/without type 2 diabetes, with an estimated glomerular filtration rate of 25–75 mL/min/1.73 m2, and urinary albumin-to-creatinine ratio 200–5 000 mg/g  **Follow-up:** 2 (median) years  **Reference:** Vart, 2023, 21 countries^31^ | 32-item frailty index (range: 0-1)  A: Frailty index (0 to 0.210)  B: Frailty index (0.211 to 0.310)  C: Frailty index (0.311 to 1) | | **eGFR decline ≥ 50%, end-stage kidney disease, or kidney or cardiovascular death**  A: HR 0.50 (0.33, 0.76)  B: HR 0.62 (0.45, 0.85)  C: HR 0.64 (0.49, 0.83)  **Kidney composite outcome: eGFR decline ≥ 50%, end-stage kidney disease or kidney death**  A: HR 0.42 (0.27, 0.67)  B: HR 0.62 (0.44, 0.87)  C: HR 0.57 (0.41, 0.9)  **Cardiovascular outcome: Hospitalization for heart failure or cardiovascular death**  A: HR 1.02 (0.43, 2.41)  B: HR 0.70 (0.40, 1.24)  C: HR 0.67 (0.49, 0.92)  **All-cause mortality**  A: HR 1.03 (0.45, 2.34)  B: HR 0.56 (0.34, 0.90)  C: HR 0.69 (0.50, 0.96) | There was no evidence that the effect of dapagliflozin compared with placebo on eGFR decline ≥ 50%, end-stage kidney disease, or kidney or cardiovascular death (p-for-interaction=0.667), kidney composite outcome (p-for-interaction=0.437), cardiovascular outcome (p-for-interaction=0.627), all-cause mortality (p-for-interaction=0.417) was different across the frailty spectrum. |
| **Intervention:** Canakinumab (50, 100, or 300 mg) vs  Placebo  **Population:** 9942 patients with stable post-myocardial infarction  **Follow-up:** 60 months  **Reference:** Orkaby, 2023, 39 countries^13^ | 34-item frailty index (range: 0-1)  A: Frailty index (0 to <0.1)  B: Frailty index (0.1≤ to <0.2)  C: Frailty index (0.2≤ to 1) | | **Incident MACE**  A: HR 0.89 (0.73, 1.10)  B: HR 0.86 (0.74, 1.00)  C: HR 0.87 (0.71, 1.08) | There was no evidence that the effect of canakinumab compared with placebo on incident MACE was different across the frailty spectrum (p-for-interaction=NR). |
| **Pharmacological intervention - vaccinations** | | | | |
| **Intervention:** Adjuvanted recombinant zoster vaccine vs Placebo  **Population:** 29305 adults aged 50 years and older  **Follow-up:** 36 months  **Reference:** Curran, 2020, 17 countries^32^ | 41-item frailty index^*^ (range: 0-1)  A: Frailty index (0 to 0.08)  B: Frailty index (0.08< to 0.25)  C: Frailty index (0.25< to 1.0) | | **Vaccine efficacy**^†^  A: 95.8%  B: 90.4%  C: 90.2% | There was no evidence that the effect of recombinant zoster vaccine compared with placebo on herpes zoster was different across the frailty spectrum (p-for-interaction=NR). |
| **Intervention:** 23-valent polysaccharide vaccine vs 23-valent polysaccharide vaccine with 7-valent pneumococcal conjugate vaccine  **Population:** 312 frail hospitalized adults aged 60 years and older  **Follow-up:** 12 months  **Reference:** Macintyre 2014, Australia^33^  Macintyre 2019, Australia^34^ | 40-item frailty index (range: 0-1)  A: Frailty index (0 to 0.250)  B: Frailty index (0.250< to 0.375)  C: Frailty index (0.375< to 1) | | **Serotype 4 IgG (mg/mL)**  (12 months^†^)  A: 1.0 vs 1.5  B/C: 0.8 vs 0.6  (72 months)  A: 1.6 vs 1.7  B/C: 1.2 vs 0.6  **Serotype 18C IgG (mg/mL)**  (12 months^†^)  A: 5.0 vs 5.4  B/C: 2.9 vs 2.7  (72 months)  A: 6.6 vs 5.9  B/C: 5.5 vs 2.3  **Serotype 19F IgG (mg/mL)**  (12 months^†^)  A: 2.8 vs 3.9  B/C: 2.3 vs 1.8 | There was no evidence that the response of 23-valent polysaccharide vaccine with 7-valent pneumococcal conjugate vaccine compared with 23-valent polysaccharide vaccine was different across the frailty spectrum (p-for-interaction=NR). |
| **Intervention:** High-dose inactivated influenza vaccine vs Standard-dose vaccine  **Population:** 31,989 medically stable adults  **Follow-up:** 6-8 months  **Reference:** DiazGranados, 2015, US and Canada^18^ | Frailty-associated conditions  (0-14 conditions^b^)  A: Class 1 (0)  B: Class 2 (1)  C: Class 3 (2)  D: Class 4 (3 to 14) | | **Laboratory-confirmed influenza caused by any viral type/subtype (regardless of similarity to the vaccine)**  A: 34.0 (-7.9, 60.2)  B: 27.5 (0.4, 47.4)  C: 23.9 (-9.0, 47.2)  D: 16.0 (-16.3, 39.4) | There was no evidence that the effect of high-dose inactivated influenza vaccine compared with standard-dose vaccine on laboratory-confirmed influenza caused by any viral type/subtype (regardless of similarity to the vaccine) was different across the frailty spectrum (p-for-interaction=0.838). |
| **Pharmacological intervention – antiplatelet medications** | | | | |
| **Intervention:** Prasugrel vs Clopidogrel  **Population:** 9326 adults aged 65 years or older with unstable angina  **Follow-up:** 3 months  **Reference:** White, 2016, 52 countries^40^ | Fried frailty criteria (range: 0-5)  A: Frailty phenotype (0)  B: Frailty phenotype (1 to 2)  C: Frailty phenotype (3 to 5) | **Cardiovascular death, myocardial infarction, or Stroke**  A: HR 0.90 (0.77, 1.06)  B: HR 1.34 (1.04, 1.73)  C: HR 0.89 (0.54, 1.46) | | Prasugrel was associated with higher rates of composite of cardiovascular death, myocardial infarction, or stroke compared with clopidogrel in patients with 1-2 components of the frailty phenotype (p-for-interaction=0.032). |
| **Pharmacological intervention - osteoporosis medications** | | | | |
| **Intervention:** Strontium ranelate vs Placebo  **Population:** 5,082 older women with osteoporotic  **Follow-up:** 3 years  **Reference:** Rolland, 2010, 12 countries^16^ | Fried frailty criteria (range: 0-5)  A: Frailty phenotype (0)  B: Frailty phenotype (1 to 2)  C: Frailty phenotype (3 to 5) | **Vertebral fracture**  A: HR 0.70 (0.57, 0.86)  B: HR 0.55 (0.46, 0.67)  C: HR 0.42 (0.24, 0.74) | | There was no evidence that the effect of strontium ranelate compared with placebo on vertebral fracture was different across the frailty spectrum (p-for-interaction=0.11). |
| **Pharmacological intervention - androgen medications** | | | | |
| **Intervention:** Testosterone gel vs Placebo  **Population:** 262 men aged 65 years or older  **Follow-up:** 6 months  **Reference:** Srinivas-Shankar, 2010, UK^15^ | Fried frailty criteria (range: 0-5)  A: Frailty phenotype (1)  B: Frailty phenotype (2)  C: Frailty phenotype (3)  D: Frailty phenotype (4) | **IME-PT**  Adjusted difference:  9.48 (-4.05, 23.02) | | There was no evidence that testosterone gel compared with placebo on IME-PT was different across the frailty spectrum (p-for-interaction=0.68). |
| **Pharmacological intervention - myeloma medications** | | | | |
| **Intervention:** Lenalidomide vs Placebo  **Population:** 40 adults with myeloma  **Follow-up:** 12.9 months (median)  **Reference:** Brioli, 2019, Germany^17^ | International Myeloma Working Group geriatric score (range: 0-5)  A: Class 1 (0)  B: Class 2 (1)  C: Class 3 (2 to 5) | **PFS**: NR  **OS**: NR | | There was no evidence that the effect of lenalidomide compared with placebo on PFS and OS was different across the frailty spectrum (p-for-interaction=NR). |
| **Intervention:** Rd continuous vs Rd18 vs Fixed-duration MPT  **Population:** 1618 transplant-ineligible adults with newly diagnosed multiple myeloma  **Follow-up:** 90 months  **Reference:** Facon, 2019, Europe, North America, and the Asia–Pacific region^57^ | Simplified ECOG-based frailty assessment (range: 0-7)  A: Class 1 (0 to 1)  B: Class 2 (2 to 7) | **PFS**  A: HR 0.60 (0.49, 0.75)  B: HR 0.75 (0.61, 0.91)  **OS**  A: HR 0.69 (0.54, 0.88)  B: HR 0.84 (0.68, 1.04) | | Rd continuous was potentially associated with more prolonged PFS and OS compared with MPT in patients with Simplified ECOG-based frailty assessment <1 (p-for-interaction=NR). |
| **Intervention:** Daratumumab plus lenalidomide/dexamethasone vs Lenalidomide/dexamethasone  **Population:** 737 patients with transplant-ineligible newly diagnosed multiple myeloma  **Follow-up:** 36.4 (median) months  **Reference:** Facon, 2022, North America, Europe, the Middle East, and the Asia–Pacific region^58^ | Simplified ECOG-based frailty assessment (range: 0-7)  A: Class 1 (0 to 1)  B: Class 2 (2 to 7) | **Progression-free survival**  A: HR 0.48  B: HR 0.003 | | There was no evidence that the effect of daratumumab plus lenalidomide/dexamethasone compared with lenalidomide/dexamethasone on progression-free survival was different across the frailty spectrum (p-for-interaction=NR). |
| **Intervention:** MPR vs CPR vs Rd (low-dose)  **Population:** 654 adults aged 50-89 years with multiple myeloma  **Follow-up:** 60months, 70 months  **Reference:** Bringhen, 2020, Italian and Czech Republic^59^  Magarotto, 2016, Italy and Czech Republic^60^ | International Myeloma Working Group geriatric score (range: 0-5)  A: Class 1 (0)  B: Class 2 (1)  C: Class 3 (2 to 5) | **PFS**  (MPR vs CPR)  60 months  A: HR 0.740 (0.506, 1.082)  B: HR 0.966 (0.637, 1.466)  C: HR 0.731 (0.452, 1.182)  70 months  A: HR 0.72 (0.52, 1.00)  B: HR 0.80 (0.56, 1.14)  C: HR 0.88 (0.57, 1.34)  (MPR vs Rd)  60months  A: HR 0.671 (0.461, 0.976)  B: HR 0.850 (0.550, 1.313)  C: HR 1.030 (0.632, 1.680)  70months  A: HR 0.72 (0.52, 0.99)  B: HR 0.82 (0.56, 1.20)  C: HR 1.18 (0.78, 1.80) | | MPR was potentially associated with more prolonged PFS compared with Rd in fit patients (p-for-interaction=NR). |
| **Intervention:** Lxazomib vs Placebo  **Population:** 706 adults aged 42-90 years with multiple myeloma  **Follow-up:** NR  **Reference:** Dimopoulos, 2020, 34 countries^62^ | Frailty assessment based on four components^a^  A: Fit / B: Unfit / C: Frail (NR) | **PFS**  A: HR 0.530 (0.387, 0.727)  B: HR 0.746 (0.526, 1.058)  C: HR 0.733 (0.481, 1.117) | | Lxazomib was potentially associated with more prolonged PFS compared with placebo in fit patients, but not in unfit and frail patients (p-for-interaction=NR). |

### Table 2. Results from 27 non-pharmacological intervention

| **Intervention, Population,**  **Reference** | **Frailty assessment** | **Frailty-Specific Results** | **Interpretation** |  |
| --- | --- | --- | --- | --- |
| **Non-pharmacological intervention – diabetes management** | | | |  |
| **Intervention:** Intensive lifestyle intervention vs Diabetes support and education  **Population:** 5145 adults aged 45-76 years with type 2 diabetes and overweight or obesity  **Follow-up:** 118 months  **Reference:** Simpson, 2021, US^11^ | 38-item frailty index (range: 0-1)  A: Frailty index (0 to <0.178)  B: Frailty index (0.178≤ to <0.230)  C: Frailty index (0.230≤ to 1) | **Cardiovascular events:**  A: RR 0.73 (0.55, 0.98)  B: RR 0.97 (0.72, 1.17) C: RR 1.15 (0.94, 1.42) | Intensive lifestyle intervention was associated with lower cardiovascular events compared with diabetes support and education in patients with frailty index <0.178, not in patients with frailty index ≥0.178 (p-for-interaction=0.01). |  |
| **Intervention:** Multimodal intervention^a^ vs Usual care  **Population:** 964 adults aged over 70 years older adults with type 2 diabetes mellitus and functional impairment  **Follow-up:** 12 months  **Reference:** Rodriguez-Manas, 2019, 7 European countries^42^ | Fried frailty criteria (range: 0-5)  A: Frailty phenotype (1 to 2)  B: Frailty phenotype (3 to 5) | **Changes in physical function**: NR | There was no evidence that the effect of multimodal intervention compared with usual care on physical function was different across the frailty spectrum (p-for-interaction=0.49). | |
| **Non-pharmacological intervention – physical activity and exercise** | | | |  |
| **Intervention:** Chair yoga vs Health education  **Population:** 112 adults aged 65 years or older with lower extremity osteoarthritis  **Follow-up:** 8 weeks  **Reference:** Park, 2020, US^35^ | 82-item frailty index (range: 0-1)  A: Quartile 1 (mean frailty index 0.39)  B: Quartile 2 (mean frailty index 0.43)  C: Quartile 3 (mean frailty index 0.47)  D: Quartile 4 (mean frailty index 0.57) | **WOMAC pain score:** NR  **Pain interference:** NR | Chair yoga was associated with lower WOMAC pain score (p-for-interaction=0.02) and pain interference (p-for-interaction=0.01) compared with health education in patients with frailty index quartile 3 and 4, not in patients with frailty index quartile 1 and 2. |  |
| **Intervention:** Aerobic exercise training vs Usual care  **Population:** 2130 stable patients with HFrEF  **Follow-up:** 36 months  **Reference:** Pandey, 2022, United States, Canada, and France^37^ | 36-item frailty index (range: 0-1)  A: Frailty index (0 to ≤ 0.21)  B: Frailty index (0.21< to 1) | **Composite of all-cause hospitalization or all-cause mortality**  A: HR 1.04 (0.87, 1.25)  B: HR 0.83 (0.72, 0.95) | Aerobic exercise training was associated with lower rates of composite of all-cause hospitalization or all-cause mortality compared with usual care in patients with frailty index >0.21 (p-for-interaction=NR). |  |
| **Intervention:** Intensive exercise intervention vs Usual care  **Population:** 323 adults admitted to an acute care ward  **Follow-up:** 58 months  **Reference:** Pérez-Zepeda, 2022, Spain^12^ | 63-item frailty index (range: 0-1)  A: Frailty index (0 to <0.2)  B: Frailty index (0.2≤ to < 0.29)  C: Frailty index (0.3≤ to 1) | **Mortality**  A: HR 0.9 (0.18, 4.3)  B: HR 0.4 (0.08, 2.6)  C: HR 0.8 (0.2, 2.9)  **Refer to Appendix Table** | There was no evidence that the effect of intensive exercise compared with usual care on mortality was different across the frailty spectrum (p-for-interaction=NR). |  |
| **Intervention:** Physical activity vs Health education  **Population:** 1635 community-dwelling participants  **Follow-up:** 2 years  **Reference:** Quach, 2022, US^38^ | 44-item frailty index  A: Frailty index centered in 0.05  B: Frailty index centered in 0.10  C: Frailty index centered in 0.15  D: Frailty index centered in 0.20  E: Frailty index centered in 0.25  F: Frailty index centered in 0.30  G: Frailty index centered in 0.35  H: Frailty index centered in 0.40  I: Frailty index centered in 0.45 | **Major mobility disability**  A: HR 0.87 (0.64, 1.17)  B: HR 0.84 (0.66, 1.06)  C: HR 0.81 (0.67, 0.98)  D: HR 0.78 (0.66, 0.92)  E: HR 0.75 (0.62, 0.90)  F: HR 0.72 (0.58, 0.90)  G: HR 0.70 (0.53, 0.92)  H: HR 0.67 (0.47, 0.95)  I: HR 0.65 (0.43, 0.98) | Physical activity was associated with lower rates of major mobility disability compared with health education in patients with centered frailty index ≥0.15 (p-for-interaction=NR). |  |
| **Intervention:** Individual training (Nintendo Wii Fit Plus) vs Physical activity education  **Population:** 30 older adults aged 71-92 years  **Follow-up:** 30 days  **Reference:** Gomes, 2018, Brazil^14^ | Fried frailty criteria (range: 0-5)  A: Frailty phenotype: 1-2  B: Frailty phenotype: >=3 | **Mini-BESTest score at final and follow-up assessments** ^†^  (Final)  A: mean 17.6 (14.8, 20.5) vs mean 19.1 (17.4, 20.9)  B: mean 19.6 (16.6, 22.6) vs mean 15.8 (13.6, 18.0)  (Follow-up)  A: mean 16.9 (14.1, 19.7) vs mean 19.0 (17.2, 20.8)  B: mean 17.6 (14.3, 20.9) vs mean 15.7 (13.0, 18.3)  **FGA at final and follow-up assessments ^†^**  (Final)  A: mean 19.5 (17.2, 21.9) vs mean 18.2 (16.2, 20.3)  B: mean 18.0 (16.2, 19.8) vs mean 15.8 (13.5, 18.0)  (Follow-up)  A: mean 19.9 (17.4, 22.4) vs mean 19.8 (17.8, 21.7)  B: mean 19.0 (17.1, 20.9) vs mean 17.6 (15.4, 19.9) | There was no evidence that the effect of individual training (Nintendo Wii Fit Plus) compared with physical activity education on Mini-BESTest score (p-for-interaction>0.05), and FGA (p-for-interaction>0.05) was different across the frailty spectrum. |  |
| **Intervention:** LSRT vs HSRT vs control  **Population:** 60 adults aged 60 years and older  **Follow-up:** NR  **Reference:** Coelho-junior, 2021, Brazil^43^ | Fried frailty criteria (range: 0-5)  A: Frailty phenotype (1 to 2)  B: Frailty phenotype (3 to 5) | Refer to Appendix Table | There was no evidence that the effect of LSRT and HSRT compared with control on physical performance was different across the frailty spectrum (p-for-interaction=NR). |  |
| **Interaction:** Functional walking vs Usual pattern of activities  **Population:** 278 adults aged 63-98 years  **Follow-up:** 52 weeks  **Reference:** Faber, 2006, Netherland^44^ | Fried frailty criteria (range: 0-5)  A: Frailty phenotype (0)  B: Frailty phenotype (1 to 2)  C: Frailty phenotype (3 to 5) | **POMA**  B: mean 1.1 (0.4, 1.8)  C: mean 0.5 (-0.6, 1.7)  **PPS**  B: mean 0.7 (0.3, 1.2) C: mean -0.7 (-1.3, -0.0)  **Time to First Fall**  B: HR 0.62 (0.29, 1.33)  C: HR 2.95 (1.64, 5.32) | There was no evidence that the effect of functional walking compared with usual pattern of activities on PPS was different across the frailty spectrum (p-for-interaction=NR).  Functional walking was associated with higher risks of fall compared with usual pattern of activities in patients with three or more frailty phenotypes, but not in patients with 1-2 components of the frailty phenotype (p-for-interaction=0.002). |  |
| **Intervention:** Tailored, progressive physical rehabilitation vs Usual care  **Population:** 349 adults aged 60 years or older with heart failure  **Follow-up:** 3 months  **Reference:** Kitzman, 2021,  US^45^ | Modified Fried frailty criteria (range: 0-5)  A: Frailty phenotype (0)  B: Frailty phenotype (1 to 2)  C: Frailty phenotype (3 to 5) | **SPPB score**  (Between-Group Difference in Mean Score, 95% CI)  A or B: 0.7 (-0.1, 1.5)  C: 2.1 (1.3, 2.8) | There was no evidence that the effect of tailored, progressive physical rehabilitation compared with usual care on SPPB score was different across the frailty spectrum (p-for-interaction=NR). |  |
| **Intervention:** Physical rehabilitation intervention vs Attention control  **Population:** 337 patients 60 years and older hospitalized for acute decompensated heart failure  **Follow-up:** 3 months  **Reference:** Pandey, 2023, US^46^ | Modified Fried frailty criteria (range: 0-5)  A: Frailty phenotype (1 to 2)  B: Frailty phenotype (3 to 5) | **SPPB score**  A: Effect size 0.8 (–0.1, 1.6)  B: Effect size 2.1 (1.3, 2.9) | Physical rehabilitation intervention was associated with greater improvement in SPPB score compared with attention control in patients with three or more frailty phenotypes, but not in patients with 1-2 components of the frailty phenotype (p-for-interaction=0.03). |  |
| **Intervention:** Physical exercise vs Usual care  **Population:** 299 adults aged 65 years and older  **Follow-up:** 24 months  **Reference:** Suikkanen, 2020, Finland^47^ | Modified Fried frailty criteria (range: 0-5)  A: Frailty phenotype (1 to 2)  B: Frailty phenotype (3 to 5) | **Days at home**  A: IRR 1.03 (0.96, 1.11)  B: IRR 1.04 (0.96, 1.12) | There was no evidence that the effect of physical exercise compared with usual care on days at home was different across the frailty spectrum (p-for-interaction=NR). |  |
| **Intervention:** Resistance-type exercise training vs No exercise training  **Population:** 127 adults aged 65 years or older with prefrail or frail  **Follow-up:** 24 weeks  **Reference:** Tieland, 2015, Netherland^48^ | Fried frailty criteria (range: 0-5)  A: Frailty phenotype (1 to 2)  B: Frailty phenotype (3 to 5) | **Dominant handgrip strength**  NR | There was no evidence that the effect of resistance-type exercise training compared with no exercise training on dominant handgrip strength was different across the frailty spectrum (p-for-interaction>0.05). |  |
| **Intervention:** Moderate-intensity physical activity vs Health education  **Population:** 1635 adults aged 70-89 years with functional limitations  **Follow-up:** 2 years  **Reference:** Trombetti, 2018, US^49^ | Study of Osteoporotic Fractures Index (range: 0-3)  A: Frailty phenotype (0 to <2)  B: Frailty phenotype (2 to 3) | **MMD**^‡^  A vs B: HR 0.92 vs HR 0.96  **PBD**^‡^  A vs B: HR 0.92 vs HR 0.94 | There was no evidence that the effect of moderate-intensive physical activity compared with health education on MMD (p-for-interaction=0.91) and PBD (p-for-interaction=0.64) was different across the frailty spectrum. |  |
| **Intervention:** Walking and nutrition vs walking vs Control  **Population:** 227 adults aged 65 years and older  **Follow-up:** 6 months  **Reference:** Yamada, 2015, Japan^50^ | Modified Cardiovascular Health Study criteria (range: 0-5)  A: Frailty phenotype (0 to 2)  B: Frailty phenotype (3 to 5) | **SMI**  A: 1.02% vs 1.11% vs -0.86%  B: 3.16% vs 0.64% vs -3.87%  **IGF-1**  A: 21.4% vs 22.5% vs 8.6%  B: 31.8% vs 14.5% vs 9.4%  **DHEA-S**  A: 26.6% vs 18.1% vs 8.4%  B: 15.9% vs 19.7% vs -0.8%  **25(OH)D**  A: 39.9% vs 32.0% vs 6.1%  B: 45.2% vs 33.6% vs -5.6% | There was no evidence that the effect of walking and nutrition or walking compared with control on SMI, IGF-1, DHEA-S, and 25(OH)D was different across the frailty spectrum (p-for-interaction=NR). |  |
| **Intervention:** High-dose exercise program vs Low-dose exercise program vs No exercise  **Population:** 110 adults aged over 65 years with sedentary behavior  **Follow-up:** 12 weeks  **Reference:** Kaushal, 2019, Canada^65^ | Combined Fried frailty phenotype, modified Physical Performance Test, and frailty index  Frail: meeting at least two of the three methods | **HR-QOL capacity**: NR | Higher dose exercise program was associated with greater improvement in capacity HR-QOL compared with no exercise in patients meeting at least two of the three frailty assessments (p-for-interaction=0.037). |  |
| **Intervention:** Physical activity vs health education  **Population:** 1635 adults aged 70-89 years  **Follow-up:** 3.5 years  **Reference:** Newman, 2016, US^66^ | SPPB score (range: 0-12)  A: Class 1 (0 to＜8)  B: Class 2 (8 to 9) | **Total cardiovascular disease event rates**  A: HR 0.76 (0.52, 1.10)  B: HR 1.59 (1.09, 2.30) | Physical activity was associated with higher total cardiovascular event rates compared with successful aging in patients with SPPB score 8 to 9, but not in patients with SPPB < 8 (p-for-interaction=0.006). |  |
| **Intervention:** Physical activity vs Health education program  **Population:** 1623 older persons with mobility limitations  **Follow-up:** 24 months  **Reference:** Custodero, 2023, US^61^ | Study of Osteoporotic Fractures frailty index (range: 0-3 criteria)  A: Class 1 (0 to 1)  B: Class 2 (2 to 3) | **400-m gait-speed**  (6-month)  A: mean difference 0.029 (0.017, 0.041)  B mean difference 0.027 (-0.001, 0.055)  (12-month)  A: mean difference 0.023 (0.011, 0.035)  B mean difference 0.014 (-0.015, 0.042)  (24-month)  A: mean difference 0.023 (0.010, 0.035)  B: mean difference 0.010 (-0.020, 0.039)  **4-m gait-speed**  (6-month)  A: mean difference -0.004 (-0.018, 0.011)  B: mean difference -0.011 (-0.041, 0.018)  (12-month)  A: mean difference -0.002 (-0.016, 0.013)  B: mean difference -0.008 (-0.039, 0.012)  (24-month)  A: mean difference -0.001 (-0.014, 0.016)  B: mean difference -0.010 (-0.022, 0.41) | There was no evidence that the effect of physical activity compared with health education on 400-m gait-speed, 4-m gait-speed was different across the frailty spectrum (p-for-interaction=NR). |  |
| **Non-pharmacological intervention – radiation therapy** | | | |  |
| **Intervention:** 1-week course radiation therapy vs 3-week course radiation  **Population:** 61 adults aged 65 years and older with glioblastoma  **Follow-up:** 2.5 years  **Reference:** Guedes de Castro, 2017, 10 countries^63^ | Karnofsky Performance Status (range: 50%-100%)  A: Class 1 (80%-100%)  B: Class 2 (50%-70%) | **OS**  A: 8.0 months (5.9, 10.0) vs 8.0 months (5.3, 10.3)  B: 7.5 months (5.3, 9.7) vs 6.7 months (4.5, 8.9) | There was no evidence that the effect of short-course radiation therapy compared with 3-week course radiation on OS was different across the frailty spectrum (p-for-interaction=NR). |  |
| **Non-pharmacological intervention – surgical procedures** | | | |  |
| **Intervention:** Anterior minimally invasive hemiarthroplasty vs Lateral Hardinge hemiarthroplasty  **Population:** 190 adults aged 60 years and older with femoral neck fractures  **Follow-up:** 12 months  **Reference:** Saxer, 2018, Switzerland^64^ | (18-(Functional Independence Measure-18)/6 + Charlson Index + Medication score)/36 (range: 0-1)  A: Frailty index (0 to ≤0.25)  B: Frailty index (0.25< to 1) | **Timed up and go duration:** NR | There was no evidence that anterior minimally invasive hemiarthroplasty compared with lateral Hardinge hemiarthroplasty on timed up and go duration was different across the frailty spectrum (p-for-interaction=NR). |  |
| **Non-pharmacological intervention – psychosocial intervention** | | | |  |
| **Intervention:** Psychosocial intervention vs Standard educational material on stroke recovery  **Population:** 291 adults aged over 45 years with stroke  **Follow-up:** 6 months (47 months for mortality)  **Reference:** Ertel, 2007, US^67^ | summary frailty index^c^ (range: 0-5):  A: Frailty index (0 to ≤3)  B: Frailty index (4 to 5) | **Instrumental activities of daily living score** (range: 0-14)  A: MD 1.11 (p=0.01)  B: MD -0.94 (p=0.09)  **Physical performance score** (range: 1-25)  A: MD 1.41 (p=0.11)  B: MD -1.56 (p=0.10)  **Global cognitive function score (standardized):**  A: MD 0.09 (p=0.25)  B: MD 0.01 (p=0.93)  **All-cause mortality:**  A: HR 0.40 (p=0.03)  B: HR 1.34 (p=0.27) | Psychosocial intervention was associated with improvement in instrumental activities of daily living (p-for-interaction<0.01), possible improvement in physical performance (p-for-interaction=0.02), and reduced mortality (p-for-interaction=0.01) compared with standard educational material in patients with frailty index 0-3, not in patients with frailty index 4-5. |  |
| **Non-pharmacological intervention – others** | | | |  |
| **Intervention:** Frailty screening vs Frailty screening + nurse-led care program vs usual care  **Population:** 3,092 adults aged 60 and older  **Follow-up:** 12 months  **Reference:** Bleijenberg, 2016, Netherlands^36^ | 50-item frailty index (range: 0-1)  A: Frailty index (0 to <0.2)  B: Frailty index (0.2≤ to 1) | **Modified Katz-15 score:** NR | There was no evidence that the effect of frailty screening alone or with nurse-led care program compared with usual care on modified Katz-15 score was difference across the frailty spectrum (p-for-interaction=NR). |  |
| **Intervention:** Home-based physiotherapy vs Hospital-based treatment rehabilitation  **Population:** 451 adults aged 62-81 years with stroke  **Follow-up:** 6 months  **Reference:** Gladman, 1995, UK^68^ | An operational definition of frailty based on:  (1) age > 80 years;  (2) living alone;  (3) previous disability;  (4) previous Functional Ambulation Category score < 5;  (5) not scoring above 6/10 on the AMTS;  (6) hospital stay >1 month or discharge Barthel < 15/20.  Frail: two or more criteria | **Function measures** (median Barthel score; median improvement in Barthel score; being able to walk outside)**:** NR | There was no evidence that the effect of Home-based physiotherapy compared with Hospital-based treatment rehabilitation on function measures was different across frailty spectrum (p-for-interaction=NR). |  |
| **Intervention:** Embrace^d^ vs usual care  **Population:** 1456 adults aged 75 years and older  **Follow-up:** 12 months  **Reference:** Spoorenberg, 2018, Netherlands^53^  Uittenbroek, 2017, Netherlands^54^ | INTERMED-E-SA (range: 0-20) and GFI (range: 0-15)  A: Class1 (INTERMED-E-SA: 16 to 20]  B: Class 2 (INTERMED-E-SA: 0 to <16 and GFI: 5 to 15)  C: Class 3 (INTERMED-E-SA: 0 to <16 and GFI: 0 to <5) | **EQ-5D-3L**  A: Effect size d=0.07  B: Effect size d=0.16  C: Effect size d=0.03  **PAIEC total score**  A: 0.44 (0.01, 0.87)  B: B 0.89 (0.42, 1.37)  C: B 0.13 (-0.07, 0.33)  Refer to Appendix Table for additional measures | There was no evidence that the effect of embrace compared with usual care on the domains of health and wellbeing was different across the frailty spectrum (p-for-interaction=NR).  Embrace was associated greater improvement in quality of life compared with usual care in patients with INTERMED-E-SA ≥ 16, and patients with INTERMED-E-SA <16 and GFI 5 to 15 (p-for-interaction=NR). |  |
| **Intervention:** Home-based intervention program^e^ vs Educational program  **Population:** 188 adults aged 75 years and older with physical frailty  **Follow-up:** 1 year  **Reference:** Gill, 2002, US^56^ | Rapid gait test and chair stand test  A: Class 1 (meeting one of two tests)  B: Class 2 (meeting both tests) | **Disability score (Adjusted)** (range: 0-16 points)  A: change 53% (p=0.005)  B: change 16% (p=0.5) | Home-based intervention program was associated with lower disability score compared with educational program in patients meeting one of two frailty assessments (p-for-interaction<0.001 at 7 months and p-for-interaction=0.005 at 12 months) |  |
| **Intervention:** HF program + home telemedicine^f^ vs HF program  **Population:** 178 patients aged 40-92 years with heart failure  **Follow-up:** 6 months  **Reference:** Comin-Colet, 2015, Spain^69^ | Frailty defined as:  age >=90 years  OR age 85–89 needing caregiver  OR moderate to severe dependency for ADL (Barthel Index < 90) at any age  OR moderate to severe cognitive impairment according to the Pfeiffer test at any age. | **Number of non-fatal HF events**  No frailty HR 0.33 (0.17, 0.64)  Frailty: HR 0.41 (0.16, 1.06) | There was no evidence that the effect of HF program and home telemedicine compared with HF program on non-fatal HF events was different across the frailty spectrum (p-for-interaction=0.838). |  |

### Table 3. Results from 8 Multicomponent intervention

| **Intervention, Population,**  **Reference** | **Frailty assessment** | **Frailty-Specific Results** | **Interpretation** |
| --- | --- | --- | --- |
| **Intervention:** GA-intervention^a^ vs usual-care  **Population:** 541 patients with incurable cancer and impairment on ≥1 GA domain  **Follow-up:** NR  **Reference:** Gilmore, 2021, US^39^ | 50-item frailty index (range: 0-1)  A: Frailty index (0≤ to <0.2)  B: Frailty index (0.2≤ to <0.35)  C: Frailty index (0.35≤ to 1) | **Conversations**  A: adjusted mean difference 3.27 (1.68, 4.86)  B: adjusted mean difference 3.75 (2.32, 5.18)  C: adjusted mean difference 4.03 (2.50, 5.56)  **Concerns**  **Acknowledged**  A: adjusted mean difference 2.08 (1.04, 3.12)  B: adjusted mean difference 1.87 (0.97, 2.77)  C: adjusted mean difference 2.27 (1.29, 3.25)  **Concerns**  **Addressed**  A: adjusted mean difference 2.03 (0.87, 3.19)  B: adjusted mean difference 2.13 (1.05, 3.21)  C: adjusted mean difference 2.19 (1.07, 3.31) | There was no evidence that the effect of GA-intervention compared with usual care on conversations (p-for-interaction=0.6111), concerns acknowledged (p-for-interaction=0.7397), concerns addressed (p-for-interaction=0.9403) was different across the frailty spectrum. |
| **Intervention:** Pharmacist-led deprescribing intervention^b^ vs Usual care  **Population:** 338; 295  **Follow-up:** 6months  **Reference:** Jamieson, 2023, New Zealand^70^; Nishtala, 2023, New Zealand^71^ | 15-item frailty index (range: 0-1)  A: Low-frailty  B: Medium-frailty  C: High-frailty | **Changes in DBI 0.5 or more**  A: difference 9.85% (-3.94%, 23.48%)  B: difference -5.45% (-17.25%, 6.35%)  C: difference -5.13% (-22.08%, 11.82%)  **ACB**  A: mean change -0.02 (-0.65, 0.18)  B: mean change 0.05 (-0.28, 0.38)  C: mean change 0.08 (-0.40, 0.56) | There was no evidence that the effect of pharmacist-led deprescribing intervention compared with usual care on changes in DBI 0.5 or more, ACB was different across the frailty spectrum (p-for-interaction=NR). |
| **Intervention:** ①MI^b^ + n3 PUFA^c^ vs Placebo;  ②n3 PUFA vs Placebo;  ③MI + Placebo vs Placebo  **Population:** 1680 adults aged 70 years and older without dementia but with subjective memory complaints  **Follow-up:** 3 years  **Reference:** Tabue-teguo, 2018, France^20^ | Fried frailty criteria (range: 0-5)  A: Frailty phenotype (0)  B or C: Frailty phenotype (1 to 5) | **3-year TMTA Difference of score change**  ①Pre-frail: -4.029  Non-frail: 1.134  ②Pre-frail: 0.246  Non-frail: 3.478  ③Pre-frail: 2.268  Non-frail: 2.951  **3-year TMTB Difference of score change**  ①Pre-frail: -4.093  Non-frail: -1.466  ②Pre-frail: -6.313  Non-frail: 1.798  ③Pre-frail: 12.891  Non-frail: -4.297  Refer to Appendix Table for more outcomes | There was no evidence that the effect of MI and n3 PUFA program compared with placebo on cognitive function was different across the frailty spectrum (p-for-interaction>0.05). |
| **Intervention:** Multifactorial, interdisciplinary intervention^d^ vs Usual care  **Population:** 241 adults aged 70 years and older without severe cognitive impairment  **Follow-up:** 1 year  **Reference:** Fairhall, 2012, Australia^51^  Fairhall, 2014, Australia^52^ | Modified CHS frailty criteria (range: 0-5)  A: Frailty phenotype (3)  B: Frailty phenotype (4 to 5) | **Life Space Assessment score**  (3 months)  A: 9.0 (5.3, 12.7)  B: 2.0 (-2.8, 6.8)  (12 months)  NR  **Gait speed**  (3 months)  NR  (12 months)  A 0.01 m/s (-0.05, 0.07)  B 0.13 m/s (0.04, 0.22)  **SPPB**: NR  **PPA**: NR | Multifactorial, interdisciplinary intervention was associated with greater life space compared with usual care in patients with 3 components of the frailty phenotype at 3 months (p-for-interaction=0.03), but no longer effective at 12 months (p-for-interaction=0.4).  Multifactorial, interdisciplinary intervention was associated with greater gait speed compared with usual care in patients with 4-5 components of the frailty phenotype at 12 months (p-for-interaction=0.03), but not effective at 3 months (p-for-interaction=0.9).  There was no evidence that multifactorial, interdisciplinary intervention compared with usual care on SPPB and PPA was different across frailty spectrum (p-for-interaction=NR). |
| **Intervention:** DMP vs Usual care  **Population:** 173 adults aged over 70 years with heart failure  **Follow-up:** 2 years  **Reference:** Pulignano, 2010, Italy^19^ | Modified frailty score^g^  (range: 1-6)  A: Class1 (1)  B: Class 2 (2)  C: Class 3 (3)  D: Class 4 (4 to 6) | **Death and/or heart failure admissions**  A: HR 2.506 (0.459, 13.687)  B: HR 0.441 (0.226, 0.859)  C: HR 0.426 (0.195, 0.932)  D: HR 0.369 (0.153, 0.891)  **All-cause admissions**  A: HR 1.905 (0.723, 5.024)  B: HR 0.490 (0.268, 5.894)  C: HR 0.285 (0.124, 0.650)  D: HR 0.467 (0.200, 1.089) | There was no evidence that the effect of DMP compared with usual care on death and/or heart failure admissions was different across the frailty spectrum (p-for-interaction=0.208).  DMP was associated with lower all-cause admissions compared with usual care in patients with frailty score 2 to 3, but not in patients with frailty score <1 or frailty score ≥ 4 (p-for-interaction=0.0178). |
| **Intervention:** POC approach^h^ vs Usual care  **Population:** 346 community dwelling frail adults aged 70 years and older  **Follow-up:** 24 months  **Reference:** Metzelthin, 2013, Netherland^55^ | GFI (range: 0-15)  A: Class 1 (5 to 6)  B: Class 2 (7 to 14) | **Groningen Activity Restriction Scale (range total scale 18-78) at 6, 12, and 24 months**  NR | There was no evidence that the effect of POC approach compared with usual care on Groningen Activity Restriction Scale was different across the frailty spectrum (p-for-interaction>0.05). |

## Appendix 5. Detailed summary of included trials

| ***Akashi et al. Outcomes and Safety of Very-Low-Dose Edoxaban in Frail Patients With Atrial Fibrillation in the ELDERCARE-AF Randomized Clinical Trial. JAMA Netw Open. 2022 Aug 1;5(8):e2228500. doi: 10.1001/jamanetworkopen.2022.28500. https://pubmed.ncbi.nlm.nih.gov/35997978/*** | | | | |
| --- | --- | --- | --- | --- |
| **Study design** | **Study type** | **Country/ Region** | **Clinical setting** | **Duration** |
|  | Secondary analysis of the randomized controlled trial | Japan | NR | August 5 2016 - November 5 2019 |
|  | **Intervention vs Control** | | **Sample size** | **Female (%)** |
|  | Very-low-dose edoxaban vs placebo | | 984 | 57.3 |
|  | **Age, years** | **Conditions** | **Follow-up** | **Loss to follow-up (%)** |
|  | Mean: 86.6 (range: 80+) | Patients with atrial fibrillation (AF) aged 80 years or older | 4 months | 4.07% |
| **Frailty assessment** | **Measurement tool** | **Frailty assessment** | | **Frailty at baseline, Intervention vs Control** |
|  | Frailty phenotype | Fried frailty criteria (range: 0-5 criteria): Nonfrail [<3 criteria] (N=542) Frail [≥ 3 criteria] (N=402) | | Nonfrail: 60.97% vs 53.83% Frail: 30.03% vs 46.17% |
| **Main findings** | **Outcome** | **Relative effect** | **Analytic approach** | ***P*-value** |
|  | 4-month Stroke or systemic embolism | Nonfrail: HR 0.24 (0.10-0.59) Frail: HR 0.35 (0.14-0.87) | Stratified analysis and interaction term | P for interaction = 0.55 |
|  | 4-month All-cause death | Nonfrail: HR 1.46 (0.85-2.51) Frail: HR 0.72 (0.45-1.18) |  | P for interaction = 0.06 |
|  | 4-month Net clinical composite outcome | Nonfrail: HR 0.99 (0.65-1.51) Frail: HR 0.77 (0.50-1.17) |  | P for interaction = 0.42 |

Abbreviations: NR, not reported

| ***Bleijenberg et al. Effectiveness of a Proactive Primary Care Program on Preserving Daily Functioning of Older People: A Cluster Randomized Controlled Trial. J Am Geriatr Soc. 2016;64(9):1779-1788. https://pubmed.ncbi.nlm.nih.gov/27459236/*** | | | | |
| --- | --- | --- | --- | --- |
| **Study design** | **Study type** | **Country/ Region** | **Clinical setting** | **Duration** |
|  | Randomized controlled trial | Netherlands | 39 general practices | October 1, 2010-January 15, 2012* |
|  | **Intervention vs Control** | | **Sample size** | **Female (%)** |
|  | Frailty Screening vs Screening + nurse-led care program vs usual care | | 3092 | 55.2 |
|  | **Age, years** | **Conditions** | **Follow-up** | **Loss to follow-up (%)** |
|  | Mean: 74.2 (range: 60+) | Community-dwelling people | 12 months | 19.5% |
| **Frailty assessment** | **Measurement tool** | **Frailty assessment** | | **Frailty at baseline, Intervention vs Control** |
|  | Other frailty assessment tools | Frailty index (range: 0-1 points); Groningen Frailty Indicator questionnaire. NSD | | Frailty index: Screening vs Screening + nurse-led care vs Usual care: 0.06 vs 0.08 vs 0.08 |
| **Main findings** | **Outcome** | **Relative effect** | **Analytic approach** | ***P*-value for interaction** |
|  | 12-month Secondary outcome | NR | NR | NR |
| **Risk of bias** | **Random sequence generation** | **Allocation concealment** | **Blinding of participants and personnel** | **Blinding of outcome assessment** |
|  | Low | Low | Low | Low |
|  | **Incomplete outcome data** | **Selective reporting** | **Other bias** |  |
|  | High | Low | Low |  |

Abbreviations: NSD, no sufficient data; NR, not reported; QoL, quality of life.

Notes: The secondary outcomes were self-reported health-related quality of life (QoL), perceived QoL score, satisfaction with primary care, number of hospital admission, and admission to a nursing home or assisted-living facility, and general practice out-of-hours consultations during follow-up.

"Reference:

*: Clinical Trial Registration: NTR2288. Available at https://www.trialregister.nl/trial/2164."

| ***Bringhen et al. Lenalidomide-based induction and maintenance in elderly newly diagnosed multiple myeloma patients: updated results of the EMN01 randomized trial. Haematologica. 2020;105(7):1937-1947. https://pubmed.ncbi.nlm.nih.gov/31582542/*** | | | | |
| --- | --- | --- | --- | --- |
| **Study design** | **Study type** | **Country/ Region** | **Clinical setting** | **Duration** |
|  | Secondary analysis of the randomized controlled trial | Italian and Czech Republic | 67 centers | October 2009-November 2022* |
|  | **Intervention vs Control** | | **Sample size** | **Female (%)** |
|  | Induction treatments: MPR vs CPR vs RD  Maintenance treatments: R vs RP | | 654 | 51.4 |
|  | **Age, years** | **Conditions** | **Follow-up** | **Loss to follow-up (%)** |
|  | Median: 73 (range: 65+) | Newly diagnosed MM patients | Median: 71 months | 38.5%† |
| **Frailty assessment** | **Measurement tool** | **Frailty assessment** | | **Frailty at baseline, Intervention vs Control** |
|  | Other frailty assessment tool | Frailty Score (range: 0-5 scores)† Fit [0] (N=285) Intermediate-fitness [1] (N=206) Frail [≥2] (N=168) | | R vs RP: Fit: 50% vs 46% Intermediate-fit: 31% vs 29% Frail: 20% vs 25% |
| **Main findings** | **Outcome** | **Relative effect** | **Analytic approach** | **p-value for interaction** |
|  | 70-month PFS | MPR vs CPR: Fit: HR 0.72 [0.52, 1.00] Intemediate fit: HR 0.80 [0.56, 1.14] Frail: HR 0.88 [0.57, 1.34] MPR vs Rd: Fit: HR 0.72 [0.52, 0.99] Intermediate fit: HR 0.82 [0.56, 1.20] Frail: HR 1.18 [0.78, 1.80] | Stratified analysis | NA |
|  | 70-month OS | MPR vs CPR: Fit: HR 1.11 [0.72, 1.71] Intemediate fit: HR 1.11 [0.72, 1.71] Frail: HR 0.91 [0.54, 1.51] MPR vs Rd: Fit: HR 0.75 [0.50, 1.12] Intermediate fit: HR 1.25 [0.75, 2.09] Frail: HR 1.13 [0.69, 1.86] |  |  |

Abbreviations: MPR, melphalan-prednisone-lenalidomide; CPR, cyclophosphamide-prednisone-lenalidomide; RD, lenalidomide plus low-dose dexamethasone; R, Lenalidomide; RP, lenalidomide-Prednisone; MM, multiple myeloma; PFS, progression-free survival; OS, overall survival; NA, not applicable.

Notes: Duration was study start date to estimated study completion date. The analysis based different frailty subgroups so that interaction p value not applicable.

"Reference:

*: Clinical Trial Registration: NCT01093196. Avaliable at https://clinicaltrials.gov/ct2/show/NCT01093196?term=NCT01093196&draw=2&rank=1.

†: Original RCT: Magarotto et al. Triplet vs doublet lenalidomide-containing regimens for the treatment of elderly patients with newly diagnosed multiple myeloma. Blood. 2016;127(9):1102-1108.

‡: Supplement data. Avaliable at https://ashpublications.org/blood/article/127/9/1102/126446/Triplet-vs-doublet-lenalidomide-containing."

| ***Brioli, et al. Frailty impairs the feasibility of induction therapy but not of maintenance therapy in elderly myeloma patients: final results of the German Maintenance Study (GERMAIN). J Cancer Res Clin Oncol. 2020;146(3):749-759. https://pubmed.ncbi.nlm.nih.gov/31788741/*** | | | | |
| --- | --- | --- | --- | --- |
| **Study design** | **Study type** | **Country/ Region** | **Clinical setting** | **Duration** |
|  | Randomized controlled trial | Germany | NR | August 2013-December 31, 2018† |
|  | **Intervention vs Control** | | **Sample size** | **Female (%)** |
|  | Lenalidomide vs Placebo | | 40 | 32.8 |
|  | **Age, years** | **Conditions** | **Follow-up** | **Loss to follow-up (%)** |
|  | Median: 75 (range: 63-87) | Transplant-ineligible patients with untreated newly diagnosed symptomatic MM. | Median: 12.9 months | 52.5% |
| **Frailty assessment** | **Measurement tool** | **Frailty assessment** | | **Frailty at baseline, Intervention vs Control** |
|  | Other frailty assessment tool | International Myeloma Working Group (IMWG) score: Fit (N=11) Intermediate fitness (N=10) Frail (N=19) NSD | | Fit: 26% vs 29% Intermediate fitness: 16% vs 33% Frail: 58% vs 38% |
| **Main findings** | **Outcome** | **Relative effect** | **Analytic approach** | **p-value for interaction** |
|  | PFS | NR | NR | NR. There was no statistical difference in terms of PFS and OS depending on the frailty of patients. |
|  | OS |  |  |  |
| **Risk of bias** | **Random sequence generation** | **Allocation concealment** | **Blinding of participants and personnel** | **Blinding of outcome assessment** |
|  | Unclear | Unclear | Low | Low |
|  | **Incomplete outcome data** | **Selective reporting** | **Other bias** |  |
|  | High | Low | Low |  |

Abbreviations: MM, multiple myeloma; PFS, progression-free survival; OS, overall survival; NA, not applicable; NSD, no sufficient data.

Reference:

*: Supplement data. Available at https://link.springer.com/article/10.1007/s00432-019-03101-z#Sec13.

†: Clinical Trial Registration: NCT02145598. Available at https://clinicaltrials.gov/ct2/show/NCT02145598?term=NCT02145598&draw=2&rank=1.

| ***Butt et al. Efficacy and Safety of Dapagliflozin According to Frailty in Heart Failure With Reduced Ejection Fraction : A Post Hoc Analysis of the DAPA-HF Trial. Ann Intern Med. 2022 Jun;175(6):820-830. doi: 10.7326/M21-4776. Epub 2022 Apr 26.***  ***https://pubmed.ncbi.nlm.nih.gov/35467935/*** | | | | |
| --- | --- | --- | --- | --- |
| **Study design** | **Study type** | **Country/ Region** | **Clinical setting** | **Duration** |
|  | Secondary analysis of the randomized controlled trial | 20 countries | 410 sites | February 8, 2017 - July 17, 2019* |
|  | **Intervention vs Control** | | **Sample size** | **Female (%)** |
|  | Dapagliflozin vs Placebo | | 4742 | 23.4 |
|  | **Age, years** | **Conditions** | **Follow-up** | **Loss to follow-up (%)** |
|  | Mean:  Not frail: 63.6  More frail: 68.8  Most frail: 69.8 | Patients with symptomatic heart failure with a left ventricular ejection fraction of 40% or less and elevated natriuretic peptide. | Median: 18.2 months | NA. Subset of the original RCT. |
| **Frailty assessment** | **Measurement tool** | **Frailty assessment** | | **Frailty at baseline, Intervention vs Control** |
|  | Frailty index | Rockwood cumulative deficit approach (32-item Frailty Index, range: 0-1) Class 1 frailty, not frail [FI ≤0.210] (N=2392) Class 2 frailty, more frail [FI 0.211-0.310] (N=1606) Class 3 frailty, most frail [FI ≥0.311] (N=744) | | Mean FI: 0.216±0.091 |
| **Main findings** | **Outcome** | **Relative effect** | **Analytic approach** | ***P*-value** |
|  | 18-month Worsening HF event or cardiovascular death | Not frail: HR 0.72 (0.59, 0.89)  More frail: HR 0.77 (0.62, 0.97)  Most frail: HR 0.71 (0.54, 0.93) | Stratified analysis | NR |
|  | 18-month HF hospitalization or cardiovascular death | Not frail: HR 0.71(0.58, 0.88)  More frail: HR 0.79 (0.63, 0.99)  Most frail: HR 0.72 (0.55, 0.94) |  |  |
|  | 18-month HF hospitalization | Not frail: HR 0.63 (0.48,0.83)  More frail: HR 0.79 (0.59, 1.07)  Most frail: HR 0.68 (0.49, 0.94) |  |  |
|  | 18-month Cardiovascular death | Not frail: HR 0.74 (0.56, 0.98)  More frail: HR 0.83 (0.62, 1.10)  Most frail: HR 0.97 (0.67, 1.40) |  |  |
|  | 18-month Noncardiovascular death | Not frail: HR 0.70 (0.35, 1.38)  More frail: HR 1.18 (0.62, 2.24)  Most frail: HR 0.72 (0.37, 1.43) |  |  |
|  | 18-month All-cause death | Not frail: HR 0.73 (0.57, 0.95)  More frail: HR 0.88 (0.67, 1.14)  Most frail: HR 0.91 (0.66, 1.26) |  |  |
|  | 18-month Recurrent HF hospitalization or cardiovascular death | Not frail: Rate ratio 0.67 (0.53, 0.85)  More frail: Rate ratio 0.83 (0.65, 1.06)  Most frail: Rate ratio 0.79 (0.59, 1.06) |  |  |
|  | 18-month New-onset type 2 diabetes | Not frail: HR 0.64 (0.41, 1.02)  More frail: HR 0.67 (0.40, 1.12)  Most frail: HR 0.87 (0.36, 2.10) |  |  |

Abbreviations: NA, not available; HF, heart failure; NR, not reported.

References:

*: Clinical Trial Registration: NCT03036124. Available at https://classic.clinicaltrials.gov/ct2/show/NCT03036124?cond=NCT03036124&draw=2&rank=1.

| ***Butt et al. Sacubitril/Valsartan and Frailty in Patients With Heart Failure and Preserved Ejection Fraction. J Am Coll Cardiol. 2022 Sep 20;80(12):1130-1143. doi: 10.1016/j.jacc.2022.06.037. Epub 2022 Aug 29. https://pubmed.ncbi.nlm.nih.gov/36050227/*** | | | | |
| --- | --- | --- | --- | --- |
| **Study design** | **Study type** | **Country/ Region** | **Clinical setting** | **Duration** |
|  | Randomized controlled trial | United Kingdom | trial center | NR |
|  | **Intervention vs Control** | | **Sample size** | **Female (%)** |
|  | Sacubitril/valsartan vs valsartan | | 4795 | 51.7 |
|  | **Age, years** | **Conditions** | **Follow-up** | **Loss to follow-up (%)** |
|  | Mean:  not frail 71.3  more frail 73.8  most frail 74.6 (range: 50+) | Patients with heart failure with preserved ejection fraction | 4 years | NA. Subset of the original RCT. |
| **Frailty assessment** | **Measurement tool** | **Frailty assessment** | | **Frailty at baseline, Intervention vs Control** |
|  | Frailty index | Rockwood cumulative deficit approach Class 1 frailty, not frail [FI ≤0.210] (N=2165) Class 2 frailty, more frail [FI 0.211-0.310] (N=2084) Class 3 frailty, most frail [FI ≥0.311] (N=546) | | Mean FI: 0.227±0.091 |
| **Main findings** | **Outcome** | **Relative effect** | **Analytic approach** | ***P*-value** |
|  | 4-year Primary outcome | FI class 1: RR 0.98 (0.76, 1.27) FI class 2: RR 0.92 (0.76, 1.12) FI class 3: RR 0.69 (0.51, 0.95) | Interaction term | When FI is treated as a continuous variable, the p-values for the interaction: < 0.002. |
|  | 4-year Total heart failure hospitalizations | FI class 1: RR 0.95 (0.71 to 1.29) FI class 2: RR 0.93 (0.75 to 1.16) FI class 3: RR 0.64 (0.45 to 0.91) | Interaction term | When FI is treated as a continuous variable, the p-values for the interaction: < 0.001. |
| **Risk of bias** | **Random sequence generation** | **Allocation concealment** | **Blinding of participants and personnel** | **Blinding of outcome assessment** |
|  | Low | Low | Low | Low |
|  | **Incomplete outcome data** | **Selective reporting** | **Other bias** |  |
|  | Low | Low | Unclear |  |

Abbreviations: NR, not reported

Note: The primary outcome in PARAGON-HF was a composite of total (first and recurrent) HF hospitalizations or cardiovascular death.

| ***Butt et al. Efficacy and Safety of Dapagliflozin According to Frailty in Patients With Heart Failure: A Prespecified Analysis of the DELIVER Trial. Circulation. 2022 Oct 18;146(16):1210-1224. doi: 10.1161/CIRCULATIONAHA.122.061754. Epub 2022 Aug 27. https://pubmed.ncbi.nlm.nih.gov/36029465/*** | | | | |
| --- | --- | --- | --- | --- |
| **Study design** | **Study type** | **Country/ Region** | **Clinical setting** | **Duration** |
|  | Randomized controlled trial | United Kingdom | trial center | NR |
|  | **Intervention vs Control** | | **Sample size** | **Female (%)** |
|  | Dapagliflozin vs placebo | | 6258 | 43.9 |
|  | **Age, years** | **Conditions** | **Follow-up** | **Loss to follow-up (%)** |
|  | Mean:  not frail 70.1 vs more frail 72.6 vs most frail 72.7 (range: 40+) | Patients with heart failure and mildly reduced or preserved left ventricular ejection fraction (LVEF) | 3 years | NA. Subset of the original RCT. |
| **Frailty assessment** | **Measurement tool** | **Frailty assessment** | | **Frailty at baseline, Intervention vs Control** |
|  | Frailty index | Rockwood cumulative deficit approach Class 1 frailty, not frail [FI ≤0.210] (N=2354) Class 2 frailty, more frail [FI 0.211-0.310] (N=2413) Class 3 frailty, most frail [FI ≥0.311] (N=1491) | | Mean FI: 0.248±0.092 |
| **Main findings** | **Outcome** | **Relative effect** | **Analytic approach** | ***P*-value** |
|  | 3-year The effect of dapagliflozin on the primary end point | FI class 1: HR 0.85 (0.68, 1.06) FI class 2: HR 0.89 (0.74, 1.08) FI class 3: HR 0.74 (0.61, 0.91) | Stratified analysis and interaction term | P for interaction = 0.40 |
| **Risk of bias** | **Random sequence generation** | **Allocation concealment** | **Blinding of participants and personnel** | **Blinding of outcome assessment** |
|  | Low | Low | Low | Low |
|  | **Incomplete outcome data** | **Selective reporting** | **Other bias** |  |
|  | Low | Low | Unclear |  |

Abbreviations: NR, not reported

Note: The primary end point was time to a first worsening heart failure event or cardiovascular death.

| ***Coelho-Júnior, et al. Effects of Low-Speed and High-Speed Resistance Training Programs on Frailty Status, Physical Performance, Cognitive Function, and Blood Pressure in Prefrail and Frail Older Adults. Front Med (Lausanne). 2021;8:702436. Published 2021 Jul 26. https://pubmed.ncbi.nlm.nih.gov/34381802/*** | | | | |
| --- | --- | --- | --- | --- |
| **Study design** | **Study type** | **Country/ Region** | **Clinical setting** | **Duration** |
|  | Randomized controlled trial | Brail | Senior center and nursing home | October 1, 2017-August 29, 2019* |
|  | **Intervention vs Control** | | **Sample size** | **Female (%)** |
|  | low-speed resistance training vs high-speed resistance training vs control | | 60 | 81.7 |
|  | **Age, years** | **Conditions** | **Follow-up** | **Loss to follow-up (%)** |
|  | Mean: 70.5 (range: 60-76) | Prefrail and frail adluts | NR | 23.1% |
| **Frailty assessment** | **Measurement tool** | **Frailty assessment** | | **Frailty at baseline, Intervention vs Control** |
|  | Frailty phenotype | Fried frailty criteria (range: 0-5 criteria): Prefrail [1-2 of the 5 criteria] (N=32) Frail [≥ 3 criteria] (N=28) | | Prefrail: 57.9% vs 50% vs 52.6% Frail: 42.1% vs 50% vs 47.4% |
| **Main findings** | **Outcome** | **Relative effect** | **Analytic approach** | ***P*-value for interaction** |
|  | Right IHG, kg | Prefrail: mean 27.4 vs 20.7 vs 25.7  Frail: mean 9.0 vs 7.0 vs 13.6 | Stratified analysis | NR |
|  | Left IHG, kg | Prefrail: mean 27.3 vs 20.0 vs 25.6 Frail: mean 11.2 vs 12.3 vs 13.7 |  |  |
|  | Right knee extensor, kgf | Prefrail: mean 19.2 vs 13.5 vs 9.8 Frail: mean 10.6 vs 7.9 vs 6.8 |  |  |
|  | Left knee extensor, kgf | Prefrail: mean 16.8 vs 14.3 vs 9.7 Frail: mean 9.7 vs 9.2 vs 6.2 |  |  |
|  | Right hip flexor, kgf | Prefrail: mean 12.8 vs 9.1 vs 8.1 Frail: mean 7.4 vs 6.6 vs 5.0 |  |  |
|  | Left hip flexor, kgf | Prefrail: mean 12.5 vs 8.7 vs 8.2 Frail: mean 6.8 vs 7.1 vs 4.7 |  |  |
|  | Right ankle extensor, kgf | Prefrail: mean 8.7 vs 7.6 vs 5.6 Frail: mean 6.1 vs 4.3 vs 3.8 |  |  |
|  | Left ankle extensor, kgf | Prefrail: mean 8.7 vs 7.5 vs 6.2 Frail: mean 4.5 vs 5.9 vs 3.2 |  |  |
|  | Right one-leg stand, s (30s max) | Prefrail: mean 6.6 vs 14.2 vs 11.9 Frail: mean 1.0 vs 2.0 vs 2.8 |  |  |
|  | Left one-leg stand, s (30s max) | Prefrail: mean 5.5 vs 17.9 vs 9.9 Frail: mean 0.2 vs 1.7 vs 3.3 |  |  |
|  | Normal balance, s (10s max) | Prefrail: mean 10.0 vs 10.0 vs 10.0 Frail: mean 2.5 vs 2.7 vs 4.4 |  |  |
|  | Semi tandem balance, s (10s max) | Prefrail: mean 10.0 vs 10.0 vs 10.0 Frail: mean 1.2 vs 1.8 vs 4.4 |  |  |
|  | Tandem balance, s (10s max) | Prefrail: mean 10.0 vs 7.3 vs 10.0 Frail: mean 1.2 vs 0.9 vs 1.1 |  |  |
|  | Sit-to-stand, s | Prefrail: mean 6.6 vs 7.3 vs 9.0 Frail: mean 17.1 vs 18.9 vs 37.1 |  |  |
|  | Sit-to-stand, power | Prefrail: mean 54.2 vs 42.1 vs 43.9 Frail: mean 28.5 vs 27.5 vs 12.8 |  |  |
|  | Sit-to-stand, concentric contraction, m/s | Prefrail: mean 1.7 vs 1.2 vs 1.1 Frail: mean 0.55 vs 0.50 vs 0.19 |  |  |
|  | Sit-to-stand, eccentric contraction, m/s | Prefrail: mean 1.0 vs 1.0 vs 1.3 Frail: mean 0.65 vs 0.56 vs 0.86 |  |  |
|  | TUG at usual pace, s | Prefrail: mean 7.5 vs 9.4 vs 6.3 Frail: mean 64.2 vs 23.9 vs 48.7 |  |  |
|  | TUG at fast pace, s | Prefrail: mean 6.1 vs 7.8 vs 6.0 Frail: mean 45.0 vs 16.7 vs 25.8 |  |  |
|  | TUG with verbal task, s | Prefrail: mean 7.4 vs 9.1 vs 7.4 Frail: mean 52.0 vs 20.8 vs 38.3 |  |  |
|  | TUG with motor task, s | Prefrail: mean 7.8 vs 9.4 vs 8.2 Frail: mean 22.2 vs 5.9 vs 13.7 |  |  |
|  | TUG with both verbal and motor tasks, s | Prefrail: mean 7.7 vs 9.8 vs 11.1 Frail: mean 29.6 vs 6.0 vs 17.1 |  |  |
|  | WS at usual pace, m/s | Prefrail: mean 1.5 vs 1.4 vs 1.2 Frail: mean 0.41 vs 0.48 vs 0.58 |  |  |
|  | WS at fast pace, m/s | Prefrail: mean 1.8 vs 1.4 vs 2.1 Frail: mean 0.48 vs 0.61 vs 0.65 |  |  |
|  | 6MWT, m | Prefrail: mean 511 vs 478 vs 589 Frail: NR |  |  |
| **Risk of bias** | **Random sequence generation** | **Allocation concealment** | **Blinding of participants and personnel** | **Blinding of outcome assessment** |
|  | Low | Low | High | Low |
|  | **Incomplete outcome data** | **Selective reporting** | **Other bias** |  |
|  | High | Low | Low |  |

Abbreviations: NR, not reported; 6MWT, 6-min walking test; IHG, Isometric handgrip strength; TUG, Timed "Up and Go"; WS, Walking speed.

Note: The relative effects were not reported.

"References:

*: Clinical Trial Registration: NCT04868071. Available at https://clinicaltrials.gov/ct2/show/NCT04868071?term=NCT04868071&draw=2&rank=1."

| ***Comín-Colet, et al. Impact on clinical events and healthcare costs of adding telemedicine to multidisciplinary disease management programmes for heart failure: Results of a randomized controlled trial. J Telemed Telecare. 2016;22(5):282-295. https://pubmed.ncbi.nlm.nih.gov/26350543/*** | | | | |
| --- | --- | --- | --- | --- |
| **Study design** | **Study type** | **Country/ Region** | **Clinical setting** | **Duration** |
|  | Randomized controlled trial | Spain* | NR | December 2010- December 2014* |
|  | **Intervention vs Control** | | **Sample size** | **Female (%)** |
|  | HF programme + telemedicine vs HF programme | | 178 | 41.0 |
|  | **Age, years** | **Conditions** | **Follow-up** | **Loss to follow-up (%)** |
|  | Mean: 74 (range: 18+) | Patients with HF. | 6 months | 0.0% |
| **Frailty assessment** | **Measurement tool** | **Frailty assessment** | | **Frailty at baseline, Intervention vs Control** |
|  | Other frailty assessment tool | Frailty was defined according to the following criteria: age≥90 years or age 85–89 needing caregiver or moderate to severe dependency for basic activities of daily living (Barthel Index<90) at any age or moderate to severe cognitive impairment according to the Pfeiffer test at any age. | | Frailty: 24% vs 26% |
| **Main findings** | **Outcome** | **Relative effect** | **Analytic approach** | ***P*-value for interaction** |
|  | number of non-fatal HF events in 6 months | Frailty: 0.41 [0.16, 1.06] No frailty 0.33 [0.17, 0.64]† | Stratified analysis and interaction term | 0.838† |
| **Risk of bias** | **Random sequence generation** | **Allocation concealment** | **Blinding of participants and personnel** | **Blinding of outcome assessment** |
|  | Low | Low | High | High |
|  | **Incomplete outcome data** | **Selective reporting** | **Other bias** |  |
|  | Low | Low | Low |  |

Abbreviations: NR, not reported; HF, heart failure

Note: †The figure is blury.

Reference:

*: Clinical Trial Registration: NCT01495078. Available at https://clinicaltrials.gov/ct2/show/NCT01495078?term=NCT01495078&draw=2&rank=1.

| ***Curran, et al. Recombinant Zoster Vaccine Is Efficacious and Safe in Frail Individuals. J Am Geriatr Soc. 2021;69(3):744-752. https://pubmed.ncbi.nlm.nih.gov/33197294/*** | | | | |
| --- | --- | --- | --- | --- |
| **Study design** | **Study type** | **Country/ Region** | **Clinical setting** | **Duration** |
|  | Secondary analysis of two RCTs | 17 countries* | 169 investigational site* | June 5, 2018-April 30, 2019* |
|  | **Intervention vs Control** | | **Sample size** | **Female (%)** |
|  | Adjuvanted recombinant zoster vaccine vs placebo | | 29305 | 58.1 |
|  | **Age, years** | **Conditions** | **Follow-up** | **Loss to follow-up (%)** |
|  | Mean: 68.8 (range: 50+) | NR | NR | NA |
| **Frailty assessment** | **Measurement tool** | **Frailty assessment** | | **Frailty at baseline, Intervention vs Control** |
|  | 41-item Frailty index | Frailty index (range: 0-1 points) Non-frail [0, 0.08] (N=11518) Pre-frail (0.08, 0.25] (N=12290) Frail (0.25, 1] (N=3037) | | NR |
| **Main findings** | **Outcome** | **Absolute effect†** | **Analytic approach** | ***P*-value for interaction** |
|  | 36-month Vaccine efficacy (%) | Non-frail: 95.8  Pre-frail: 90.4  Frail: 90.2 | Stratified analysis | Not available |
|  | 36-month Vaccine reponse rate for anti-gE antibody ELISA concentrations (%) | Non-frail: 81.4 vs 1.8 Pre-frail: 74 vs 2.2 Frail: 61.6 vs 7.1 |  |  |
|  | 36-month Vaccine reponse rate for gE-specific CD42+ T-cells (%) | Non-frail: 58.4 vs 0 Pre-frail: 48.3 vs 0 Frail: 32.8 vs 13.5 |  |  |
|  | 36-month Anti-gE antibody GMC (mIU/mL) | Non-frail: 12475.2 Pre-frail: 11089.1 Frail: 8712.9 |  |  |
|  | 36-month CD42+ cells/106 CD4 T cells (Median) | Non-frail: 825 Pre-frail: 693 Frail: 528 |  |  |

Abbreviations: RCT, randomized controlled trial; NR, not reported; NA, not applicable.

Notes: United States; Australia; Brazil; Canada; Czechia; Finland; France; Germany; Hong Kong; Italy; Japan; Korea, Republic of; Mexico; Spain; Sweden; Taiwan; and United Kingdom. The relative effects were not reported.

Reference:

*: Clinical Trial Registration: NCT03563183. Available at https://clinicaltrials.gov/ct2/show/NCT03563183?term=NCT03563183&draw=2&rank=1.

†: Data were extracted by GetData Graph Digitizer software.

Original RCT-1: Lal H, et al. Efficacy of an adjuvanted herpes zoster subunit vaccine in older adults. N Engl J Med. 2015;372(22):2087-2096.

Original RCT-2: Cunningham AL, et al. Efficacy of the Herpes Zoster Subunit Vaccine in Adults 70 Years of Age or Older. N Engl J Med. 2016;375(11):1019-1032.

| ***Custodero, et al. Effect of Physical Activity Intervention on Gait Speed by Frailty Condition: A Randomized Clinical Trial. J Am Med Dir Assoc. 2023 Apr;24(4):489-496. doi: 10.1016/j.jamda.2023.01.023. Epub 2023 Mar 3.***  ***https://pubmed.ncbi.nlm.nih.gov/36878264/*** | | | | |
| --- | --- | --- | --- | --- |
| **Study design** | **Study type** | **Country/ Region** | **Clinical setting** | **Duration** |
|  | Secondary analysis of the randomized controlled trial | United States | 8 centers | February 2010 - December 2013 |
|  | **Intervention vs Control** | | **Sample size** | **Female (%)** |
|  | Physical activity vs Health education program | | 1623 | Nonfrail: 66 vs 65.75  Frail: 71.70 vs 73.75 |
|  | **Age, years** | **Conditions** | **Follow-up** | **Loss to follow-up (%)** |
|  | 78.9 (range: 70-89) | older persons with mobility limitations | 24 months | NA. Subset of the original RCT. |
| **Frailty assessment** | **Measurement tool** | **Frailty assessment** | | **Frailty at baseline, Intervention vs Control** |
|  | Other frailty measurement tool | Study of Osteoporotic Fractures frailty index (range: 0-3 criteria)  Nonfrail [0-1 of the 3 criteria] (N=1304)  Frail [2 of the 3 criteria] (N=319) | | Nonfrail: 80.42% vs 80.27%  Frail: 19.58% vs 19.73% |
| **Main findings** | **Outcome** | **Relative effect** | **Analytic approach** | ***P*-value** |
|  | 6-month 400-m gait-speed | Nonfrail: mean difference 0.029 (0.017, 0.041)  Frail: mean difference 0.027 (-0.001, 0.055) | Stratified analysis | NR |
|  | 12-month 400-m gait-speed | Nonfrail: mean difference 0.023 (0.011, 0.035)  Frail: mean difference 0.014 (-0.015, 0.042) |  |  |
|  | 24-month 400-m gait-speed | Nonfrail: mean difference 0.023 (0.010, 0.035)  Frail: mean difference 0.010 (-0.020, 0.039) |  |  |
|  | 6-month 4-m gait-speed | Nonfrail: mean difference -0.004 (-0.018, 0.011)  Frail: mean difference -0.011 (-0.041, 0.018) |  |  |
|  | 12-month 4-m gait-speed | Nonfrail: mean difference -0.002 (-0.016, 0.013)  Frail: mean difference -0.008 (-0.039, 0.012) |  |  |
|  | 24-month 4-m gait-speed | Nonfrail: mean difference -0.001 (-0.014, 0.016)  Frail: mean difference -0.010 (-0.022, 0.41) |  |  |

Abbreviations: NA, not available; NR, not reported.

| ***DiazGranados et al. Efficacy and immunogenicity of high-dose influenza vaccine in older adults by age, comorbidities, and frailty. Vaccine. 2015;33(36):4565-4571. https://pubmed.ncbi.nlm.nih.gov/26187260/*** | | | | |
| --- | --- | --- | --- | --- |
| **Study design** | **Study type** | **Country/ Region** | **Clinical setting** | **Duration** |
|  | Secondary analysis of the randomized controlled trial | United States, Canada | 126 centers | September 6, 2011-May 31, 2013* |
|  | **Intervention vs Control** | | **Sample size** | **Female (%)** |
|  | High-dose inactivated influenza vaccine vs standard-dose vaccine | | 31989 | 56.0-57.0 |
|  | **Age, years** | **Conditions** | **Follow-up** | **Loss to follow-up (%)** |
|  | Mean: 73.3 (range: 65+) | Medically stable olders (without moderate or severe acute illnesses). | 6-8 months | 4.8%* |
| **Frailty assessment** | **Measurement tool** | **Frailty assessment** | | **Frailty at baseline, Intervention vs Control** |
|  | Other frailty measurement tool | Frailty-associated conditions (0-14 conditions): No frailty conditions (N= 4261) One frailty condition (N= 9881) Two frailty conditions (N= 8016) ≥3 frailty conditions (N =9825) | | No frailty conditions: 13.3% vs 13.3% One frailty condition: 31.2% vs 30.6% Two frailty conditions: 24.8 vs 25.3% ≥3 frailty conditions: 30.7% vs 30.8% |
| **Main findings** | **Outcome** | **Relative effect** | **Analytic approach** | ***P*-value for interaction** |
|  | 6-8 month Laboratory-confirmed influenza caused by any viral type/subtype (regardless of similarity to the vaccine) | No frailty conditions: 34.0 [-7.9, 60.2] One frailty condition: 27.5 [0.4, 47.4] Two frailty conditions: 23.9 [-9.0, 47.2] ≥3 frailty conditions: 16.0 [-16.3, 39.4] | Stratified analysis and interaction term | 0.838 |
|  | 6-8 month Culture-confirmed influenza caused by any viral type/subtype (regardless of similarity to the vaccine) | No frailty conditions: 36.5 [-6.3, 62.7] One frailty condition: 24.1 [-6.2, 45.9] Two frailty conditions: 22.0 [-15.0, 47.3] ≥3 frailty conditions: 16.1 [-17.8, 40.4] |  | 0.83 |
|  | 6-8 month Laboratory-confirmed influenza caused by strains similar to the vaccine components | No frailty conditions: 59.0 [7.4, 83.4] One frailty condition: 49.6 [8.5, 73.1] Two frailty conditions: 10.3 [-65.7, 51.8] ≥3 frailty conditions: 22.3 [-36.8, 56.4] |  | 0.28 |
|  | 6-8 month Culture-confirmed influenza caused by strains similar to the vaccine components | No frailty conditions: 59.9 [5.1, 84.7] One frailty condition: 32.1 [-28.7, 64.9] Two frailty conditions: 8.3 [-82.6, 54.3] ≥3 frailty conditions: 26.6 [-37.8, 61.6] |  | 0.463 |

Reference:

*: DiazGranados et al. Efficacy of high-dose versus standard-dose influenza vaccine in older adults. N Engl J Med. 2014;371(7):635-645.

| ***Dimopoulos, et al. Ixazomib as Postinduction Maintenance for Patients With Newly Diagnosed Multiple Myeloma Not Undergoing Autologous Stem Cell Transplantation: The Phase III TOURMALINE-MM4 Trial. J Clin Oncol. 2020;38(34):4030-4041. https://pubmed.ncbi.nlm.nih.gov/33021870/*** | | | | |
| --- | --- | --- | --- | --- |
| **Study design** | **Study type** | **Country/ Region** | **Clinical setting** | **Duration** |
|  | Randomized controlled trial | 34 countries | 187 sites | April 23 2015- October 8 2018 |
|  | **Intervention vs Control** | | **Sample size** | **Female (%)** |
|  | Lxazomib vs placebo | | 706 | 46.6 |
|  | **Age, years** | **Conditions** | **Follow-up** | **Loss to follow-up (%)** |
|  | Mean: 72.5 (range: 18+) | Adults with multiple myeloma | NR | 49.7% |
| **Frailty assessment** | **Measurement tool** | **Frailty assessment** | | **Frailty at baseline, Intervention vs Control** |
|  | Other frailty assessment tool | Fit: (N=284) Unfit: (N=245) Frail: (N=170) | | Fit: 60.5% vs 39.5% Unfit: 60% vs 40% Frail: 60% vs 40% |
| **Main findings** | **Outcome** | **Relative effect** | **Analytic approach** | **p-value for interaction** |
|  | PFS | Fit: HR 0.530 [0.387, 0.727] Unfit: HR 0.746 [0.526, 1.058] Frail: HR 0.733 [0.481, 1.117] | Stratified analysis | NR |
| **Risk of bias** | **Random sequence generation** | **Allocation concealment** | **Blinding of participants and personnel** | **Blinding of outcome assessment** |
|  | Unclear | Low | Low | Low |
|  | **Incomplete outcome data** | **Selective reporting** | **Other bias** |  |
|  | High | Low | Unclear |  |

Abbreviations: NR, not reported; PFS, progression-free survival; HR, hazrad ratio.

Note: Frailty status was classified as fit, unfit, or frail on the basis of four components: age, the Katz Index of Independence in Activities of Daily Living, the Lawton Instrumental Activities of Daily Living Scale, and the Charlson Comorbidity Index Scoring System.

Reference:

Supplement data and protocol available at https://ascopubs.org/doi/suppl/10.1200/JCO.20.02060.

| ***Dolui, et al. Association of Intensive vs Standard Blood Pressure Control With Cerebral Blood Flow: Secondary Analysis of the SPRINT MIND Randomized Clinical Trial. JAMA Neurol. 2022 Apr 1;79(4):380-389. doi: 10.1001/jamaneurol.2022.0074.***  ***https://pubmed.ncbi.nlm.nih.gov/35254390/*** | | | | |
| --- | --- | --- | --- | --- |
| **Study design** | **Study type** | **Country/ Region** | **Clinical setting** | **Duration** |
|  | Secondary analysis of the randomized controlled trial | United States | 6 sites | November 2010 – July 2016 |
|  | **Intervention vs Control** | | **Sample size** | **Female (%)** |
|  | Intensive blood pressure target vs standard blood pressure target | | 547 | 40.04% |
|  | **Age, years** | **Conditions** | **Follow-up** | **Loss to follow-up (%)** |
|  | Mean: 67.9 vs 67.1 (range: 50+) | Adults with hypertension and increased cardiovascular risk but free of diabetes or dementia. | 4 years | NA. Subset of the original RCT. |
| **Frailty assessment** | **Measurement tool** | **Frailty assessment** | | **Frailty at baseline, Intervention vs Control** |
|  | Frailty index | 36-item frailty index (range: 0-1)  Fit [FI≤0.10]  Pre-frail [0.10<FI≤0.21]  Frail [FI>0.21) | | NR |
| **Main findings** | **Outcome** | **Relative effect** | **Analytic approach** | ***P*-value** |
|  | 4-year Whole Brain Cerebral Blood Flow | Fit: difference in change 4.50 (0.02, 8.99)  Pre-frail: difference in change 3.18 (0.49, 5.86)  Frail: difference in change -1.11 (-5.13, 2.90) | Stratified analysis and interaction term | 0.13 |

Abbreviations: NA, not available; NR, not reported.

| ***Ertel et al. Frailty modifies effectiveness of psychosocial intervention in recovery from stroke. Clin Rehabil. 2007;21(6):511-522. https://pubmed.ncbi.nlm.nih.gov/17613582/*** | | | | |
| --- | --- | --- | --- | --- |
| **Study design** | **Study type** | **Country/ Region** | **Clinical setting** | **Duration** |
|  | Randomized controlled trial | United States | Home | NR |
|  | **Intervention vs Control** | | **Sample size** | **Female (%)** |
|  | Psychosocial intervention vs Usual care | | 291 | 48.8 |
|  | **Age, years** | **Conditions** | **Follow-up** | **Loss to follow-up (%)** |
|  | Intervention: 69.3 Control: 70.2 (range: 45+) | Stroke survivors | NR | 8.9%^*^ |
| **Frailty assessment** | **Measurement tool** | **Frailty assessment** | | **Frailty at baseline, Intervention vs Control** |
|  | Frailty index | Summary frailty index (range: 0-5 points): Not frail (3, 5] (N=156)  Frail [0, 3] (N=135) | | NR |
| **Main findings** | **Outcome** | **Absolute effects: mean differences (p-value)** | **Analytic approach** | ***P*-value of interaction** |
|  | 6-month Instrumental ADL | Not frail: 1.11 (p=0.01) Frail: -0.94 (p=0.09) | Stratified analysis and interaction term | ＜0.01 |
|  | 6-month Physical Performance | Not frail: 1.41 (p=0.11) Frail: -1.56 (p=0.10) |  | 0.02 |
|  | 6-month Cognitive Summary | Not frail: 0.09 (p=0.25) Frail: 0.01 (p=0.93) |  | 0.59 |
|  | 47-month Mortality | Not frail: 0.40 (p=0.03) Frail: 1.34 (p=0.27) |  | 0.01 |
|  | 6-month Instrumental efficacy | Not frail: 5.21 (p=0.10) Frail: -11.79 (p=0.01) |  | ＜0.01 |
|  | Recovery efficacy | Not frail: 0.61 (p=0.20) Frail: 0.32 (p=0.61) |  | 0.71 |
|  | Social efficacy | Not frail: 1.69 (p=0.49) Frail: -3.69 (p=0.32) |  | 0.21 |
|  | Received social support | Not frail: -1.05 (p=0.58) Frail: 0.65 (p=0.72) |  | 0.53 |
|  | Social ties index | Not frail: 0.08 (p=0.58) Frail: -0.05 (p=0.80) |  | 0.58 |
| **Risk of bias** | **Random sequence generation** | **Allocation concealment** | **Blinding of participants and personnel** | **Blinding of outcome assessment** |
|  | Low | Low | High | Low |
|  | **Incomplete outcome data** | **Selective reporting** | **Other bias** |  |
|  | Low | Low | Unclear |  |

Abbreviations: NR, not reported; ADL, activities of daily living.

Notes: summary Frailty Index combining measures of physical and mental functioning: depressive symptoms, Mini-Mental State Exam, NIH Stroke Scale, rehabilitation days, and number of pre-existing conditions; Relative effects were not applicable.

Reference:

*: Glass et al. The Families In Recovery From Stroke Trial (FIRST): primary study results. Psychosom Med. 2004;66(6):889-897.

| ***Faber et al. Effects of exercise programs on falls and mobility in frail and pre-frail older adults: A multicenter randomized controlled trial. Arch Phys Med Rehabil. 2006;87(7):885-896. https://pubmed.ncbi.nlm.nih.gov/16813773/*** | | | | |
| --- | --- | --- | --- | --- |
| **Study design** | **Study type** | **Country/ Region** | **Clinical setting** | **Duration** |
|  | Randomized controlled trial | Netherlands | 15 long-term care centers | NR |
|  | **Intervention vs Control** | | **Sample size** | **Female (%)** |
|  | Functional walking vs usual activities; IB vs usual activities | | 278 | 79.0 |
|  | **Age, years** | **Conditions** | **Follow-up** | **Loss to follow-up (%)** |
|  | Mean: 84.9 (range: 63-98) | Participants with varying degrees of frailty. | 52 weeks | 14.4% |
| **Frailty assessment** | **Measurement tool** | **Frailty assessment** | | **Frailty at baseline, Intervention vs Control** |
|  | Frailty phenotype | Fried frailty indicators (range: 0-5 indicators): Not frail [0 of the 5 criteria] (N=15) Pre-frail [1-2 of the 5 criteria] (N=105) Frail [3-5 of the 5 criteria] (N=115) | | Not frail: 6.2% vs 5.0% vs 7.8% Pre-frail: 38.5% vs 41.3% vs 52.2% Frail: 55.4% vs 53.8% vs 40.0% |
| **Main findings** | **Outcome** | **Relative effect** | **Analytic approach** | ***P*-value for interaction** |
|  | 52-weeks Performance Oriented Mobility Assessment (POMA) | Pre-frail: mean 1.1 [0.4, 1.8] Frail: mean 0.5 [-0.6, 1.7] | Stratified analysis and interaction term | not available |
|  | 52-weeks physical performance score (PPS) | Pre-frail: mean 0.7 [0.3, 1.2] Frail: mean -0.7 [-1.3, -0.0] |  | not available |
|  | 52-weeks Groningen Activity Restriction Scale (GARS) | NR |  | not available |
|  | Time to First Fall | Pre-frail HR 0.62 [0.29, 1.33] Frail: HR 2.95 [1.64, 5.32] |  | P=0.002 |
| **Risk of bias** | **Random sequence generation** | **Allocation concealment** | **Blinding of participants and personnel** | **Blinding of outcome assessment** |
|  | Low | Low | Unclear | Unclear |
|  | **Incomplete outcome data** | **Selective reporting** | **Other bias** |  |
|  | Low | Low | Unclear |  |

Abbreviations: NR, not reported; FW, functional walking; IB, in balance; POMA, Performance Oriented Mobility Assessment; PPS, physical performance score; GARS, the Groningen Activity Restriction Scale.

Notes: The analysis based different frailty subgroups so that interaction p value not applicable.

| ***Facon et al. A simplified frailty scale predicts outcomes in transplant-ineligible patients with newly diagnosed multiple myeloma treated in the FIRST (MM-020) trial. Leukemia. 2020;34(1):224-233. https://pubmed.ncbi.nlm.nih.gov/31427722/*** | | | | |
| --- | --- | --- | --- | --- |
| **Study design** | **Study type** | **Country/ Region** | **Clinical setting** | **Duration** |
|  | Secondary analysis of the randomized controlled trial | Europe, North America, and the Asia–Pacific region* | 246 treatment centers* | August 21, 2008-July 14, 2016† |
|  | **Intervention vs Control** | | **Sample size** | **Female (%)** |
|  | continuous lenalidomide and dexamethasone (Rd continuous) vs lenalidomide and dexamethasone for 18 cycles (Rd18) vs fixed-duration melphalan + prednisone + thalidomide (MPT) | | 1618 | 47.4 |
|  | **Age, years** | **Conditions** | **Follow-up** | **Loss to follow-up (%)** |
|  | Median: 73 (range: 40-92)* | Transplant-ineligible patients with newly diagnosed multiple myeloma | Median: 37 months (range: 0-56.7 months)* | NA. Subset of the original RCT. |
| **Frailty assessment** | **Measurement tool** | **Frailty assessment** | | **Frailty at baseline, Intervention vs Control** |
|  | Other frailty measurement tool | Simplified ECOG-based frailty assessment: Non-frail [0,1] (N= 828) Frail [2,7] (N= 790) | | Non-frail: 77% vs 79% Frail: 23% vs 21%* |
| **Main findings** | **Outcome** | **Relative effect** | **Analytic approach** | **p-value for interaction** |
|  | 90-month Progression-Free Survival | Non-frail: HR 0.60 [0.49, 0.75] Frail: HR 0.75 [0.61, 0.91] | Stratified analysis | NR |
|  | 90-month Overall Survival | Non-frail: HR 0.69 [0.54, 0.88] Frail: HR 0.84 [0.68, 1.04] |  |  |

Abbreviations: NR, not reported; NA, not applicable.

Notes: The analysis based different frailty subgroups so that interaction p value not applicable.

Reference:

*: Original RCT. Benboubker et al. Lenalidomide and dexamethasone in transplant-ineligible patients with myeloma. N Engl J Med. 2014;371(10):906-917.

†: Clinical Trial Registration: NCT00689936. Available at https://clinicaltrials.gov/ct2/show/NCT00689936?term=NCT00689936&draw=1&rank=1.

| ***Facon, et al. Daratumumab plus lenalidomide and dexamethasone in transplant-ineligible newly diagnosed multiple myeloma: frailty subgroup analysis of MAIA. Leukemia. 2022 Apr;36(4):1066-1077. doi: 10.1038/s41375-021-01488-8. Epub 2022 Jan 2.***  ***https://pubmed.ncbi.nlm.nih.gov/34974527/*** | | | | |
| --- | --- | --- | --- | --- |
| **Study design** | **Study type** | **Country/ Region** | **Clinical setting** | **Duration** |
|  | Secondary analysis of the randomized controlled trial | 14 countries across North America, Europe, the Middle East, and the Asia–Pacific region* | 176 sites* | March 2015 - January 2017* |
|  | **Intervention vs Control** | | **Sample size** | **Female (%)** |
|  | Daratumumab plus lenalidomide/dexamethasone vs Lenalidomide/dexamethasone | | 737 | Non-frail: 51.0 vs 47.5  Frail: 45.9 vs 46.7 |
|  | **Age, years** | **Conditions** | **Follow-up** | **Loss to follow-up (%)** |
|  | Median:  Non-frail: 71.0 vs 72.0  Frail: 77.0 vs 77.0 | patients with transplant-ineligible newly diagnosed multiple myeloma. | Median: 36.4 months | NA. Subset of the original RCT. |
| **Frailty assessment** | **Measurement tool** | **Frailty assessment** | | **Frailty at baseline, Intervention vs Control** |
|  | Other frailty measurement tool | Non-frail [0 ≤ frailty score≤ 1] (N=396)  Frail [≥ 2] (N=172) † | | Non-frail: 53.3% vs 54.2%  Frail: 46.7% vs 45.8% |
| **Main findings** | **Outcome** | **Relative effect** | **Analytic approach** | ***P*-value** |
|  | 36-month progression-free survival | Non-frail: HR 0.48  Frail: HR 0.62 | Stratified analysis | NR |

Abbreviations: NA, not available; NR, not reported.

*: Facon, et al. Daratumumab plus Lenalidomide and Dexamethasone for Untreated Myeloma. N Engl J Med. 2019 May 30;380(22):2104-2115. doi: 10.1056/NEJMoa1817249. Available at https://pubmed.ncbi.nlm.nih.gov/31141632/.

†: Frailty assessment was performed retrospectively using age, Charlson comorbidity index, and baseline Eastern Cooperative Oncology Group performance status score.

| ***Fairhall et al. Effect of a multifactorial interdisciplinary intervention on mobility-related disability in frail older people: randomised controlled trial. BMC Med. 2012;10:120. https://pubmed.ncbi.nlm.nih.gov/23067364/*** | | | | |
| --- | --- | --- | --- | --- |
| **Study design** | **Study type** | **Country/ Region** | **Clinical setting** | **Duration** |
|  | Randomized controlled trial | Australia* | Community* | Approximately 25 January 2008-2 May 2012† |
|  | **Intervention vs Control** | | **Sample size** | **Female (%)** |
|  | Multifactorial, interdisciplinary intervention vs Usual care* | | 241* | 68.0 |
|  | **Age, years** | **Conditions** | **Follow-up** | **Loss to follow-up (%)** |
|  | Mean: 83.3 (range: 70+)* | People without severe cognitive impairment.* | 1 year* | 10.0% |
| **Frailty assessment** | **Measurement tool** | **Frailty assessment** | | **Frailty at baseline, Intervention vs Control** |
|  | Frailty phenotype* | modified CHS frailty criteria (range: 5 criteria): Less Frail Class 1 [3 of the 5 criteria] (N=156)  More frail Class 2 [4 of the 5 criteria] (N=63)  Class 3 [5 of the 5 criteria] (N=22)* | | Class 1: 64% vs 65% Class 2: 28% vs 25% Class 3: 8% vs 10%* |
| **Main findings** | **Outcome** | **Relative effect** | **Analytic approach** | ***P*-value for interaction** |
|  | 3-month Gait speed | NR | Stratified analysis and interaction term | 0.9 |
|  | 12-month Gait speed | More frail: between-group difference 0.13 [0.04, 0.22] Less frail: between-group difference 0.01 [-0.05, 0.07] |  | 0.03 |
|  | 3-month Life space | More frail: between-group difference 2.0 [-2.8, 6.8] Less frail: between-group difference 9.0 [5.3, 12.7] |  | 0.03 |
|  | 12-month Life space | NR |  | 0.4 |
| **Risk of bias** | **Random sequence generation** | **Allocation concealment** | **Blinding of participants and personnel** | **Blinding of outcome assessment** |
|  | Low | Low | High | Low |
|  | **Incomplete outcome data** | **Selective reporting** | **Other bias** |  |
|  | Low | Low | Low |  |

Abbreviations: NR, not reported.

Reference:

*: Fairhall et al. Effect of a multifactorial, interdisciplinary intervention on risk factors for falls and fall rate in frail older people: a randomised controlled trial. Age Ageing. 2014;43(5):616-622.

†: Clinical Trial Registration: ACTRN12608000250336. Available at https://www.anzctr.org.au/TrialSearch.aspx#&&conditionCode=&dateOfRegistrationFrom=&interventionDescription=&interventionCodeOperator=OR&primarySponsorType=&gender=&distance=&postcode=&pageSize=20&ageGroup=&recruitmentCountryOperator=OR&recruitmentRegion=&ethicsReview=&countryOfRecruitment=&registry=&searchTxt=ACTRN12608000250336&studyType=&allocationToIntervention=&dateOfRegistrationTo=&recruitmentStatus=&interventionCode=&healthCondition=&healthyVolunteers=&page=1&conditionCategory=&fundingSource=&trialStartDateTo=&trialStartDateFrom=&phase=.

| ***Fairhall et al. Effect of a multifactorial, interdisciplinary intervention on risk factors for falls and fall rate in frail older people: a randomised controlled trial. Age Ageing. 2014;43(5):616-622. https://pubmed.ncbi.nlm.nih.gov/24381025/*** | | | | |
| --- | --- | --- | --- | --- |
| **Study design** | **Study type** | **Country/ Region** | **Clinical setting** | **Duration** |
|  | Randomized controlled trial | Australia | Community | Approximately 25 January 2008-2 May 2012* |
|  | **Intervention vs Control** | | **Sample size** | **Female (%)** |
|  | Multifactorial, interdisciplinary intervention vs Usual care | | 241 | 68.0 |
|  | **Age, years** | **Conditions** | **Follow-up** | **Loss to follow-up (%)** |
|  | Mean: 83.3 (range: 70+) | People without severe cognitive impairment | 1 year | 10%† |
| **Frailty assessment** | **Measurement tool** | **Frailty assessment** | | **Frailty at baseline, Intervention vs Control** |
|  | Frailty phenotype | modified CHS frailty criteria (range: 5 criteria): Class 1 [3 criteria] (N=156)  Class 2 [4 criteria] (N=63)  Class 3 [5 criteria] (N=22) | | Class 1: 64% vs 65% Class 2: 28% vs 25% Class 3: 8% vs 10% |
| **Main findings** | **Outcome** | **Relative effect** | **Analytic approach** | **p-value for interaction** |
|  | 3-month Gait speed | NR | Stratified analysis | 0.97 |
|  | 12-month Gait speed | More frail: between-group difference 0.14 [0.06, 0.22] Less frail: between-group difference 0.02 [-0.04, 0.07] |  | 0.03 |
|  | 3- and 12-month SPPB | NR | NR | NR. Just described there was no significant differential effect of the intervention on SPPB or PPA components based on baseline degree of frailty. |
|  | 3- and 12-month PPA | NR | NR |  |
| **Risk of bias** | **Random sequence generation** | **Allocation concealment** | **Blinding of participants and personnel** | **Blinding of outcome assessment** |
|  | Low | Low | High | Low |
|  | **Incomplete outcome data** | **Selective reporting** | **Other bias** |  |
|  | Low | Low | Low |  |

Abbreviations: SPPB, the short physical performance battery; PPA, the Physiological Profile Assessment; NR, not reported.

Reference:

*: Clinical Trial Registration: ACTRN12608000250336. Available at https://www.anzctr.org.au/TrialSearch.aspx#&&conditionCode=&dateOfRegistrationFrom=&interventionDescription=&interventionCodeOperator=OR&primarySponsorType=&gender=&distance=&postcode=&pageSize=20&ageGroup=&recruitmentCountryOperator=OR&recruitmentRegion=&ethicsReview=&countryOfRecruitment=&registry=&searchTxt=ACTRN12608000250336&studyType=&allocationToIntervention=&dateOfRegistrationTo=&recruitmentStatus=&interventionCode=&healthCondition=&healthyVolunteers=&page=1&conditionCategory=&fundingSource=&trialStartDateTo=&trialStartDateFrom=&phase=.

†: Fairhall et al. Effect of a multifactorial interdisciplinary intervention on mobility-related disability in frail older people: randomised controlled trial. BMC Med. 2012;10:120.

| ***Gill et al. A program to prevent functional decline in physically frail, elderly persons who live at home. N Engl J Med. 2002;347(14):1068-1074. https://pubmed.ncbi.nlm.nih.gov/12362007/*** | | | | |
| --- | --- | --- | --- | --- |
| **Study design** | **Study type** | **Country/ Region** | **Clinical setting** | **Duration** |
|  | Randomized controlled trial | United States | General community in greater Bridgeport, Connecticut* | NR |
|  | **Intervention vs Control** | | **Sample size** | **Female (%)** |
|  | Home-based intervention program vs Educational program | | 188 | 79.8 |
|  | **Age, years** | **Conditions** | **Follow-up** | **Loss to follow-up (%)** |
|  | Mean: 83.2 (range: 75-97)* | Physically frail | 1 year | 5.3% |
| **Frailty assessment** | **Measurement tool** | **Frailty assessment** | | **Frailty at baseline, Intervention vs Control** |
|  | Other frailty measurement tools | Two tests of physical abilities: Moderately frail [meeting one of two tests] (N= 116) Severely frail [meeting both tests] (N= 72) | | Moderate: 64% vs 60% Severe: 36% vs 40% |
| **Main findings** | **Outcome** | **Relative effect** | **Analytic approach** | ***P*-value for interaction** |
|  | 1-year disability score (Adjusted) (range: 0-16 points) | Moderate: change 53% Severe: change 16% | Stratified analysis | P<0.001 at 7months and p=0.005 at 12 months. |
| **Risk of bias** | **Random sequence generation** | **Allocation concealment** | **Blinding of participants and personnel** | **Blinding of outcome assessment** |
|  | Low | Low | Unclear | Low |
|  | **Incomplete outcome data** | **Selective reporting** | **Other bias** |  |
|  | Low | Low | Low |  |

Abbreviations: NR, not reported.

Notes: The analysis based different frailty subgroups so that interaction p value not applicable.

Reference:

*: Gill et al. Two recruitment strategies for a clinical trial of physically frail community-living older persons. J Am Geriatr Soc. 2001;49(8):1039-1045.

| ***Gilmore, et al. Evaluating the association of frailty with communication about aging-related concerns between older patients with advanced cancer and their oncologists. Cancer. 2022 Mar 1;128(5):1101-1109. doi: 10.1002/cncr.34010. Epub 2021 Nov 11.***  ***https://pubmed.ncbi.nlm.nih.gov/34762734/*** | | | | |
| --- | --- | --- | --- | --- |
| **Study design** | **Study type** | **Country/ Region** | **Clinical setting** | **Duration** |
|  | Secondary analysis of the randomized controlled trial | US | 30 community oncology practices | October 2014 -April 2017 |
|  | **Intervention vs Control** | | **Sample size** | **Female (%)** |
|  | GA-intervention vs usual-care | | 541 | 48.9 |
|  | **Age, years** | **Conditions** | **Follow-up** | **Loss to follow-up (%)** |
|  | Mean: 76.6(rang: 70-96) | Patients with incurable cancer and impairment on ≥1 GA domain | NR | NA. Subset of the original RCT. |
| **Frailty assessment** | **Measurement tool** | **Frailty assessment** | | **Frailty at baseline, Intervention vs Control** |
|  | Frailty index | Deficit accumulation index (range: 0-1)  Robust [0≤FI<0.2]  Pre-frail [0.2≤FI<0.35]  Frail [≥0.35] | | Mean: 0.31 vs 0.30 |
| **Main findings** | **Outcome** | **Relative effect** | **Analytic approach** | ***P*-value** |
|  | Conversations | Robust: adjusted mean difference 3.27 (1.68, 4.86)  Pre-frail: adjusted mean difference 3.75 (2.32, 5.18)  Frail: adjusted mean difference 4.03 (2.50, 5.56) | Stratified analysis and interaction term | 0.6111 |
|  | Concerns  Acknowledged | Robust: adjusted mean difference 2.08 (1.04, 3.12)  Pre-frail: adjusted mean difference 1.87 (0.97, 2.77)  Frail: adjusted mean difference 2.27 (1.29, 3.25) |  | 0.7397 |
|  | Concerns  Addressed | Robust: adjusted mean difference 2.03 (0.87, 3.19)  Pre-frail: adjusted mean difference 2.13 (1.05, 3.21)  Frail: adjusted mean difference 2.19 (1.07, 3.31) |  | 0.9403 |

Abbreviations: GA, geriatric assessment; NA, not available; NR, not reported.

| ***Gladman, et al. Hospital- and home-based rehabilitation after discharge from hospital for stroke patients: analysis of two trials. Age Ageing. 1995;24(1):49-53. https://pubmed.ncbi.nlm.nih.gov/7762461/*** | | | | |
| --- | --- | --- | --- | --- |
| **Study design** | **Study type** | **Country/ Region** | **Clinical setting** | **Duration** |
|  | Secondary analysis of two RCTs | United Kingdom | Urban | NR |
|  | **Intervention vs Control** | | **Sample size** | **Female (%)** |
|  | Hospital-based treatment vs home-based treatment | | 451 | 31.3 |
|  | **Age, years** | **Conditions** | **Follow-up** | **Loss to follow-up (%)** |
|  | NR. Range: 80+ | Stroke patients | 6 months | NR |
| **Frailty assessment** | **Measurement tool** | **Frailty assessment** | | **Frailty at baseline, Intervention vs Control** |
|  | self-designed Frailty definition | an operational definition of frailty based on (1) age > 80 years, (2) living alone, (3) previous disability, (4) previous Functional Ambulation Category score < 5, (5) not scoring above 6/10 on the AMTS, (6) hospital stay >1 month or discharge Barthel < 15/20: Frailty (≥2 characteristics) | | NR |
| **Main findings** | **Outcome** | **Relative effect** | **Analytic approach** | ***P*-value for interaction** |
|  | 6-month treatment effect (median Barthel score; median improvement in Barthel score; being able to walk outside) | NR | Stratified analysis or interaction term | NR. |

Abbreviations: RCT, randomized controlled trial; NR, not reported;AMTS, a mental test score.

References:

Original RCT-1: Young JB, et al. The Bradford community stroke trial: results at six months. BMJ. 1992;304(6834):1085-1089.

Original RCT-2: Gladman JR, et al. A randomised controlled trial of domiciliary and hospital-based rehabilitation for stroke patients after discharge from hospital. J Neurol Neurosurg Psychiatry. 1993;56(9):960-966.

| ***Gomes, et al. Feasibility, safety, acceptability, and functional outcomes of playing Nintendo Wii Fit PlusTM for frail older adults: A randomized feasibility clinical trial. Maturitas. 2018;118:20-28. https://pubmed.ncbi.nlm.nih.gov/30415751/*** | | | | |
| --- | --- | --- | --- | --- |
| **Study design** | **Study type** | **Country/ Region** | **Clinical setting** | **Duration** |
|  | Randomized controlled trial | Brazil | Geriatric outpatient facility | September 10, 2015-September 10, 2016* |
|  | **Intervention vs Control** | | **Sample size** | **Female (%)** |
|  | Individual training sessions vs education program | | 30 | 93.3 |
|  | **Age, years** | **Conditions** | **Follow-up** | **Loss to follow-up (%)** |
|  | Mean: 84 (range: 71-92) | Frail and pre-frail older adults | 30 days | 13.3% |
| **Frailty assessment** | **Measurement tool** | **Frailty assessment** | | **Frailty at baseline, Intervention vs Control** |
|  | Frailty phenotype | Fried frailty criteria (range: 0-5 criteria): Pre-frail and frail. NSD | | NR |
| **Main findings** | **Outcome** | **Absolute effects** | **Analytic approach** | ***P*-value for interaction** |
|  | Mini-BESTest score at final and follow-up assessments † | Final: Pre-frail: mean 17.6 [14.8, 20.5] vs mean 19.1 [17.4, 20.9] Frail: mean 19.6 [16.6, 22.6] vs mean 15.8 [13.6, 18.0]  Follow-up:  Pre-frail: mean 16.9 [14.1, 19.7] vs mean 19.0 [17.2, 20.8] Frail: mean 17.6 [14.3, 20.9] vs mean 15.7 [13.0, 18.3] | Stratified analysis and interaction term | p>0.05  p>0.05 |
|  | Functional Gait Assessment or FGA at final and follow-up assessments † | Final:  Pre-frail: mean 19.5 [17.2, 21.9] vs mean 18.2 [16.2, 20.3] Frail: mean 18.0 [16.2, 19.8] vs mean 15.8 [13.5, 18.0]  Follow-up: Pre-frail: mean 19.9 [17.4, 22.4] vs mean 19.8 [17.8, 21.7] Frail: mean 19.0 [17.1, 20.9] vs mean 17.6 [15.4, 19.9] |  |  |
| **Risk of bias** | **Random sequence generation** | **Allocation concealment** | **Blinding of participants and personnel** | **Blinding of outcome assessment** |
|  | Unclear | Low | Low | Low |
|  | **Incomplete outcome data** | **Selective reporting** | **Other bias** |  |
|  | Low | Low | Low |  |

Abbreviations: FGA, Functional Gait Assessment; CG1, control group pre-frail; CG2, control group frail; EG1, experimental group pre-frail; EG2, experimental group frail; NR, not reported; NSD, no sufficient data.

Note: Duration form first enrollment to last enrollment.

Reference:

*: Clinical Trial Registration: RBR-823rst. Available at https://ensaiosclinicos.gov.br/rg/RBR-823rst.

†:Data were extracted by GetData Graph Digitizer software.

| ***Guedes de Castro, et al. Survival Outcomes With Short-Course Radiation Therapy in Elderly Patients With Glioblastoma: Data From a Randomized Phase 3 Trial. Int J Radiat Oncol Biol Phys. 2017;98(4):931-938. https://pubmed.ncbi.nlm.nih.gov/28602417/*** | | | | |
| --- | --- | --- | --- | --- |
| **Study design** | **Study type** | **Country/ Region** | **Clinical setting** | **Duration** |
|  | Secondary analysis of the randomized controlled trial | 9 countries | 12 institutions | February 2009- November 2014* |
|  | **Intervention vs Control** | | **Sample size** | **Female (%)** |
|  | 1-week course RT vs 3-week course RT | | 61 | 59.0 |
|  | **Age, years** | **Conditions** | **Follow-up** | **Loss to follow-up (%)** |
|  | NR. Range: 65+ | Patients with glioblastoma. | 2.5 years | NA. Subset of the original RCT. |
| **Frailty assessment** | **Measurement tool** | **Frailty assessment** | | **Frailty at baseline, Intervention vs Control** |
|  | Other frailty assessment tool | KPS (range:50%-100%): Nonfrail [80%, 100%] (N=21) Frail: [50%, 70%] (N= 40) | | Nonfrail: 69%% vs 63% Frail: 31% vs 37% |
| **Main findings** | **Outcome** | **Relative effect** | **Analytic approach** | **p-value for interaction** |
|  | OS | Nonfrail: 8.0 months [5.9, 10.0] vs 8.0 months [5.3, 10.3] Frail: 7.5 months [5.3, 9.7] vs 6.7 months [4.5, 8.9] | Stratified analysis | NR |

Abbreviations: RT, radiation therapy; NR, not reported; NA, not applicable; OS, overall survival.

Note: 9 countries included Belarus, Brazil, Georgia, Greece, India, Ireland, Poland, Thailand, and Tunisia.

References:

*: Clinical Trial Registration: 01450449. Available at https://clinicaltrials.gov/ct2/show/NCT01450449?term=NCT+01450449&draw=2&rank=1.

Original RCT: Roa W, et al. International Atomic Energy Agency Randomized Phase III Study of Radiation Therapy in Elderly and/or Frail Patients With Newly Diagnosed Glioblastoma Multiforme. J Clin Oncol. 2015;33(35):4145-4150.

| ***Jamieson, et al. Deprescribing Anticholinergic and Sedative Drugs to Reduce Polypharmacy in Frail Older Adults Living in the Community: A Randomized Controlled Trial. J Gerontol A Biol Sci Med Sci. 2023 Aug 27;78(9):1692-1700. doi: 10.1093/gerona/glac249.***  ***https://pubmed.ncbi.nlm.nih.gov/36692224/*** | | | | |
| --- | --- | --- | --- | --- |
| **Study design** | **Study type** | **Country/ Region** | **Clinical setting** | **Duration** |
|  | Randomized controlled trial | New Zealand | 2 district health boards | September 25, 2018 -  October 30, 2020 |
|  | **Intervention vs Control** | | **Sample size** | **Female (%)** |
|  | Pharmacist-led deprescribing intervention vs Usual care | | 338 | 66.4% |
|  | **Age, years** | **Conditions** | **Follow-up** | **Loss to follow-up (%)** |
|  | 79.9 (Range: 60-97) | Community-based older adults | 6 months | 6.89% |
| **Frailty assessment** | **Measurement tool** | **Frailty assessment** | | **Frailty at baseline, Intervention vs Control** |
|  | Frailty index | 15-item cumulative deficit model*  Low-frailty (N=117)  Medium-frailty (N=167)  High-frailty (N=79) | | Mean: 0.277 |
| **Main findings** | **Outcome** | **Relative effect** | **Analytic approach** | ***P*-value** |
|  | 6-month changes in DBI 0.5 or more | Low-frailty: difference 9.85% (-3.94%, 23.48%)  Medium-frailty: difference -5.45% (-17.25%, 6.35%)  High-frailty: difference -5.13% (-22.08%, 11.82%)† | Stratified analysis and interaction term | NR |
| **Risk of bias** | **Random sequence generation** | **Allocation concealment** | **Blinding of participants and personnel** | **Blinding of outcome assessment** |
|  | Low | Low | Low | Low |
|  | **Incomplete outcome data** | **Selective reporting** | **Other bias** |  |
|  | Low | Low | Unclear |  |

Abbreviations: DBI, Drug Burden Index; NR, not reported.

*: Bergler, et al. Deprescribing to reduce polypharmacy: study protocol for a randomised controlled trial assessing deprescribing of anticholinergic and sedative drugs in a cohort of frail older people living in the community. Trials. 2021 Nov 3;22(1):766. doi: 10.1186/s13063-021-05711-w.

†: Data were extracted by GetData Graph Digitizer software.

| ***Kaushal, et al. Investigating dose-response effects of multimodal exercise programs on health-related quality of life in older adults. Clin Interv Aging. 2019;14:209-217. https://pubmed.ncbi.nlm.nih.gov/30774322/*** | | | | |
| --- | --- | --- | --- | --- |
| **Study design** | **Study type** | **Country/ Region** | **Clinical setting** | **Duration** |
|  | Secondary analysis of two RCTs | Canada*† | Laboratory | NR |
|  | **Intervention vs Control** | | **Sample size** | **Female (%)** |
|  | lower-dose physical activity vs higher-dose physical activity | | 110 | NR |
|  | **Age, years** | **Conditions** | **Follow-up** | **Loss to follow-up (%)** |
|  | NR. Range: 65+ | Original RCT2: community-dwelling individuals† | 12 weeks | NA. Two RCTs. |
| **Frailty assessment** | **Measurement tool** | **Frailty assessment** | | **Frailty at baseline, Intervention vs Control** |
|  | Other frailty assessment tool | Combined Fried frailty phenotype, modified Physical Performance Test, and 7-point Clinical Frailty Scale^‡^  Frail: meeting at least two of the three methods  NSD | | NR |
| **Main findings** | **Outcome** | **Relative effect** | **Analytic approach** | ***P*-value for interaction** |
|  | 12 weeks HR-QOL | NA | Stratified analysis | 0.037 |

Abbreviations: RCT, randomized controlled trial; NR, not reported; NSD, no sufficient data; HR-QOL, health-related quality of life; NA, not applicable.

"Reference:

*: Original RCT-1: Langlois F, et al. Benefits of physical exercise training on cognition and quality of life in frail older adults. J Gerontol B Psychol Sci Soc Sci. 2013;68(3):400-404.

†: Original RCT-2: Desjardins-Crépeau L, et al. Effects of combined physical and cognitive training on fitness and neuropsychological outcomes in healthy older adults. Clin Interv Aging. 2016;11:1287-1299."

Rockwood K, Song X, MacKnight C, Bergman H, Hogan DB, McDowell I, Mitnitski A. A global clinical measure of fitness and frailty in elderly people. CMAJ. 2005 Aug 30;173(5):489-95. doi: 10.1503/cmaj.050051.

| ***Kitzman, et al. Physical Rehabilitation for Older Patients Hospitalized for Heart Failure. N Engl J Med. 2021;385(3):203-216. https://pubmed.ncbi.nlm.nih.gov/33999544/*** | | | | |
| --- | --- | --- | --- | --- |
| **Study design** | **Study type** | **Country/ Region** | **Clinical setting** | **Duration** |
|  | Randomized controlled trial | the United States* | Hospital | September 2014-July 27, 2020† |
|  | **Intervention vs Control** | | **Sample size** | **Female (%)** |
|  | Rehabilitation intervention vs usual care | | 349 | 52.0 |
|  | **Age, years** | **Conditions** | **Follow-up** | **Loss to follow-up (%)** |
|  | Mean: 72.7 (range: 60+) | Old patients hospitalized with ADHF | 3 months* | 12.9% |
| **Frailty assessment** | **Measurement tool** | **Frailty assessment** | | **Frailty at baseline, Intervention vs Control** |
|  | Frailty phenotype | Modified Fried criteria (range: 0-5 criteria) Frail [≥3 criteria] (N=192) Prefrail [1-2 criteria] (N=145) | | Frail: 53% vs 57% Prefrail: 44% vs 39% |
| **Main findings** | **Outcome** | **Relative effect** | **Analytic approach** | ***P*-value for interaction** |
|  | 3-month Short Physical Performance Battery | Nonfrail or prefrail: 0.7 [-0.1, 1.5] Frail: 2.1 [1.3, 2.8]‡ | Stratified analysis | NR |
| **Risk of bias** | **Random sequence generation** | **Allocation concealment** | **Blinding of participants and personnel** | **Blinding of outcome assessment** |
|  | Unclear | Low | High | Low |
|  | **Incomplete outcome data** | **Selective reporting** | **Other bias** |  |
|  | Low | Low | Low |  |

Abbreviations: ADHF, Acute Decompensated Heart Failure; NR, not reported.

Note: ‡: Between-Group Difference in Mean Score (95% CI).

Reference:

*: Reeves GR, et al. Rehabilitation Therapy in Older Acute Heart Failure Patients (REHAB-HF) trial: Design and rationale. Am Heart J. 2017;185:130-139.

†: Clinical Trial Registration: NCT02196038. Available at https://clinicaltrials.gov/ct2/show/NCT02196038?term=NCT02196038&draw=2&rank=1.

| ***Maclntyre, et al. A randomized clinical trial of the immunogenicity of 7-valent pneumococcal conjugate vaccine compared to 23-valent polysaccharide vaccine in frail, hospitalized elderly. PLoS One. 2014;9(4):e94578. https://pubmed.ncbi.nlm.nih.gov/24760002/*** | | | | |
| --- | --- | --- | --- | --- |
| **Study design** | **Study type** | **Country/ Region** | **Clinical setting** | **Duration** |
|  | Randomized controlled trial | Australia | Hospital | May 2005- December 2007 |
|  | **Intervention vs Control** | | **Sample size** | **Female (%)** |
|  | 23vPPV or PCV7-23vPPV | | 312 | 46.8 |
|  | **Age, years** | **Conditions** | **Follow-up** | **Loss to follow-up (%)** |
|  | Mean: 70 (range: 60+) | Frail and hospitalized elderly | 12 months | 10.30% |
| **Frailty assessment** | **Measurement tool** | **Frailty assessment** | | **Frailty at baseline, Intervention vs Control** |
|  | Frailty index | 40-item frailty index (range: 0-40):  Low [1, 10] (N=182) Moderate [11, 15] (N=80) High [16, 24] (N=50) | | Low: 54.25% vs 62.26% Moderate: 28.76% vs 22.64% High: 16.99% vs 15.09% |
| **Main findings** | **Outcome** | **Absolute effect*,**† | **Analytic approach** | ***P*-value for interaction** |
|  | 12-month ELISA (Serotype 4) (IgG mg/mL) | Low: 1.0 vs 1.5 Moderate/High: 0.8 vs 0.6 | Stratified analysis | NR |
|  | 12-month ELISA (Serotype 18C) (IgG mg/mL) | Low: 5.0 vs 5.4 Moderate/High: 2.9 vs 2.7 |  |  |
|  | 12-month ELISA (Serotype 19F) (IgG mg/mL) | Low: 2.8 vs 3.9 Moderate/High: 2.3 vs 1.8 |  |  |
|  | 12-month OPA (Serotype 4) ( titre-1) | Low: 379.4 vs 454.3 Moderate/High: 224.8 vs 332.6 |  |  |
|  | 72-month OPA (Serotype 18C) ( titre-1) | Low: 336.5 vs 763.0 Moderate/High: 327.0 vs 341.2 |  |  |
|  | 72-month OPA (Serotype 19F) ( titre-1) | Low: 153.7 vs 142.6 Moderate/High: 93.0 vs 93.0 |  |  |
| **Risk of bias** | **Random sequence generation** | **Allocation concealment** | **Blinding of participants and personnel** | **Blinding of outcome assessment** |
|  | Unclear | Low | High | Low |
|  | **Incomplete outcome data** | **Selective reporting** | **Other bias** |  |
|  | Low | Low | Low |  |

Abbreviations: 23vPPV, 23-valent capsular polysaccharide; PCV7, 7-valent conjugate.

Note: The relative effects were not reported. †: Data were extracted by GetData Graph Digitizer software.

References:

Clinical Trial Registration: ACTRN12607000387426. Available at https://www.anzctr.org.au/TrialSearch.aspx#&&conditionCode=&dateOfRegistrationFrom=&interventionDescription=&interventionCodeOperator=OR&primarySponsorType=&gender=&distance=&postcode=&pageSize=20&ageGroup=&recruitmentCountryOperator=OR&recruitmentRegion=&ethicsReview=&countryOfRecruitment=&registry=&searchTxt=+ACTRN12607000387426&studyType=&allocationToIntervention=&dateOfRegistrationTo=&recruitmentStatus=&interventionCode=&healthCondition=&healthyVolunteers=&page=1&conditionCategory=&fundingSource=&trialStartDateTo=&trialStartDateFrom=&phase=.

*: Data were extracted by GetData Graph Digitizer software.

| ***Maclntyre, et al. Persistence of immunity to conjugate and polysaccharide pneumococcal vaccines in frail, hospitalised older adults in long-term follow up. Vaccine. 2019;37(35):5016-5024. https://pubmed.ncbi.nlm.nih.gov/31300288/*** | | | | |
| --- | --- | --- | --- | --- |
| **Study design** | **Study type** | **Country/ Region** | **Clinical setting** | **Duration** |
|  | Randomized controlled trial | Australia | Hospital | May 2005- February 2008 |
|  | **Intervention vs Control** | | **Sample size** | **Female (%)** |
|  | 23vPPV or PCV7-23vPPV | | 312 | 46.8 |
|  | **Age, years** | **Conditions** | **Follow-up** | **Loss to follow-up (%)** |
|  | Mean: 71 (range: 60+) | Frail and hospitalized elderly. | 6 years | 56.4% |
| **Frailty assessment** | **Measurement tool** | **Frailty assessment** | | **Frailty at baseline, Intervention vs Control** |
|  | Frailty index | 40-item frailty index (range: 0-1):  Low [0,10] (N= 97) Moderate [11, 15] (N=25) High frailty [16, 24] (N= 14) | | Low: 75.8% vs 67.6% Moderate: 12.9% vs 23.0% High: 11.3% vs 9.5% |
| **Main findings** | **Outcome** | **Absolute effect*,†** | **Analytic approach** | ***P*-value for interaction** |
|  | 72-month ELISA (Serotype 18C) (IgG mg/mL) | Low: 6.6 vs 5.9 Moderate/High: 5.5 vs 2.3 | Stratified analysis | NR |
|  | 72-month ELISA (Serotype 4) (IgG mg/mL) | Low: 1.6 vs 1.7 Moderate/High: 1.2 vs 0.6 |  |  |
|  | 72-month OPA (Serotype 18C) ( titre-1) | Low: 244.1 vs 310.4 Moderate/High: 299.1 vs 62.1 |  |  |
|  | 72-month OPA (Serotype 23F) ( titre-1) | Low: 38.9 vs 76.8 Moderate/High: 30.2 vs 19.6 |  |  |
| **Risk of bias** | **Random sequence generation** | **Allocation concealment** | **Blinding of participants and personnel** | **Blinding of outcome assessment** |
|  | Unclear | Low | High | Low |
|  | **Incomplete outcome data** | **Selective reporting** | **Other bias** |  |
|  | Low | Low | Low |  |

Abbreviations: 23vPPV, 23-valent capsular polysaccharide; PCV7, 7-valent conjugate; ELISA, enzyme-linked immunosorbent assay; OPA, opsonophagocytic assays.

Note: The relative effects were not reported. †: Data were extracted by GetData Graph Digitizer software.

References:

Clinical Trial Registration: ACTRN12613001244796. Available at https://www.anzctr.org.au/TrialSearch.aspx#&&conditionCode=&dateOfRegistrationFrom=&interventionDescription=&interventionCodeOperator=OR&primarySponsorType=&gender=&distance=&postcode=&pageSize=20&ageGroup=&recruitmentCountryOperator=OR&recruitmentRegion=&ethicsReview=&countryOfRecruitment=&registry=&searchTxt=ACTRN12613001244796&studyType=&allocationToIntervention=&dateOfRegistrationTo=&recruitmentStatus=&interventionCode=&healthCondition=&healthyVolunteers=&page=1&conditionCategory=&fundingSource=&trialStartDateTo=&trialStartDateFrom=&phase=.

*: Data were extracted by GetData Graph Digitizer software.

| ***Magarotto et al. Triplet vs doublet lenalidomide-containing regimens for the treatment of elderly patients with newly diagnosed multiple myeloma. Blood. 2016;127(9):1102-1108. https://pubmed.ncbi.nlm.nih.gov/26729895/*** | | | | |
| --- | --- | --- | --- | --- |
| **Study design** | **Study type** | **Country/ Region** | **Clinical setting** | **Duration** |
|  | Randomized controlled trial | Italian and Czech Republic | 67 centers | October 2009-November 2022* |
|  | **Intervention vs Control** | | **Sample size** | **Female (%)** |
|  | MPR vs CPR vs RD | | 654 | 51.4 |
|  | **Age, years** | **Conditions** | **Follow-up** | **Loss to follow-up (%)** |
|  | Median: 73 (range: 65+)† | Newly diagnosed MM patients | Median: 39 months | 38.5% |
| **Frailty assessment** | **Measurement tool** | **Frailty assessment** | | **Frailty at baseline, Intervention vs Control** |
|  | Other frailty assessment tool | International Myeloma Working Group geriatric score (Frailty Score) (range: 0-5 scores)‡ Fit [0] (N=285) Intermediate-fitness [1] (N=206) Frail [≥2] (N=168) | | MPR vs CPR vs Rd: Fit: 41% vs 44% vs 45% Intermediate fitness: 36% vs 32% 26% Frail: 23% vs 24% vs 28% |
| **Main findings** | **Outcome** | **Relative effect** | **Analytic approach** | ***P*-value for interaction** |
|  | 60-month PFS | MPR + CPR vs Rd: Fit: HR 0.793 [0.584, 1.077] Intermediate-fitness: 0.868 [0.591, 1.274] Frail: HR 1.223 [0.824, 1.817] MPR vs Rd: Fit: HR 0.671 [0.461, 0.976] Intermediate-fitness: HR 0.850 [0.550, 1.313] Frail: HR 1.030 [0.632, 1.680] CPR vs Rd: Fit: HR 0.957 [0.805, 1.139] Intermediate-fitness: HR 0.944 [0.758, 1.175] Frail: HR 1.180 [0.947, 1.470] MPR vs CPR: Fit: HR 0.740 [0.506, 1.082] Intermediate-fitness: HR 0.966 [0.637, 1.466] Frail: HR 0.731 [0.452, 1.182] | Stratified analysis | NA |
| **Risk of bias** | **Random sequence generation** | **Allocation concealment** | **Blinding of participants and personnel** | **Blinding of outcome assessment** |
|  | Low | Low | High | High |
|  | **Incomplete outcome data** | **Selective reporting** | **Other bias** |  |
|  | High | Low | Low |  |

Abbreviations: MPR, melphalan-prednisone-lenalidomide; CPR, cyclophosphamide-prednisone-lenalidomide; RD, lenalidomide plus low-dose dexamethasone; MM, multiple myeloma; PFS, progression-free survival; NA, not applicable.

Notes: Duration was study start date to estimated study completion date. The analysis based different frailty subgroups so that interaction p value not applicable.

Reference:

*: Clinical Trial Registration: NCT01093196. Avaliable at https://clinicaltrials.gov/ct2/show/NCT01093196?term=NCT01093196&draw=2&rank=1.

†: Bringhen et al. Lenalidomide-based induction and maintenance in elderly newly diagnosed multiple myeloma patients: updated results of the EMN01 randomized trial. Haematologica. 2020;105(7):1937-1947.

‡: Supplement data. Available at https://ashpublications.org/blood/article/127/9/1102/126446/Triplet-vs-doublet-lenalidomide-containing.

| ***Metzelthin et al. Effectiveness of interdisciplinary primary care approach to reduce disability in community dwelling frail older people: cluster randomised controlled trial. BMJ. 2013;347:f5264. Published 2013 Sep 10. https://pubmed.ncbi.nlm.nih.gov/24022033/*** | | | | |
| --- | --- | --- | --- | --- |
| **Study design** | **Study type** | **Country/ Region** | **Clinical setting** | **Duration** |
|  | Randomized controlled trial | Netherlands | 12 general practices | April 11, 2009-June 30, 2012* |
|  | **Intervention vs Control** | | **Sample size** | **Female (%)** |
|  | Prevention of care approach vs usual care | | 346 | 57.5 |
|  | **Age, years** | **Conditions** | **Follow-up** | **Loss to follow-up (%)** |
|  | Mean: 77.2 (range: 70+) | Frail older people | 24 months | 22.0% |
| **Frailty assessment** | **Measurement tool** | **Frailty assessment** | | **Frailty at baseline, Intervention vs Control** |
|  | Other frailty assessment tool | Groningen Frailty Indicator (range: 0-15) Low frailty [5,6] High frailty [7,14] NSD | | GFI 6.72 vs 7.13 |
| **Main findings** | **Outcome** | **Relative effect** | **Analytic approach** | **p-value for interaction** |
|  | Groningen Activity Restriction Scale (range total scale 18-78) at 6, 12, and 24 months | NR | Interaction term | ＞0.05 |
| **Risk of bias** | **Random sequence generation** | **Allocation concealment** | **Blinding of participants and personnel** | **Blinding of outcome assessment** |
|  | Low | Low | High | Low |
|  | **Incomplete outcome data** | **Selective reporting** | **Other bias** |  |
|  | High | Low | Unclear |  |

Abbreviations: NSD, no sufficient data; NR, not reported.

Reference:

*: Clinical Trial Registration: ISRCTN31954692. Available at https://www.isrctn.com/ISRCTN31954692?q=ISRCTN31954692&filters=&sort=&offset=1&totalResults=1&page=1&pageSize=10&searchType=basic-search.

| ***Newman, et al. Cardiovascular Events in a Physical Activity Intervention Compared With a Successful Aging Intervention: The LIFE Study Randomized Trial. JAMA Cardiol. 2016;1(5):568-574. https://www.ncbi.nlm.nih.gov/pmc/articles/PMC5755709/*** | | | | |
| --- | --- | --- | --- | --- |
| **Study design** | **Study type** | **Country/ Region** | **Clinical setting** | **Duration** |
|  | Randomized controlled trial | United States | 8 centers | April 2004-January 2006* |
|  | **Intervention vs Control** | | **Sample size** | **Female (%)** |
|  | Physical activity vs successful aging | | 1635 | 67.0 |
|  | **Age, years** | **Conditions** | **Follow-up** | **Loss to follow-up (%)** |
|  | Mean: 78.7 (range: 70-89) | With a Short Physical Performance Battery (SPPB) score of 9 or less but able to walk 400 m. | 3.5 years | 2.3%† |
| **Frailty assessment** | **Measurement tool** | **Frailty assessment** | | **Frailty at baseline, Intervention vs Control** |
|  | Other frailty assessment tool | SPPB (range: 0-12 score) SPPB ＜8 (N=731) SPPB 8 or 9 (N=904) | | SPPB ＜8: 43.2% vs 46.3% SPPB 8 or 9: 56.8% vs 53.7% |
| **Main findings** | **Outcome** | **Relative effect** | **Analytic approach** | ***P*-value for interaction** |
|  | 3.5-year total cardiovascular disease event rates | SPPB ＜8: HR 0.76 [0.52, 1.10] SPPB 8 or 9: HR 1.59 [1.09, 2.30] | Stratified analysis and interaction term | 0.006 |
| **Risk of bias** | **Random sequence generation** | **Allocation concealment** | **Blinding of participants and personnel** | **Blinding of outcome assessment** |
|  | Low | Low | High | Low |
|  | **Incomplete outcome data** | **Selective reporting** | **Other bias** |  |
|  | Low | Low | Low |  |

Abbreviations: SPPB, Short Physical Performance Battery; NR, not reported

Reference:

*: Clinical Trial Registration: NCT00116194. Available at https://clinicaltrials.gov/ct2/show/NCT00116194?term=NCT00116194&draw=2&rank=1.

†: Pahor M, et al. Effect of structured physical activity on prevention of major mobility disability in older adults: the LIFE study randomized clinical trial. JAMA. 2014;311(23):2387-2396. doi:10.1001/jama.2014.5616

| ***Nishtala, et al. Post Hoc Analyses of a Randomized Controlled Trial for the Effect of Pharmacist Deprescribing Intervention on the Anticholinergic Burden in Frail Community-Dwelling Older Adults. J Am Med Dir Assoc. 2023 Aug;24(8):1253-1260. doi: 10.1016/j.jamda.2023.05.014. Epub 2023 Jun 17.***  ***https://pubmed.ncbi.nlm.nih.gov/37339754/*** | | | | |
| --- | --- | --- | --- | --- |
| **Study design** | **Study type** | **Country/ Region** | **Clinical setting** | **Duration** |
|  | Secondary analysis of the randomized controlled trial | New Zealand | 2 district health boards | September 25, 2018 -  October 30, 2020 |
|  | **Intervention vs Control** | | **Sample size** | **Female (%)** |
|  | Pharmacist-led deprescribing intervention vs Usual care | | 295 | 67% |
|  | **Age, years** | **Conditions** | **Follow-up** | **Loss to follow-up (%)** |
|  | Median: 79 | Older adults | 6 months | NA |
| **Frailty assessment** | **Measurement tool** | **Frailty assessment** | | **Frailty at baseline, Intervention vs Control** |
|  | Frailty index | 15-item cumulative deficit model*  Low-frailty (N=88)  Medium-frailty (N=142)  High-frailty (N=65) | | 0.27 vs 0.28 |
| **Main findings** | **Outcome** | **Relative effect** | **Analytic approach** | ***P*-value** |
|  | ACB | Low-frailty: mean change -0.02 (-0.65, 0.18)  Medium-frailty: mean change 0.05 (-0.28, 0.38)  High-frailty: mean change 0.08 (-0.40, 0.56) | Stratified analysis | NR |

Abbreviations: ACB, anticholinergic cognitive burden; NA, not available; NR, not reported.

*: Bergler, et al. Deprescribing to reduce polypharmacy: study protocol for a randomised controlled trial assessing deprescribing of anticholinergic and sedative drugs in a cohort of frail older people living in the community. Trials. 2021 Nov 3;22(1):766. doi: 10.1186/s13063-021-05711-w.

| ***Orkaby, et al. Effect of canakinumab on frailty: A post hoc analysis of the CANTOS trial. Aging Cell. 2023 Nov 5:e14029. doi: 10.1111/acel.14029. Epub ahead of print.***  ***https://pubmed.ncbi.nlm.nih.gov/37927208/*** | | | | |
| --- | --- | --- | --- | --- |
| **Study design** | **Study type** | **Country/ Region** | **Clinical setting** | **Duration** |
|  | Secondary analysis of the randomized controlled trial | 39 countries | 1091 clinical sites | 2011 - 2017 |
|  | **Intervention vs Control** | | **Sample size** | **Female (%)** |
|  | Canakinumab (50, 100, or 300 mg) vs  Placebo | | 9942 | 25.6% |
|  | **Age, years** | **Conditions** | **Follow-up** | **Loss to follow-up (%)** |
|  | Median: 61 | Stable post-myocardial infarction patients | 60 months | NA |
| **Frailty assessment** | **Measurement tool** | **Frailty assessment** | | **Frailty at baseline, Intervention vs Control** |
|  | Frailty index | 34-item cumulative-deficit Frailty  Index (range: 0-1)  Non frail [FI <0.1] (N=4052)  Pre-frail [0.1≤ FI <0.2] (N=4540)  Frail [FI ≥0.2] (N=1350) | | Median: 0.12 |
| **Main findings** | **Outcome** | **Relative effect** | **Analytic approach** | ***P*-value** |
|  | 60-month incident MACE | Not frail: HR 0.89 (0.73, 1.10)  Prefrail: HR 0.86 (0.74, 1.00)  Frail: HR 0.87 (0.71, 1.08) | Stratified analysis | NR |

Abbreviations: MACE, major adverse cardiovascular events; NA, not available; NR, not reported.

| ***Pandey et al. Frailty and Effects of a Multidomain Physical Rehabilitation Intervention Among Older Patients Hospitalized for Acute Heart Failure: A Secondary Analysis of a Randomized Clinical Trial. JAMA Cardiol. 2023 Feb 1;8(2):167-176. doi: 10.1001/jamacardio.2022.4903. https://pubmed.ncbi.nlm.nih.gov/36598761/*** | | | | |
| --- | --- | --- | --- | --- |
| **Study design** | **Study type** | **Country/ Region** | **Clinical setting** | **Duration** |
|  | Secondary analysis of the randomized controlled trial | United States | NR | September 17 2014 - September 19 2019 |
|  | **Intervention vs Control** | | **Sample size** | **Female (%)** |
|  | Physical rehabilitation intervention vs attention control | | 337 | 53.7 |
|  | **Age, years** | **Conditions** | **Follow-up** | **Loss to follow-up (%)** |
|  | Mean: 72 (range: 60+) | Patients 60 years and older hospitalized for acute decompensated heart failure (ADHF) | 3 months | 3.44% |
| **Frailty assessment** | **Measurement tool** | **Frailty assessment** | | **Frailty at baseline, Intervention vs Control** |
|  | Frailty phenotype | Modified Fried criteria: Prefrail [1-2 of the 5 criteria] (N=145) Frail [≥ 3 criteria] (N=192) | | Prefrail: 45.56% vs 40.48% Frail: 54.44% vs 59.52% |
| **Main findings** | **Outcome** | **Relative effect** | **Analytic approach** | ***P*-value** |
|  | 3-month Short Physical Performance Battery (SPPB) score | Prefrail: Effect size 0.8 (–0.1, 1.6) Frail: Effect size 2.1 (1.3, 2.9) | Interaction term | P for interaction = 0.03 |

Abbreviations: NR, not reported.

| ***Pandey, et al. Frailty Status Modifies the Efficacy of Exercise Training Among Patients With Chronic Heart Failure and Reduced Ejection Fraction: An Analysis From the HF-ACTION Trial. Circulation. 2022 Jul 12;146(2):80-90. doi: 10.1161/CIRCULATIONAHA.122.059983. Epub 2022 May 26.***  ***https://pubmed.ncbi.nlm.nih.gov/35616018/*** | | | | |
| --- | --- | --- | --- | --- |
| **Study design** | **Study type** | **Country/ Region** | **Clinical setting** | **Duration** |
|  | Secondary analysis of the randomized controlled trial | United States, Canada, and France* | 82 centers* | 2002 - 2007 |
|  | **Intervention vs Control** | | **Sample size** | **Female (%)** |
|  | Aerobic exercise training vs Usual care | | 2130 | 28% |
|  | **Age, years** | **Conditions** | **Follow-up** | **Loss to follow-up (%)** |
|  | Mean: 59 | Stable patients with HFrEF | 36 months | NA |
| **Frailty assessment** | **Measurement tool** | **Frailty assessment** | | **Frailty at baseline, Intervention vs Control** |
|  | Frailty index | 36-item frailty index (Rockwood’s Frailty index, range: 0-1)  Non-frail [FI ≤ 0.21] (N=864)  Frail [FI > 0.21] (N=1266) | | NR |
| **Main findings** | **Outcome** | **Relative effect** | **Analytic approach** | ***P*-value** |
|  | 36-month composite of all-cause hospitalization or all-cause mortality | Non-frail: HR 1.04 (0.87, 1.25)  Frail: HR 0.83 (0.72, 0.95) | Stratified analysis and interaction term | NR |

Abbreviations: HFrEF, heart failure with reduced ejection fraction; NA, not available; NR, not reported.

*: O'Connor, et al. Efficacy and safety of exercise training in patients with chronic heart failure: HF-ACTION randomized controlled trial. JAMA. 2009 Apr 8;301(14):1439-50. doi: 10.1001/jama.2009.454. Available at https://pubmed.ncbi.nlm.nih.gov/19351941/.

| ***Park, et al. Frailty modifies the intervention effect of chair yoga on pain among older adults with lower extremity osteoarthritis: Secondary analysis of a nonpharmacological intervention trial [published online ahead of print, 2020 Feb 20]. Exp Gerontol. 2020;134:110886. https://pubmed.ncbi.nlm.nih.gov/32088398/*** | | | | |
| --- | --- | --- | --- | --- |
| **Study design** | **Study type** | **Country/ Region** | **Clinical setting** | **Duration** |
|  | Secondary analysis of the randomized controlled trial | United States | Community sites | March 2014-September 2016* |
|  | **Intervention vs Control** | | **Sample size** | **Female (%)** |
|  | Chair Yoga vs health education | | 112 | 75.9 |
|  | **Age, years** | **Conditions** | **Follow-up** | **Loss to follow-up (%)** |
|  | Mean: 75.3 (range: 65+) | With lower extremity osteoarthritis. | 3 months | 14.5% |
| **Frailty assessment** | **Measurement tool** | **Frailty assessment** | | **Frailty at baseline, Intervention vs Control** |
|  | Frailty index | 82-item Frailty index (range: 0-1 points) | | both FI mean 0.43 |
| **Main findings** | **Outcome** | **Relative effect** | **Analytic approach** | **p-value for interaction** |
|  | 8-weeks WOMAC pain | Figures 2 and 3. At lower level of baseline frailty (mean FI=0.39), slight declines in WOMAC pain and pain interference in both intervention and control groups, with no substantial difference in rates of declines.   At higher level of baseline frailty (mean FI=0.57), slight declines in WOMAC pain and pain interference in the intervention group but rapid increases in the control group | Stratified analysis and interaction term | 0.02 |
|  | 8-weeks pain interference |  |  | 0.01 |

Abbreviations: FI, frailty index; WOMAC, Western Ontario and McMaster Universities.

Reference:

*: Clinical Trial Registration: NCT02113410. Available at https://clinicaltrials.gov/ct2/show/NCT02113410?term=NCT02113410&draw=2&rank=1.

| ***Pulignano et al. Usefulness of frailty profile for targeting older heart failure patients in disease management programs: a cost-effectiveness, pilot study. J Cardiovasc Med (Hagerstown). 2010;11(10):739-747. https://pubmed.ncbi.nlm.nih.gov/20736784/*** | | | | |
| --- | --- | --- | --- | --- |
| **Study design** | **Study type** | **Country/ Region** | **Clinical setting** | **Duration** |
|  | Secondary analysis of the randomized controlled trail | United States | Heart failure outpatient clinic | NR |
|  | **Intervention vs Control** | | **Sample size** | **Female (%)** |
|  | Disease management programs vs usual care | | 173 | 48.0 |
|  | **Age, years** | **Conditions** | **Follow-up** | **Loss to follow-up (%)** |
|  | Intervention: 77.4 Control: 77.5  (range: 70+) | Olders with reduced and normal left ventricular ejection fraction and discharged home | 2 years | NA. Subset of the original RCT. |
| **Frailty assessment** | **Measurement tool** | **Frailty assessment** | | **Frailty at baseline, Intervention vs Control** |
|  | Other frailty measurement tool | Modified frailty score (range: 1-6 score):  Frailty score 1 (N= 37)  Frailty score 2 (N= 71) Frailty score 3 (N= 37) Frailty score 4-6 (N= 28) | | Frailty score 1: 19.8% vs 23.0% Frailty score 2: 38.4% vs 43.7% Frailty score 3: 26.7% vs 16.1% Frailty score 4-6: 15.1% vs 17.2% |
| **Main findings** | **Outcome** | **Relative effect** | **Analytic approach** | **p-value for interaction** |
|  | 2-year Death and/or heart failure admissions | Frailty score 1: HR 2.506 [0.459, 13.687] Frailty score 2: HR 0.441 [0.226, 0.859] Frailty score 3: HR 0.426 [0.195, 0.932] Frailty score 4-6: HR 0.369 [0.153, 0.891] | Stratified analysis and interaction | 0.208 |
|  | 2-year All-cause admissions | Frailty score 1: HR 1.905 [0.723, 5.024] Frailty score 2: HR 0.490 [0.268, 5.894] Frailty score 3: HR 0.285 [0.124, 0.650] Frailty score 4-6: HR 0.467 [0.200, 1.089] |  | 0.0178 |
|  | 2-year Heart failure admissions | Frailty score 1: NR Frailty score 2: HR 0.485 [0.239, 0.981] Frailty score 3: HR 0.207 [0.078, 0.550] Frailty score 4-6: HR 0.307 [0.116, 0.812] |  | 0.38 |

Abbreviations: NR, not reported; NA, not applicable; RCT, randomized controlled trial; HR, hazard ratio.

| ***Pérez-Zepeda, et al. The impact of an exercise intervention on frailty levels in hospitalised older adults: secondary analysis of a randomised controlled trial. Age Ageing. 2022 Feb 2;51(2):afac028. doi: 10.1093/ageing/afac028.***  ***https://pubmed.ncbi.nlm.nih.gov/35180287/*** | | | | |
| --- | --- | --- | --- | --- |
| **Study design** | **Study type** | **Country/ Region** | **Clinical setting** | **Duration** |
|  | Secondary analysis of the randomized controlled trial | Spain* | Hospital | February 2015 - August 2017 |
|  | **Intervention vs Control** | | **Sample size** | **Female (%)** |
|  | Intensive exercise intervention vs Usual care | | 323 | 56.3 |
|  | **Age, years** | **Conditions** | **Follow-up** | **Loss to follow-up (%)** |
|  | Mean: 87.1 (range: 75+) | Adults admitted to an acute care ward | 58 months | NA |
| **Frailty assessment** | **Measurement tool** | **Frailty assessment** | | **Frailty at baseline, Intervention vs Control** |
|  | Frailty index | 63-item Frailty Index (range: 0-1)  Class 1 [FI < 0.2]  Class 2 [0.2 ≤ FI < 0.29]  Class 3 [FI ≥ 0.3] | | Mean: 0.26 vs 0.25 |
| **Main findings** | **Outcome** | **Relative effect** | **Analytic approach** | ***P*-value** |
|  | Frailty Index | Class 1: OR 0.16 (0.01, 1.45)  Class 2: OR 0.08 (0.01, 25.50)  Class 3: OR 0.01 (0.01, 0.37) | Stratified analysis | NR |
|  | Barthel Index | Class 1: OR 2.60 (0.20,243.40)  Class 2: OR 0.19 (0.04, 0.91)  Class 3: OR 0.12 (0.01, 0.78) |  |  |
|  | Short Physical Performance Battery | Class 1: OR 0.13 (0.01, 1.72)  Class 2: OR 0.15 (0.02, 1.12)  Class 3: OR 0.13 (0.01, 1.61) |  |  |
|  | Mini-Mental State Examination | Class 1: beta coefficient -1.15 (-2.5, 0.19)  Class 2: beta coefficient -1.4 (-3.03, 0.223)  Class 3: beta coefficient -0.97 (-2.67, 0.71) |  |  |
|  | Geriatric Depression Scale | Class 1: beta coefficient -1.1 (-2.3, -0.02)  Class 2: beta coefficient -2 (-3.2, -0.7)  Class 3: beta coefficient -2 (-3.4, -0.7) |  |  |
|  | EuroQoL 5D visual analogue scale | Class 1: beta coefficient -2.7 (-17, 11.5)  Class 2: beta coefficient 9.4 (-4.6, 23.5)  Class 3: beta coefficient 18.6 (2.8, 34.5) |  |  |
|  | Handgrip strength | Class 1: beta coefficient -9.3 (-29.8, 11.1)  Class 2: beta coefficient -4.4 (-20.8, 11.9)  Class 3: beta coefficient 14.6 (-5.7, 34.9) |  |  |
|  | Incident delirium | Class 1: OR 1.4 (0.2, 111.7)  Class 2: OR 5.4 (0.4, 67.8)  Class 3: OR 1.1 (0.2, 7.5) |  |  |
|  | In-hospital falls | Class 1: OR 0.18 (0.01, 5.8)  Class 2: OR 0.9 (0.07, 12.3)  Class 3: OR 8.7 (0.24, 316.5) |  |  |
|  | Readmission | Class 1: OR 2.2 (0.28, 17.4)  Class 2: OR 0.3 (0.05, 1.7)  Class 3: OR 0.7 (0.1, 4.5) |  |  |
|  | Place of discharge not home | Class 1: OR 1.5 (0.03, 77.7)  Class 2: OR 2 (0.21, 19)  Class 3: OR 0.3 (0.03, 3.8) |  |  |
|  | Visited emergency room after discharge | Class 1: OR 3.1 (0.51, 19.3)  Class 2: OR 0.3 (0.06, 1.6)  Class 3: OR 0.2 (0.03, 1.7) |  |  |
|  | 58-month Mortality (extended follow-up) | Class 1: HR 0.9 (0.18, 4.3)  Class 2: HR 0.4 (0.08, 2.6)  Class 3: HR 0.8 (0.2, 2.9) |  |  |

Abbreviations: NA, not available; NR, not reported.

*: Martínez-Velilla, et al. Effect of Exercise Intervention on Functional Decline in Very Elderly Patients During Acute Hospitalization: A Randomized Clinical Trial. JAMA Intern Med. 2019 Jan 1;179(1):28-36. doi: 10.1001/jamainternmed.2018.4869. Erratum in: JAMA Intern Med. 2019 Jan 1;179(1):127. Available at https://pubmed.ncbi.nlm.nih.gov/30419096/.

| ***Quach, et al. Effect of a physical activity intervention and frailty on frailty trajectory and major mobility disability. J Am Geriatr Soc. 2022 Oct;70(10):2915-2924. doi: 10.1111/jgs.17941. Epub 2022 Jul 2.***  ***https://pubmed.ncbi.nlm.nih.gov/35779276/*** | | | | |
| --- | --- | --- | --- | --- |
| **Study design** | **Study type** | **Country/ Region** | **Clinical setting** | **Duration** |
|  | Secondary analysis of the randomized controlled trial | United States | 8 centers | February 2010 – December 2013 |
|  | **Intervention vs Control** | | **Sample size** | **Female (%)** |
|  | Physical activity vs Health education | | 1635 | 67.2 |
|  | **Age, years** | **Conditions** | **Follow-up** | **Loss to follow-up (%)** |
|  | Mean: 78.9 (range: 70-89) | Community-dwelling participants | 2 years | NA |
| **Frailty assessment** | **Measurement tool** | **Frailty assessment** | | **Frailty at baseline, Intervention vs Control** |
|  | Frailty index | 44-item frailty index | | Mean: 0.18 |
| **Main findings** | **Outcome** | **Relative effect** | **Analytic approach** | ***P*-value** |
|  | 2-year major mobility disability | Centered FI 0.05: HR 0.87 (0.64, 1.17)  Centered FI 0.10: HR 0.84 (0.66, 1.06)  Centered FI 0.15: HR 0.81 (0.67, 0.98)  Centered FI 0.20: HR 0.78 (0.66, 0.92)  Centered FI 0.25: HR 0.75 (0.62, 0.90)  Centered FI 0.30: HR 0.72 (0.58, 0.90)  Centered FI 0.35: HR 0.70 (0.53, 0.92)  Centered FI 0.40: HR 0.67 (0.47, 0.95)  Centered FI 0.45: HR 0.65 (0.43, 0.98) | Stratified analysis | NR |

Abbreviations: NA, not available; NR, not reported.

| ***Rodriguez-Mañas, et al. Effectiveness of a multimodal intervention in functionally impaired older people with type 2 diabetes mellitus. J Cachexia Sarcopenia Muscle. 2019;10(4):721-733. https://pubmed.ncbi.nlm.nih.gov/31016897/*** | | | | |
| --- | --- | --- | --- | --- |
| **Study design** | **Study type** | **Country/ Region** | **Clinical setting** | **Duration** |
|  | Randomized controlled trial | 7 European countries | 74 trial sites | NR |
|  | **Intervention vs Control** | | **Sample size** | **Female (%)** |
|  | Multimodel intervention vs usual care | | 964 | 49.1 |
|  | **Age, years** | **Conditions** | **Follow-up** | **Loss to follow-up (%)** |
|  | Mean: 78 (range: 70+) | With type 2 diabetes mellitus. | 1 year | 18%* |
| **Frailty assessment** | **Measurement tool** | **Frailty assessment** | | **Frailty at baseline, Intervention vs Control** |
|  | Fried phenotype | Fried frailty criteria (range: 0-5 criteria) Frail [≥3 criteria] (N=364) Prefrail [1-2 criteria] (N=600) | | Frail: 33.1% 43.0% Pre-frail: 66.9% vs 57.0% |
| **Main findings** | **Outcome** | **Relative effect** | **Analytic approach** | **p-value for interaction** |
|  | 12-month SPPB | not available | interaction term | 0.49 |
| **Risk of bias** | **Random sequence generation** | **Allocation concealment** | **Blinding of participants and personnel** | **Blinding of outcome assessment** |
|  | Unclear | Low | High | High |
|  | **Incomplete outcome data** | **Selective reporting** | **Other bias** |  |
|  | High | Low | Low |  |

Abbreviations: NR, not reported; SPPB, Short Physical Performance Battery.

Reference:

*: Supplement data. Available at https://onlinelibrary.wiley.com/doi/10.1002/jcsm.12432.

| ***Rolland, et al. Strontium ranelate and risk of vertebral fractures in frail osteoporotic women. Bone. 2011;48(2):332-338. https://pubmed.ncbi.nlm.nih.gov/20817053/*** | | | | |
| --- | --- | --- | --- | --- |
| **Study design** | **Study type** | **Country/ Region** | **Clinical setting** | **Duration** |
|  | Secondary analysis of two RCTs | 12 countries | 75 centers | NR |
|  | **Intervention vs Control** | | **Sample size** | **Female (%)** |
|  | Strontium ranelate vs placebo | | 5082 | 100 |
|  | **Age, years** | **Conditions** | **Follow-up** | **Loss to follow-up (%)** |
|  | Mean Robust: 73.9 vs 73.8 Intermediate: 74.1 vs 74.0  Frail: 75.5 vs 75.6  (range: RCT-1 50+, RCT-2 70+) | Robust, intermediate and frail women. | 3 years | NR |
| **Frailty assessment** | **Measurement tool** | **Frailty assessment** | | **Frailty at baseline, Intervention vs Control** |
|  | Frailty phenotype | Fried frailty criteria (range: 0-5 criteria): Robust [0 criteria] (N=2346) Intermediate [1-2 of the 5 criteria] (N=2472) Frail [≥ 3 criteria] (N=264) | | Robust: 51.7% vs 48.3% Intermediate: 48.4% vs 51.6% Frail: 47.3% vs 52.7% |
| **Main findings** | **Outcome** | **Relative effect** | **Analytic approach** | **p-value for interaction** |
|  | 3-year Vertebral fracture | Robust: 0.70 [0.57, 0.86] Intermediate: 0.55 [0.46, 0.67] Frail: 0.42 [0.24, 0.74] | Stratified analysis | 0.11 |

Abbreviations: RCT, randomized controlled trial

References:

Original RCT-1: Meunier PJ, et al. The effects of strontium ranelate on the risk of vertebral fracture in women with postmenopausal osteoporosis. N Engl J Med. 2004;350(5):459-468.

Original RCT-2: Reginster JY, et al. Strontium ranelate reduces the risk of nonvertebral fractures in postmenopausal women with osteoporosis: Treatment of Peripheral Osteoporosis (TROPOS) study. J Clin Endocrinol Metab. 2005;90(5):2816-2822.

| ***Sanders et al. The frailty syndrome and outcomes in the TOPCAT trial. Eur J Heart Fail. 2018;20(11):1570-1577. https://pubmed.ncbi.nlm.nih.gov/30225878/*** | | | | |
| --- | --- | --- | --- | --- |
| **Study design** | **Study type** | **Country/ Region** | **Clinical setting** | **Duration** |
|  | Secondary analysis of the randomized controlled trial | United States, Canada, Brazil, Argentina | clinical sites* | August 2006-June 2013* |
|  | **Intervention vs Control** | | **Sample size** | **Female (%)** |
|  | Spironolactone 15–45 mg daily vs Placebo | | 1767 | 49.0 |
|  | **Age, years** | **Conditions** | **Follow-up** | **Loss to follow-up (%)** |
|  | Mean: 71.5 (range: 50+)† | Symptomatic heart failure with preserved ejection fraction | Mean: 3.3 years† | NA. Subset of the original RCT. |
| **Frailty assessment** | **Measurement tool** | **Frailty assessment** | | **Frailty at baseline, Intervention vs Control** |
|  | 39-item Frailty index | FI (range: 0-1 points): Class 1 [0, 0.3) (N=482) Class 2 [0.3, 0.4) (N=598) Class 3 [0.4, 0.5) (N=460) Class 4 [0.5, 1] (N=227) | | NR |
| **Main findings** | **Outcome** | **Relative effect ‡** | **Analytic approach** | ***P*-value of interaction** |
|  | 5-year composite measure of death from cardiovascular causes, aborted cardiac arrest or hospitalization for the management of heart failure | Class 1 HR 0.78 [0.52, 1.16] Class 2 HR 0.79 [0.58, 1.06] Class 3 HR 1.00 [0.73, 1.39] Class 4 HR 0.74 [0.51, 1.07] | Stratified analysis and interaction term | 0.55 |
|  | 5-year HF hospitalization | Class 1 HR 0.75 [0.46, 1.21] Class 2 HR 0.87 [0.62, 1.23] Class 3 HR 0.92 [0.63, 1.32] Class 4 HR 0.74 [0.49, 1.11] |  | 0.78 |
|  | 5-year CV mortality | Class 1 HR 0.77 [0.44, 1.34] Class 2 HR 0.59 [0.37, 0.93] Class 3 HR 1.11 [0.66, 1.87] Class 4 HR 0.59 [0.31, 1.13] |  | 0.24 |
|  | 5-year All-cause mortality | Class 1 HR 0.97 [0.63, 1.49] Class 2 HR 0.64 [0.45, 0.90] Class 3 HR 1.33 [0.91, 1.94] Class 4 HR 0.55 [0.33, 0.90] |  | 0.01 |

Abbreviations: NR, not reported; NA, not applicable; HF, heart failure; CV, cardiovascular.

Note: Primary outcome was a composite of death from cardiovascular causes, aborted cardiac arrest or hospitalization for the management of heart failure.

References:

*: Clinical Trial Registration: NCT00094302. Available at https://clinicaltrials.gov/ct2/show/NCT00094302?term=NCT00094302&draw=2&rank=1.

†:Original RCT. Pitt et al. Spironolactone for heart failure with preserved ejection fraction. N Engl J Med. 2014;370(15):1383-1392.

‡: Data were extracted by GetData Graph Digitizer software.

| ***Saxer, et al. Minimally invasive anterior muscle-sparing versus a transgluteal approach for hemiarthroplasty in femoral neck fractures-a prospective randomised controlled trial including 190 elderly patients. BMC Geriatr. 2018;18(1):222. https://pubmed.ncbi.nlm.nih.gov/30241509/*** | | | | |
| --- | --- | --- | --- | --- |
| **Study design** | **Study type** | **Country/ Region** | **Clinical setting** | **Duration** |
|  | Randomized controlled trial | Switzerland | One centre | August 2011-April 2016* |
|  | **Intervention vs Control** | | **Sample size** | **Female (%)** |
|  | Anterior minimally-invasive vs lateral Hardinge | | 181 | 71.3 |
|  | **Age, years** | **Conditions** | **Follow-up** | **Loss to follow-up (%)** |
|  | Mean: 84.2 (range: 60+) | Adults with femoral neck fractures | 1 year | 23.20% |
| **Frailty assessment** | **Measurement tool** | **Frailty assessment** | | **Frailty at baseline, Intervention vs Control** |
|  | Frailty index | Frailty index: (18-(Functional Independence Measure-18)/6 + Charlson Index + Medication score)/36 (range: 0-1 points) [0,0.25]  (0.25,1] (26% of total patients) | | Mean frailty index: 0.18 vs 0.17 |
| **Main findings** | **Outcome** | **Absolute effect** | **Analytic approach** | **p-value for interaction** |
|  | 12-month TUG performance | NA | Stratified analysis | >0.05 |
| **Risk of bias** | **Random sequence generation** | **Allocation concealment** | **Blinding of participants and personnel** | **Blinding of outcome assessment** |
|  | Unclear | Low | High | Low |
|  | **Incomplete outcome data** | **Selective reporting** | **Other bias** |  |
|  | High | Low | Low |  |

Abbreviations: TUG, timed up and go; NSD, no sufficient data; NR, not reported.

Note: The relative effects were nor reported.

References:

*: Clinical Trial Registration: NCT01408693. Available at https://www.clinicaltrials.gov/ct2/show/NCT01408693?term=NCT01408693&draw=2&rank=1.

†: Data were extracted by GetData Graph Digitizer software.

| ***Sheppard , et al. Effect of Antihypertensive Medication Reduction vs Usual Care on Short-term Blood Pressure Control in Patients With Hypertension Aged 80 Years and Older: The OPTIMISE Randomized Clinical Trial. JAMA. 2020;323(20):2039-2051. https://pubmed.ncbi.nlm.nih.gov/32453368/*** | | | | |
| --- | --- | --- | --- | --- |
| **Study design** | **Study type** | **Country/ Region** | **Clinical setting** | **Duration** |
|  | Randomized controlled trial | England | 69 primary care sites | July 1, 2016-December 31, 2024* |
|  | **Intervention vs Control** | | **Sample size** | **Female (%)** |
|  | Antihypertensive Medication Reduction vs usual care | | 569 | 48.5 |
|  | **Age, years** | **Conditions** | **Follow-up** | **Loss to follow-up (%)** |
|  | Mean: 84.8 (range: 80+) | Patients had a systolic blood pressure lower than 150 mm Hg, and were prescribed 2 or more antihypertensive treatments for at least 12 months | 12 weeks | 6.2% |
| **Frailty assessment** | **Measurement tool** | **Frailty assessment** | | **Frailty at baseline, Intervention vs Control** |
|  | Frailty index | Frailty index ≤ 0.12 (N=222) Frailty index ＞0.12 (N=312) | | NR |
| **Main findings** | **Outcome** | **Relative effect** | **Analytic approach** | **p-value for interaction** |
|  | 12-weeks Relative risk of systolic blood pressure control* | Frailty index ≤ 0.12: 0.94 [0.85, ∞] Frailty index ＞0.12: 1.01 [0.93, ∞] | Stratified analysis and interaction term | 0.35 |
|  | 12-weeks Mean difference in change in systolic blood pressure* | Frailty index ≤ 0.12: 5.14 [1.40, 8.87] Frailty index ＞0.12: 2.26 [-0.90, 5.42] |  | 0.25 |
| **Risk of bias** | **Random sequence generation** | **Allocation concealment** | **Blinding of participants and personnel** | **Blinding of outcome assessment** |
|  | Low | Low | High | Low |
|  | **Incomplete outcome data** | **Selective reporting** | **Other bias** |  |
|  | Low | Low | Low |  |

Abbreviations: NR, not reported.

Reference:

Clinical Trial Registration: ISRCTN97503221. Available at https://www.isrctn.com/ISRCTN97503221?q=97503221&filters=&sort=&offset=1&totalResults=1&page=1&pageSize=10&searchType=basic-search.

*: Supplement data. Available at https://jamanetwork.com/journals/jama/fullarticle/2766421?resultClick=1.

| ***Simpson et al. Does the Impact of Intensive Lifestyle Intervention on Cardiovascular Disease Risk Vary According to Frailty as Measured via Deficit Accumulation?. J Gerontol A Biol Sci Med Sci. 2021;76(2):339-345. https://pubmed.ncbi.nlm.nih.gov/32564066/*** | | | | |
| --- | --- | --- | --- | --- |
| **Study design** | **Study type** | **Country/ Region** | **Clinical setting** | **Duration** |
|  | Randomized controlled trial | United States | 16 US centers | June 2001-June 30, 2020* |
|  | **Intervention vs Control** | | **Sample size** | **Female (%)** |
|  | Intensive lifestyle intervention vs Diabetes support and education | | 5145 | 59.5† |
|  | **Age, years** | **Conditions** | **Follow-up** | **Loss to follow-up (%)** |
|  | Mean: 58.7 (Range: 45-75)† | Overweight or obese adults with Type 2 diabetes | Mean: 9.8 years (range: 8.4-11.1) | 3.7%‡ |
| **Frailty assessment** | **Measurement tool** | **Frailty assessment** | | **Frailty at baseline, Intervention vs Control** |
|  | Frailty index | 38-item FI (range: 0-1 points): 1st Tertile [0.066, 0.178) (N= 1606)  2nd Tertile [0.178, 0.230) (N=1634)  3rd Tertile [0.230, 0.588] (N=1619)§ | | 1st Tertile: 33.7% vs 32.4% 2nd Tertile: 32.8% vs 34.5% 3rd Tertile: 33.5% vs 33.1%§ |
| **Main findings** | **Outcome** | **Relative effect** | **Analytic approach** | ***P*-value of interaction** |
|  | 10-year composite CVD outcome | 1st Tertile: HR 0.73 [0.55, 0.98] 2nd Tertile: HR 0.97 [0.72, 1.17]  3rd Tertile: HR 1.15 [0.94, 1.42] | Stratified analysis and interaction term | 0.01 |
| **Risk of bias** | **Random sequence generation** | **Allocation concealment** | **Blinding of participants and personnel** | **Blinding of outcome assessment** |
|  | Low | Low | Low | Low |
|  | **Incomplete outcome data** | **Selective reporting** | **Other bias** |  |
|  | Low | Low | Unclear |  |

Abbreviations: CVD, cardiovascular disease; HR, hazard ratio.

Note: composite CVD outcome, included fatal and nonfatal myocardial infarction and stroke, hospitalized angina, or CVD death.

References:

*: Clinical Trial Registration: NCT00017953. Available at https://clinicaltrials.gov/ct2/show/NCT00017953?term=NCT00017953&draw=2&rank=1.

†: Look AHEAD Research Group et al. Cardiovascular effects of intensive lifestyle intervention in type 2 diabetes. N Engl J Med. 2013;369(2):145-154.

‡: Supplement data. Available at https://www.nejm.org/doi/full/10.1056/NEJMoa1212914#article_supplementary_material.

§: Supplement data. Available at https://academic.oup.com/biomedgerontology/article/76/2/339/5860463?searchresult=1#supplementary-data.

| ***Sink, et al. Syncope, Hypotension, and Falls in the Treatment of Hypertension: Results from the Randomized Clinical Systolic Blood Pressure Intervention Trial. J Am Geriatr Soc. 2018;66(4):679-686. https://pubmed.ncbi.nlm.nih.gov/29601076/*** | | | | |
| --- | --- | --- | --- | --- |
| **Study design** | **Study type** | **Country/ Region** | **Clinical setting** | **Duration** |
|  | Randomized controlled trial | United States | 102 Academic and private practices | October 2010-March 2019* |
|  | **Intervention vs Control** | | **Sample size** | **Female (%)** |
|  | Intensive BP control vs standard BP control | | 9361 | 35.6 |
|  | **Age, years** | **Conditions** | **Follow-up** | **Loss to follow-up (%)** |
|  | Range: 50+ | With a SBP of 130 to 180 mmHg. | Median: 3.26 years | NR |
| **Frailty assessment** | **Measurement tool** | **Frailty assessment** | | **Frailty at baseline, Intervention vs Control** |
|  | 36-item Frailty index | NR | | NR |
| **Main findings** | **Outcome** | **Relative effect** | **Analytic approach** | **p-value for interaction** |
|  | Syncope | NR | Interaction term | Baseline frailty did not modify the harmful relationship between intensive SBP control and the three outcomes (P >0.7 in all cases). |
|  | Hypotension |  |  |  |
|  | Falls |  |  |  |
| **Risk of bias** | **Random sequence generation** | **Allocation concealment** | **Blinding of participants and personnel** | **Blinding of outcome assessment** |
|  | Unclear | Unclear | High | Low |
|  | **Incomplete outcome data** | **Selective reporting** | **Other bias** |  |
|  | Unclear | Low | Low |  |

Abbreviations: BP, blood pressure; NR, not reported.

Reference:

*: Clinical Trial Registration: NCT01206062. Available at https://clinicaltrials.gov/ct2/show/NCT01206062?term=NCT01206062&draw=2&rank=1.

| ***Spoorenberg et al. Effects of a population-based, person-centred and integrated care service on health, wellbeing and self-management of community-living older adults: A randomised controlled trial on Embrace. PLoS One. 2018;13(1):e0190751. https://pubmed.ncbi.nlm.nih.gov/29351295/*** | | | | |
| --- | --- | --- | --- | --- |
| **Study design** | **Study type** | **Country/ Region** | **Clinical setting** | **Duration** |
|  | Randomized controlled trial | Netherlands | General practitioner practices* | January 1, 2012-April 1 2013* |
|  | **Intervention vs Control** | | **Sample size** | **Female (%)** |
|  | Embrace vs usual care | | 1456 | 54.2 |
|  | **Age, years** | **Conditions** | **Follow-up** | **Loss to follow-up (%)** |
|  | Mean: 80.6 vs 80.8 (range: 75+) | Community-living older adults | 12 months | 22%† |
| **Frailty assessment** | **Measurement tool** | **Frailty assessment** | | **Frailty at baseline, Intervention vs Control** |
|  | Other frailty assessment tool | INTERMED-E-SA* and GFI‡:  Complex care needs [INTERMED-E-SA≥16] (N=365) Frail (INTERMED-E-SA <16 and a GFI >=5] (N= 237) Robust (INTERMED-E-SA <16 and GFI <5) (N=854) | | Complex care needs: 25% vs 25% Frail: 16% vs 16% Robust: 59% vs 59% |
| **Main findings** | **Outcome** | **Relative effect** | **Analytic approach** | ***P*-value for interaction** |
|  | 12 months EQ-5D-3L | Complex care needs: Effect size d=0.07 Frail: Effect size d=0.16 Robust: Effect size d=0.03 | Stratified analysis | NR |
|  | 12 months EQ-VAS | Complex care needs: Effect size d=0.10 Frail: Effect size d=0.11 Robust: Effect size d=0.05 |  |  |
|  | 12 months INTERMED-E-SA | Complex care needs: Effect size d=0.15 Frail: Effect size d=0.06 Robust: Effect size d=0.04 |  |  |
|  | 12 months GFI | Complex care needs: Effect size d=0.06 Frail: Effect size d=0.07 Robust: Effect size d=0.06 |  |  |
|  | 12 months Katz-15 | Complex care needs: Effect size d=0.13 Frail: Effect size d=0.06 Robust: Effect size d=0.14 |  |  |
|  | 12 month GWI SF Score | Complex care needs: Effect size d=0.07 Frail: Effect size d=0.09 Robust: Effect size d=0.01 |  |  |
|  | 12 month QoL general | Complex care needs: Effect size d=0.06 Frail: Effect size d=0.03 Robust: Effect size d=0.07 |  |  |
|  | 12 month QoL vs 1 year ago | Complex care needs: Effect size d=0.08 Frail: Effect size d=0.10 Robust: Effect size d=0.16 |  |  |
|  | 12 month SMAS-30 | Complex care needs: Effect size d=0.26 Frail: Effect size d=0.05 Robust: Effect size d=0.03 |  |  |
|  | 12 month PIH-OA | Complex care needs: Effect size d=0.00 Frail: Effect size d=0.31 Robust: Effect size d=0.01 |  |  |
| **Risk of bias** | **Random sequence generation** | **Allocation concealment** | **Blinding of participants and personnel** | **Blinding of outcome assessment** |
|  | Low | Low | High | High |
|  | **Incomplete outcome data** | **Selective reporting** | **Other bias** |  |
|  | High | Low | Unclear |  |

Abbreviations: INTERMED-E-SA, INTERMED for the Elderly Self-Assessment; GFI, Groningen Frailty Indicator; NR, not reported.

Notes: The analysis based different frailty subgroups so that interaction p value not applicable.

*: Peters, et al. Development and measurement properties of the self assessment version of the INTERMED for the elderly to assess case complexity. J Psychosom Res. 2013 Jun;74(6):518-22. doi: 10.1016/j.jpsychores.2013.02.003. Epub 2013 Mar 7.

‡: Peters, et al. Measurement properties of the Groningen Frailty Indicator in home-dwelling and institutionalized elderly people. J Am Med Dir Assoc. 2012 Jul;13(6):546-51. doi: 10.1016/j.jamda.2012.04.007. Epub 2012 May 12.

| ***Srinivas-Shankar, et al. Effects of testosterone on muscle strength, physical function, body composition, and quality of life in intermediate-frail and frail elderly men: a randomized, double-blind, placebo-controlled study. J Clin Endocrinol Metab. 2010;95(2):639-650.  https://pubmed.ncbi.nlm.nih.gov/31788741/*** | | | | |
| --- | --- | --- | --- | --- |
| **Study design** | **Study type** | **Country/ Region** | **Clinical setting** | **Duration** |
|  | Randomized controlled trial | NR. Possibly UK. | NR | NR |
|  | **Intervention vs Control** | | **Sample size** | **Female (%)** |
|  | Testosterone vs Placebo | | 262 | 0.0 |
|  | **Age, years** | **Conditions** | **Follow-up** | **Loss to follow-up (%)** |
|  | Mean: 74 (range: 65+) | Community-dwelling intermediate-frail and frail elderly men | 6 months | 15.7% |
| **Frailty assessment** | **Measurement tool** | **Frailty assessment** | | **Frailty at baseline, Intervention vs Control** |
|  | Frailty phenotype | Fried frailty criteria (range: 0-5 criteria): 1 criteria (N=162) 2 criteria (N=62) 3 criteria (N=31) 4 criteria (N=7) | | 1 criteria: 63.8% vs 59.8% 2 criteria: 22.3% vs 25% 3 criteria: 12.3% vs 11.4% 4 criteria: 1.5% vs 3.8% |
| **Main findings** | **Outcome** | **Relative effect** | **Analytic approach** | ***P*-value for interaction** |
|  | 6-month LBM | Adjusted difference: 1.6 [0.8, 2.4] | Stratified analysis and Interaction term | 0.33 |
|  | 6-month IME-PT | Adjusted difference: 9.48 [-4.05, 23.02] |  | 0.68 |
|  | 6-month AMS Somatic subscale | Adjusted difference: -2.76 [-5.18, -0.34] |  | 0.03 |
|  | 6-month AMS psychological subscale | Adjusted difference: -1.61 [-3.47, 0.24] |  | 0.001 |
|  | 6-month AMS sexual subscale | Adjusted difference: -3.03 [-4.87, -1.20] |  | 0.02 |
|  | 6-month ALF score | Adjusted difference: -3.66 [-8.52, 1.20] |  | 0.004 |
|  | 6-month 6MWT (m) | Adjusted difference: 28.0 [1.5, 54.5] |  | 0.2 |
|  | 6-month Total PPT score | Adjusted difference: 1.65 [0.11, 3.20] |  | 0.01 |
|  | 6-month Tinetti balance | Adjusted difference: 0.06 [-0.89, 1.01] |  | 0.86 |
|  | 6-month Tinetti gait | Adjusted difference: 0.10 [-0.38, 0.57] |  | 0.67 |
| **Risk of bias** | **Random sequence generation** | **Allocation concealment** | **Blinding of participants and personnel** | **Blinding of outcome assessment** |
|  | Low | Low | Low | Low |
|  | **Incomplete outcome data** | **Selective reporting** | **Other bias** |  |
|  | High | Low | Low |  |

Abbreviations: NR, not reported

| ***Suikkanen, et al. Effects of Home-Based Physical Exercise on Days at Home and Cost-Effectiveness in Pre-Frail and Frail Persons: Randomized Controlled Trial. J Am Med Dir Assoc. 2021;22(4):773-779. https://pubmed.ncbi.nlm.nih.gov/32694001/*** | | | | |
| --- | --- | --- | --- | --- |
| **Study design** | **Study type** | **Country/ Region** | **Clinical setting** | **Duration** |
|  | Randomized controlled trial | Finland | Social and Health Care District* | December 2014-December 2021† |
|  | **Intervention vs Control** | | **Sample size** | **Female (%)** |
|  | Physical exercise vs Usual care | | 299 | 75.0 |
|  | **Age, years** | **Conditions** | **Follow-up** | **Loss to follow-up (%)** |
|  | Mean: 82.5 (range: 65+) | Home-dwelling persons meeting at least 1 of the frailty phenotype criteria, Mini-Mental State Examination score≥17 | 24 months | 20.1% |
| **Frailty assessment** | **Measurement tool** | **Frailty assessment** | | **Frailty at baseline, Intervention vs Control** |
|  | Frailty phenotype | Modified Fried frailty criteria Pre-frail [1-2 of the 5 Fried's criteria] (N=182) Frail [3-5 of the 5 Fried's criteria] (N=117) | | Pre-frail: 61% vs 61% Frail: 39% vs 39% |
| **Main findings** | **Outcome** | **Relative effect** | **Analytic approach** | ***P*-value for interaction** |
|  | Days at home in 24 months | Frail IRR 1.04 [0.96, 1.12] pre-frail IRR 1.03 [0.96, 1.11] | Stratified analysis | not available |
| **Risk of bias** | **Random sequence generation** | **Allocation concealment** | **Blinding of participants and personnel** | **Blinding of outcome assessment** |
|  | Low | Low | Low | Low |
|  | **Incomplete outcome data** | **Selective reporting** | **Other bias** |  |
|  | High | Low | Unclear |  |

Abbreviations: HRQoL, health-related quality-of-life; NR, not reported.

Notes: The analysis based different frailty subgroups so that interaction p value not applicable.

Reference:

*: Soukkio P, et al. Effects of 12-month home-based physiotherapy on duration of living at home and functional capacity among older persons with signs of frailty or with a recent hip fracture - protocol of a randomized controlled trial (HIPFRA study). BMC Geriatr. 2018;18(1):232.

†: Clinical Trial Registration: NCT02305433. Available at https://clinicaltrials.gov/ct2/show/NCT02305433?term=NCT02305433&draw=2&rank=1.

| ***Tabue-Teguo, et al. Effect of Multidomain Intervention, Omega-3 Polyunsaturated Fatty Acids Supplementation or their Combinaison on Cognitive Function in Non-Demented Older Adults According to Frail Status: Results from the MAPT Study. J Nutr Health Aging. 2018;22(8):923-927. https://pubmed.ncbi.nlm.nih.gov/30272094/*** | | | | |
| --- | --- | --- | --- | --- |
| **Study design** | **Study type** | **Country/ Region** | **Clinical setting** | **Duration** |
|  | Secondary analysis of the randomized controlled trial | France | 13 memory clinics | December 2011-July 20, 2020* |
|  | **Intervention vs Control** | | **Sample size** | **Female (%)** |
|  | ①MI + n3 PUFA vs Placebo; ②n3 PUFA vs Placebo; ③MI + Placebo vs Placebo | | 1464 | 64.2 |
|  | **Age, years** | **Conditions** | **Follow-up** | **Loss to follow-up (%)** |
|  | Mean: 75.25 (range: 70+) | At risk of cognitive decline† | 3 years | NA. Subset of the original RCT. |
| **Frailty assessment** | **Measurement tool** | **Frailty assessment** | | **Frailty at baseline** |
|  | Frailty phenotype | Fried frailty phenotype: Non-frail [Frailty=0] (N=799) Pre-frail [Frailty＞0] (N=665) | | Involuntary weight loss: 4.6% Exhaustion: 15.8% Weakness (handgrip strength): 23.3% Slow gait speed: 3.9% Low physical acitivity: 14.3% |
| **Main findings** | **Outcome** | **Abosulte effect** | **Analytic approach** | **p-value for interaction** |
|  | 3-year TMTA | Difference of score change ①Pre-frail: -4.029 Non-frail: 1.134 ②Pre-frail: 0.246 Non-frail: 3.478 ③Pre-frail: 2.268 Non-frail: 2.951 | Stratified analysis | ①0.031 ②0.174 ③0.774 |
|  | 3-year TMTB | Difference of score change ①Pre-frail: -4.093 Non-frail: -1.466 ②Pre-frail: -6.313 Non-frail: 1.798 ③Pre-frail: 12.891 Non-frail: -4.297 |  | ①0.728 ②0.284 ③0.024 |
|  | 3-year Free and total recall FCSRT | Difference of score change ①Pre-frail: -0.935 Non-frail: 0.295 ②Pre-frail: -1.847 Non-frail: -0.390 ③Pre-frail: -0.836 Non-frail: -0.027 |  | ①0.344 ②0.259 ③0.532 |
|  | 3-year MMSE | Difference of score change ①Pre-frail: 0.001 Non-frail: 0.187 ②Pre-frail: -0.131 Non-frail: 0.076 ③Pre-frail: 0.093 Non-frail: 0.136 |  | ①0.502 ②0.454 ③0.876 |
|  | 3-year DSST | Difference of score change ①Pre-frail: -0.632 Non-frail: 0.159 ②Pre-frail: 0.196 Non-frail: 0.202 ③Pre-frail: 0.841 Non-frail: 0.153 |  | ①0.407 ②0.994 ③0.469 |
|  | 3-year COWAT | Difference of score change ①Pre-frail: 0.182 Non-frail: 0.243 ②Pre-frail: 0.363 Non-frail: 0.639 ③Pre-frail: 0.542 Non-frail: 0.722 |  | ①0.939 ②0.734 ③0.825 |
|  | 3-year CNT | Difference of score change ①Pre-frail: -0.474 Non-frail: 0.306 ②Pre-frail: -0.163 Non-frail: -0.154 ③Pre-frail: -0.124 Non-frail: 0.217 |  | ①0.424 ②0.992 ③0.725 |

Abbreviations: MI, multidomain intervention; n3 PUFA, Omega-3 Polyunsaturated Fatty Acids; NA, not applicable; FCSRT, Free and Cued Selective Reminding test , DSST, Digit Symbol Substitution Test, COWAT, Controlled Oral Word Association Test; CNT, Category Naming Test , MMSE, Mini Mental State Examination, TMT, Trail Making Test.

Note: Relative effects were not applicable.

Reference:

*: Clinical Trial Registration: NCT01513252. Available at https://clinicaltrials.gov/ct2/show/NCT01513252?term=NCT01513252&draw=2&rank=1.

†: Original RCT. Vellas et al. MAPT STUDY: A MULTIDOMAIN APPROACH FOR PREVENTING ALZHEIMER'S DISEASE: DESIGN AND BASELINE DATA. J Prev Alzheimers Dis. 2014;1(1):13-22.

| ***Tieland, et al. Handgrip strength does not represent an appropriate measure to evaluate changes in muscle strength during an exercise intervention program in frail older people. Int J Sport Nutr Exerc Metab. 2015;25(1):27-36. https://pubmed.ncbi.nlm.nih.gov/24903908/*** | | | | |
| --- | --- | --- | --- | --- |
| **Study design** | **Study type** | **Country/ Region** | **Clinical setting** | **Duration** |
|  | Randomized controlled trial | Netherlands | NR | NR |
|  | **Intervention vs Control** | | **Sample size** | **Female (%)** |
|  | Resistance-type exercise training vs no exercise training | | 127 | 61.0 |
|  | **Age, years** | **Conditions** | **Follow-up** | **Loss to follow-up (%)** |
|  | Mean: 79 (range: 65+) | Prefrail and frail older people. | 24 weeks | 15.0% |
| **Frailty assessment** | **Measurement tool** | **Frailty assessment** | | **Frailty at baseline, Intervention vs Control** |
|  | Frailty phenotype | Fried frailty criteria (range: 0-5 criteria): Pre-frail [1-2 of the 5 criteria] Frail [3-5 of the 5 criteria] | | Pre-frail: 72% vs 84% Frail: 28% vs 16% |
| **Main findings** | **Outcome** | **Relative effect** | **Analytic approach** | **p-value for interaction** |
|  | 24-weeks hominant handgrip strength | NR | Interaction term | ＞0.05 |
| **Risk of bias** | **Random sequence generation** | **Allocation concealment** | **Blinding of participants and personnel** | **Blinding of outcome assessment** |
|  | Low | Low | Low | Low |
|  | **Incomplete outcome data** | **Selective reporting** | **Other bias** |  |
|  | Low | Low | Low |  |

Abbreviations: NR, not reported.

| ***Trombetti et al. Effect of Physical Activity on Frailty: Secondary Analysis of a Randomized Controlled Trial. Ann Intern Med. 2018;168(5):309-316. https://pubmed.ncbi.nlm.nih.gov/29310138/*** | | | | |
| --- | --- | --- | --- | --- |
| **Study design** | **Study type** | **Country/ Region** | **Clinical setting** | **Duration** |
|  | Secondary analysis of the randomized controlled trial | United States | 8 centers | February 2010-December 2013 |
|  | **Intervention vs Control** | | **Sample size** | **Female (%)** |
|  | Physical activity vs Health education | | 1623 | 67.2 |
|  | **Age, years** | **Conditions** | **Follow-up** | **Loss to follow-up (%)** |
|  | Mean: 78.9 (range: 70-89) | With functional limitations | 2 years | NA. Subset of the original RCT. |
| **Frailty assessment** | **Measurement tool** | **Frailty assessment** | | **Frailty at baseline, Intervention vs Control** |
|  | SOF Frailty index | Study of Osteoporotic Fractures index (range: 0-3 criteria): Frail [>=2 criteria] (N=319) Not frail [＜2 criteria] (N=1304) | | Frail: 19.6% vs 19.7% Not frail: 80.4% vs 80.3% |
| **Main findings** | **Outcome** | **Relative effect, HR*** | **Analytic approach** | ***P*-value of interaction** |
|  | 2- and 3-year major mobility disability | 2 year non-Frail: 0.92 Frail: 0.96 3-year non frail: 0.97 frail: 1.02 | Stratified analysis and interaction term | 0.91 |
|  | 2- and 3-year persistent mobility disability | 2 year non-Frail: 0.92 Frail: 0.94 3-year non frail: 0.97 frail: 1.03 |  | 0.64 |

Abbreviations: NR, not reported; NA, not applicable.

References:

Clinical Trial Registration: NCT01072500. Available at https://clinicaltrials.gov/ct2/show/NCT01072500?term=NCT01072500&draw=2&rank=1.

Original RCT: Pahor et al. Effect of structured physical activity on prevention of major mobility disability in older adults: the LIFE study randomized clinical trial. JAMA. 2014;311(23):2387-2396.

*: Calculations are based on published data in the literature.

| ***Uittenbroek et al. Integrated Care for Older Adults Improves Perceived Quality of Care: Results of a Randomized Controlled Trial of Embrace. J Gen Intern Med. 2017;32(5):516-523. https://pubmed.ncbi.nlm.nih.gov/27271728/*** | | | | |
| --- | --- | --- | --- | --- |
| **Study design** | **Study type** | **Country/ Region** | **Clinical setting** | **Duration** |
|  | Randomized controlled trial | Netherlands* | General practitioner practices* | January 1, 2012-April 1 2013† |
|  | **Intervention vs Control** | | **Sample size** | **Female (%)** |
|  | Embrace vs usual care* | | 1456* | 54.2* |
|  | **Age, years** | **Conditions** | **Follow-up** | **Loss to follow-up (%)** |
|  | Mean: 80.7 vs 80.8 (range: 75+) | Community-living older adults* | 12 months* | 22.0% |
| **Frailty assessment** | **Measurement tool** | **Frailty assessment** | | **Frailty at baseline, Intervention vs Control** |
|  | Other frailty assessment tool | INTERMED-E-SA (range: 0-20)‡ and GFI (range: 0-15)*§:  Complex care needs [INTERMED-E-SA≥16] (N=365) Frail [INTERMED-E-SA <16 and a GFI >=5] (N= 237) Robust [INTERMED-E-SA <16 and GFI <5] (N=854) | | Complex care needs: 25% vs 25% Frail: 16% vs 16% Robust: 59% vs 59%* |
| **Main findings** | **Outcome** | **Relative effect** | **Analytic approach** | ***P*-value for interaction** |
|  | 12 month PAIEC total score | Complex care needs: 0.44 [0.01, 0.87] Frail: B 0.89 [0.42, 1.37] Robust: B 0.13 [-0.07, 0.33] | Stratified analysis | NR |
|  | 12 month PAIEC activation | Complex care needs: 0.54 [0.04, 1.05] Frail: B 0.77 [0.21, 1.32] Robust: B 0.11 [-0.13, 0.35] |  |  |
|  | 12 month PAIEC goal | Complex care needs: 0.48 [-0.00, 0. 96] Frail: B 0.56 [0.01, 1.11] Robust: B 0.12 [-0.10, 0.33] |  |  |
|  | 12 month PAIEC coordination | Complex care needs: 0.23 [-0.22, 0.69] Frail: B 1.32 [0.75, 1.90] Robust: B 0.16 [-0.04, 0.36] |  |  |
| **Risk of bias** | **Random sequence generation** | **Allocation concealment** | **Blinding of participants and personnel** | **Blinding of outcome assessment** |
|  | Low | Low | High | High |
|  | **Incomplete outcome data** | **Selective reporting** | **Other bias** |  |
|  | High | Low | Unclear |  |

Abbreviations: PAIEC, Patient Assessment of Integrated Elderly Care; B, unstandardized regression coefficient.

Notes: The analysis based different frailty subgroups so that interaction p value not applicable.

Reference:

*: Spoorenberg, et al. Effects of a population-based, person-centred and integrated care service on health, wellbeing and self-management of community-living older adults: A randomised controlled trial on Embrace. PLoS One. 2018;13(1):e0190751.

†: Clinical Trial Registration: NTR 3039. Available at <https://www.trialregister.nl/trial/2893.>

‡: Peters, et al. Development and measurement properties of the self assessment version of the INTERMED for the elderly to assess case complexity. J Psychosom Res. 2013 Jun;74(6):518-22. doi: 10.1016/j.jpsychores.2013.02.003. Epub 2013 Mar 7.

§: Peters, et al. Measurement properties of the Groningen Frailty Indicator in home-dwelling and institutionalized elderly people. J Am Med Dir Assoc. 2012 Jul;13(6):546-51. doi: 10.1016/j.jamda.2012.04.007. Epub 2012 May 12.

| ***Vart, et al. Efficacy and Safety of Dapagliflozin in Patients with Chronic Kidney Disease across the Spectrum of Frailty. J Gerontol A Biol Sci Med Sci. 2023 Aug 1:glad181. doi: 10.1093/gerona/glad181. Epub ahead of print.***  ***https://pubmed.ncbi.nlm.nih.gov/37527836/*** | | | | |
| --- | --- | --- | --- | --- |
| **Study design** | **Study type** | **Country/ Region** | **Clinical setting** | **Duration** |
|  | Secondary analysis of the randomized controlled trial | 21 countries | 386 sites | February 2017 - June  2020 |
|  | **Intervention vs Control** | | **Sample size** | **Female (%)** |
|  | Dapagliflozin (10 mg/day) vs Placebo | | 4303 | 33.1 |
|  | **Age, years** | **Conditions** | **Follow-up** | **Loss to follow-up (%)** |
|  | Mean:  Not-to-mildly Frail: 53.9  Moderately Frail: 63.3  Severely Frail: 66.4 | Adults with CKD, with/without type 2 diabetes, with an estimated glomerular filtration rate of 25–75 mL/min/1.73 m2, and urinary albumin-to-creatinine ratio 200–5 000 mg/g | Median: 2 years | NA |
| **Frailty assessment** | **Measurement tool** | **Frailty assessment** | | **Frailty at baseline, Intervention vs Control** |
|  | Frailty index | 32-item frailty index (Rockwood cumulative deficit approach, range: 0-1)  Not-to-mildly frail [FI ≤ 0.210] (N=1 162)  Moderately frail [FI 0.211< FI ≤ 0.310] (N=1642)  Severely frail [FI >0.311] (N=1499) | | Mean: 0.273 |
| **Main findings** | **Outcome** | **Relative effect** | **Analytic approach** | ***P*-value** |
|  | 2-year eGFR decline ≥ 50%, end-stage kidney disease, or kidney or cardiovascular death | Not-to-mildly frail: HR 0.50 (0.33, 0.76)  Moderately frail: HR 0.62 (0.45, 0.85)  Severely frail: HR 0.64 (0.49, 0.83) | Stratified analysis and interaction term | 0.667 |
|  | 2-year Kidney composite outcome: eGFR decline ≥ 50%, end-stage kidney disease or kidney death | Not-to-mildly frail: HR 0.42 (0.27, 0.67)  Moderately frail: HR 0.62 (0.44, 0.87)  Severely frail: HR 0.57 (0.41, 0.9) |  | 0.437 |
|  | 2-year Cardiovascular outcome: Hospitalization for heart failure or cardiovascular death | Not-to-mildly frail: HR 1.02 (0.43, 2.41)  Moderately frail: HR 0.70 (0.40, 1.24)  Severely frail: HR 0.67 (0.49, 0.92) |  | 0.627 |
|  | 2-year All-cause mortality | Not-to-mildly frail: HR 1.03 (0.45, 2.34)  Moderately frail: HR 0.56 (0.34, 0.90)  Severely frail: HR 0.69 (0.50, 0.96) |  | 0.417 |

Abbreviations: CKD, chronic kidney disease; eGFR, estimated glomerular filtration rate; NR, not reported.

| ***Warwick et al. No evidence that frailty modifies the positive impact of antihypertensive treatment in very elderly people: an investigation of the impact of frailty upon treatment effect in the HYpertension in the Very Elderly Trial (HYVET) study, a double-blind, placebo-controlled study of antihypertensives in people with hypertension aged 80 and over. BMC Med. 2015;13:78. https://pubmed.ncbi.nlm.nih.gov/25880068/*** | | | | |
| --- | --- | --- | --- | --- |
| **Study design** | **Study type** | **Country/ Region** | **Clinical setting** | **Duration** |
|  | Secondary analysis of the randomized controlled trial | 13 countries in Europe, China, Australasia, and Tunisia* | 195 centers* | November 2000-October 2008† |
|  | **Intervention vs Control** | | **Sample size** | **Female (%)** |
|  | Indaoamide ± perindopril vs Placebo | | 2656 | 60.6 |
|  | **Age, years** | **Conditions** | **Follow-up** | **Loss to follow-up (%)** |
|  | Mean 83.6 (range: 80+)* | People with hypertension | Median: 1.8 years* | NA. Subset of the original RCT. |
| **Frailty assessment** | **Measurement tool** | **Frailty assessment** | | **Frailty at baseline, Intervention vs Control** |
|  | Frailty index | FI based on 60 deficits (range: 0-1 points):  FI 0.1  FI 0.2  FI 0.3  FI 0.4  FI 0.5  FI 0.6 NSD | | FI median: 0.16 vs 0.17 |
| **Main findings** | **Outcome** | **Relative effect** | **Analytic approach** | ***P*-value for interaction** |
|  | 4-year Stroke | FI 0.1: HR 0.75 [0.40, 1.38] FI 0.2: HR 0.66 [0.43, 1.01] FI 0.3: HR 0.59 [0.36, 0.96] FI 0.4: HR 0.52 [0.25, 1.09] FI 0.5: HR 0.47 [0.16, 1.33] FI 0.6: HR 0.41 [0.10, 0.65] | Stratified analysis and interaction term | 0.52 |
|  | 4-year Cardiovascular events | FI 0.1: HR 0.62 [0.42, 0.92] FI 0.2: HR 0.60 [0.45, 0.78] FI 0.3: HR 0.57 [0.42, 0.79] FI 0.4: HR 0.55 [0.34, 0.89] FI 0.5: HR 0.53 [0.26, 1.06] FI 0.6: HR 0.50 [0.20, 1.27] |  | 0.73 |
|  | 4-year Total mortality | FI 0.1: HR 0.89 [0.63, 1.25] FI 0.2: HR 0.84 [0.66, 1.07] FI 0.3: HR 0.80 [0.61, 1.04] FI 0.4: HR 0.76 [0.50, 1.14] FI 0.5: HR 0.72 [0.40, 1.29] FI 0.6: HR 0.68 [0.32, 1.48] |  | 0.61 |

Abbreviations: NA, not applicable; NSD, no sufficient data; HR, hazard ratio; NR, not reported.

References:

*: Original RCT. Beckett et al. Treatment of hypertension in patients 80 years of age or older. N Engl J Med. 2008;358(18):1887-1898.

†: Clinical Trial Registration: NCT00122811. Available at https://clinicaltrials.gov/ct2/show/NCT00122811?term=NCT00122811&draw=2&rank=1.

| ***White, et al. Frailty is associated with worse outcomes in non-ST-segment elevation acute coronary syndromes: Insights from the TaRgeted platelet Inhibition to cLarify the Optimal strateGy to medicallY manage Acute Coronary Syndromes (TRILOGY ACS) trial. Eur Heart J Acute Cardiovasc Care. 2016;5(3):231-242. https://pubmed.ncbi.nlm.nih.gov/25897147/*** | | | | |
| --- | --- | --- | --- | --- |
| **Study design** | **Study type** | **Country/ Region** | **Clinical setting** | **Duration** |
|  | Secondary analysis of the randomized controlled trial | 52 countries* | 966 sites* | June 2008-April 2012† |
|  | **Intervention vs Control** | | **Sample size** | **Female (%)** |
|  | Prasugrel vs Clopidogrel | | 4996 | 46.1 |
|  | **Age, years** | **Conditions** | **Follow-up** | **Loss to follow-up (%)** |
|  | Not frail: median 73 Pre-frail: median 74 Frail: median 75 (range: 65+) | High-risk patients with unstable angina/NSTEMI. | Median 17.1 months (10.4–24.4) | 6.1%* |
| **Frailty assessment** | **Measurement tool** | **Frailty assessment** | | **Frailty at baseline** |
|  | Frailty phenotype | Fried frailty phenotype (range: 5 criteria):  Not frail [none of the 5 criteria] (N=3612) Pre-frail [1-2 of the 5 criteria] (N=1147) Frail [3-5 of the 5 criteria](N=237) | | Not frail: 72.3% Pre-frail: 23.0% Frail: 4.7% |
| **Main findings** | **Outcome** | **Relative effect** | **Analytic approach** | ***P*-value for interactions** |
|  | 3-month Cardiovascular death, MI, or Stroke | Not frail: HR 0.90 [0.77, 1.06] Pre-frail: HR 1.34 [1.04, 1.73] Frail: HR 0.89 [0.54, 1.46] | Stratified analysis and interaction term | 0.032 |
|  | 3-month Cardiovascular death | Not frail: HR 0.87 [0.69, 1.08] Pre-frail: HR 1.22 [0.87, 1.72] Frail: HR 0.98 [0.51, 1.87] |  | 0.252 |
|  | 3-month Myocardial infarction | Not frail: HR 0.95 [0.76, 1.18] Pre-frail: HR 1.22 [0.87, 1.70] Frail: HR 1.19 [0.57, 2.51] |  | 0.434 |
|  | 3-month Stroke | Not frail: HR 0.95 [0.57, 1.58] Pre-frail: HR 1.65 [0.79, 3.44] Frail: HR 0.73 [0.12, 4.51] |  | 0.427 |
|  | 3-month All-cause death | Not frail: HR 0.96 [0.78, 1.17] Pre-frail: HR 0.97 [0.72, 1.30] Frail: HR 0.79 [0.44, 1.41] |  | 0.81 |
|  | 3-month GUSTO severe or life-threatening bleeding | Not frail: HR 0.56 [0.26, 1.21] Pre-frail: HR 1.12 [0.30, 4.19] Frail: NE |  | 0.671 |
|  | 3-month GUSTO severe or life-threatening or moderate bleeding | Not frail: HR 0.93 [0.60, 1.45] Pre-frail: HR 1.57 [0.69, 3.58] Frail: NE |  | 0.541 |
|  | 3-month TIMI major bleeding | Not frail: HR 0.87 [0.51, 1.51] Pre-frail: HR 0.90 [0.29, 2.79] Frail: NE |  | 0.999 |
|  | 3-month TIMI major or minor bleeding | Not frail: HR 0.94 [0.60, 1.47] Pre-frail: HR 0.93 [0.42, 2.08] Frail: HR 2.85 [0.31, 25.83] |  | 0.625 |

Abbreviations: NR, not reported; HR, hazard ratio; GUSTO, Global Use of Strategies to Open Occluded Arteries; MI, myocardial infarction; NE, hazard ratio not computed due to insufficient data; TIMI, Thrombolysis In Myocardial Infarction.

Note: 52 countries in Europe, Scandinavia, Latin America, East Asia, India, North America, Mediterranean, Australia, New Zealand, and South Africa area.

Reference:

*: Roe et al. Prasugrel versus clopidogrel for acute coronary syndromes without revascularization. N Engl J Med. 2012;367(14):1297-1309.

†: Clinical Trial Registration: NCT00699998. Available at https://clinicaltrials.gov/ct2/show/NCT00699998?term=NCT00699998&draw=2&rank=1.

| ***Wilkinson et al. Clinical outcomes in patients with atrial fibrillation and frailty: insights from the ENGAGE AF-TIMI 48 trial. BMC Med. 2020;18(1):401. https://pubmed.ncbi.nlm.nih.gov/33357217/*** | | | | |
| --- | --- | --- | --- | --- |
| **Study design** | **Study type** | **Country/ Region** | **Clinical setting** | **Duration** |
|  | Secondary of the randomized controlled trial | 46 countries* | 1393 centres | November 2008-May 2013† |
|  | **Intervention vs Control** | | **Sample size** | **Female (%)** |
|  | Edoxaban 60 mg vs Edoxaban 30 mg vs Warfarin | | 20867 | 38.1 |
|  | **Age, years** | **Conditions** | **Follow-up** | **Loss to follow-up (%)** |
|  | Median: 72 (range: 60+)‡ | Patients with AF and a moderate or high risk of stroke | Median: 2.8 years | NA. Subset of the original RCT |
| **Frailty assessment** | **Measurement tool** | **Frailty assessment** | | **Frailty at baseline, Intervention vs Control** |
|  | 40-item Frailty index | FI (range: 0-1 points):  Fit [0, 0.12) (N=4459) Pre-frailty [0.12, 0.24) (N=12326) Mild-moderate [0.24, 0.36) (N=3722) Severe frailty [0.36,1.0) (N=360) | | Fit: 33.8% vs 33.0% vs 33.2% Pre-frail: 33.1% vs 33.4% vs 33.5% Mild-moderate: 33.4% vs 33.5% vs 33.0% Severe: 35.6% vs 31.7% vs 32.8% |
| **Main findings** | **Outcome** | **Relative effect** | **Analytic approach** | **p-value for interaction** |
|  | Time to first adjudicated stroke or systemic embolism | Edoxaban 60 mg vs Edoxaban 30 mg vs Warfarin (ref) Fit: HR 1.03 [0.71, 1.49] vs HR 1.04 [0.71, 1.50] Pre-frail: HR 0.82 [0.66, 1.01] vs HR 1.18 [0.97, 1.43] Mild-moderate: HR 0.84 [0.61, 1.15] vs HR 1.17 [0.87, 1.56] Severe: HR 0.54 [0.20, 1.50] vs HR 0.30 [0.08, 1.11] | Stratified analysis and interaction term | NR. There was no difference when stratified by frailty category. |
|  | Time to adjudicated major bleeding during treatment | Fit: HR 0.96 [0.71, 1.30] vs HR 0.42 [0.28, 0.62] Pre-frail: HR 0.76 [0.64, 0.90] vs HR 0.46 [0.38, 0.56] Mild-moderate: HR 0.75 [0.57, 0.98] vs HR 0.47 [0.35, 0.64] Severe: HR 0.60 [0.29, 1.26] vs HR 0.74 [0.36, 1.52] |  | NR. Edoxaban 30 mg was associated with a reduction in major bleeding compared with warfarin in all but those with severe frailty, and edoxaban 60 mg with a reduction in major bleeding in the pre-frail and mild-moderate frailty groups only. |
|  | Stroke, systemic embolic event, major bleeding, or death | Fit: HR 1.11 [0.92, 1.34] vs HR 0.90 [0.74, 1.10] Pre-frail: HR 0.84 [0.76, 0.93] vs HR 0.83 [0.75, 0.92] Mild-moderate: HR 0.90 [0.78, 1.03] vs HR 0.81 [0.70, 0.93] Severe: HR 0.75 [0.50, 1.12] vs HR 0.82 [0.54, 1.23] |  | NR.  Fit & Severe: no difference. Pre-frailty: reduced risk. Mild-moderate: reduced risk within edoxaban 30 mg arm; no difference within edoxaban 60 mg arm. |
|  | Disabling stroke, life-threatening bleeding, or death | Fit: HR 1.11 [0.87, 1.41] vs HR 1.01 [0.79, 1.30] Pre-frail: HR 0.86 [0.76, 0.97] vs HR 0.83 [0.74, 0.95] Mild-moderate: HR 0.88 [0.74, 1.04] vs HR 0.79 [0.66, 0.94] Severe: HR 0.62 [0.39, 0.99] vs HR 0.66 [0.41, 1.07] |  | NR.  Fit: no difference. Pre-frailty: reduced risk. Mild-moderate: reduced risk within edoxaban 30 mg arm; no difference within edoxaban 60 mg arm. Severe: reduced risk within edoxaban 60 mg arm. |
|  | Stroke, systemic embolic event, life-threatening bleeding, or death | Fit: HR 1.14 [0.91, 1.42] vs HR 1.03 [0.82, 1.30] Pre-frail: HR 0.83 [0.74, 0.93] vs HR 0.89 [0.79, 1.00] Mild-moderate: HR 0.88 [0.75, 1.04] vs HR 0.85 [0.72, 1.00] Severe: HR 0.66 [0.42, 1.03] vs HR 0.64 [0.40, 1.02] |  | NR.  Fit & Mild-moderate & Severe: no difference. Pre-frailty: reduction effect. |
|  | Death | Fit: HR 1.20 [0.92, 1.56] vs HR 1.08 [0.83, 1.42] Pre-frail: HR 0.88 [0.77, 1.01] vs HR 0.89 [0.78, 1.02] Mild-moderate: HR 0.89 [0.75, 1.07] vs HR 0.83 [0.69, 1.00] Severe: HR 0.69 [0.43, 1.12] vs HR 0.67 [0.41, 1.12] |  | NR. There was a stepwise association between frailty category and mortality. |

Abbreviations: AF, Atrial fibrillation; NA, not applicable; NR, not reported; HR, hazard ratio.

Note: 46 countries, inlucded Argentina, Australia, Belgium, Brazil, Bulgaria, Canada, Chile, China, Colombia, Croatia, Czech Republic, Denmark, Estonia, Finland, France, Germany, Greece, Guatemala, Hungary, India, Israel, Italy, Japan, Mexico, Netherlands, New Zealand, Norway, Peru, Philippines, Poland, Portugal, Romania, Russian Federation, Serbia, Slovakia, South Africa, South Korea, Spain, Sweden, Switzerland, Taiwan, Thailand, Turkey, Ukraine, United Kingdom, United States.

References:

*: Supplement dada. Available at https://www.nejm.org/doi/full/10.1056/NEJMoa1310907.

†: Clinical Trial Registration: NCT00781391. Available at https://clinicaltrials.gov/ct2/show/NCT00781391?term=NCT00781391&draw=2&rank=1.

‡: Original RCT. Ruff et al. Evaluation of the novel factor Xa inhibitor edoxaban compared with warfarin in patients with atrial fibrillation: design and rationale for the Effective aNticoaGulation with factor xA next GEneration in Atrial Fibrillation-Thrombolysis In Myocardial Infarction study 48 (ENGAGE AF-TIMI 48). Am Heart J. 2010;160(4):635-641.

§: Giugliano et al. Edoxaban versus warfarin in patients with atrial fibrillation. N Engl J Med. 2013;369(22):2093-2104.

| ***Williamson et al. Intensive vs Standard Blood Pressure Control and Cardiovascular Disease Outcomes in Adults Aged ≥75 Years: A Randomized Clinical Trial. JAMA. 2016;315(24):2673-2682. https://pubmed.ncbi.nlm.nih.gov/27195814/*** | | | | |
| --- | --- | --- | --- | --- |
| **Study design** | **Study type** | **Country/ Region** | **Clinical setting** | **Duration** |
|  | Randomized controlled trial | United States | clinics* | October 2010-March 2019† |
|  | **Intervention vs Control** | | **Sample size** | **Female (%)** |
|  | Intensive (<120 mm Hg) systolic blood pressure (SBP) targets vs Standard (<140 mm Hg) SBP targets | | 2636 | 37.9 |
|  | **Age, years** | **Conditions** | **Follow-up** | **Loss to follow-up (%)** |
|  | Mean: 79.9 (range: 75+) | With an average baseline systolic blood pressure ≥130 mm Hg and evidence of cardiovascular disease, chronic kidney disease, 10-year Framingham cardiovascular disease risk score ≥15%, or age ≥75 years.* | Median: 3.14 years | 10.9% |
| **Frailty assessment** | **Measurement tool** | **Frailty assessment** | | **Frailty at baseline, Intervention vs Control** |
|  | Frailty index | 37-item frailty index (range: 0-1 points):  Fit [0, 0.10] (N=349)  Less fit (0.10, 0.21] (N=1456)  Frail (0.21,1] (N=815) | | Fit: 12.1% vs 14.4% Less fit: 54.0% vs 56.5% Frail: 33.4% vs 28.4% |
| **Main findings** | **Outcome** | **Relative effect** | **Analytic approach** | ***P*-value for interaction** |
|  | 6-year composite cardiovascular disease (primary outcome) | Fit: HR 0.47 [0.13, 1.39] Less fit: HR 0.63 [0.43, 0.91] Frail: HR 0.68 [0.45, 1.01] | Stratified analysis and interaction term | 0.84 |
|  | 6-year All-cause mortality | Fit: HR 0.95 [0.27, 3.15] Less fit: HR 0.48 [0.29, 0.78] Frail: HR 0.64 [0.41, 1.01] |  | 0.52 |
|  | 6-year Primary outcome + All-cause mortality | Fit: HR 0.71 [0.28, 1.69] Less fit: HR 0.60 [0.44, 0.83] Frail: HR 0.67 [0.48, 0.95] |  | 0.88 |
| **Risk of bias** | **Random sequence generation** | **Allocation concealment** | **Blinding of participants and personnel** | **Blinding of outcome assessment** |
|  | Unclear | Unclear | High | Low |
|  | **Incomplete outcome data** | **Selective reporting** | **Other bias** |  |
|  | Low | Low | Unclear |  |

Abbreviation: HR, hazard ratio.

Note: Primary outcome includes nonfatal myocardial infarction, acute coronary syndrome not resulting in a myocardial infarction, nonfatal stroke, nonfatal acute decompensated heart failure, and death from cardiovascular causes.

References:

*: Original RCT. Ambrosius et al. The design and rationale of a multicenter clinical trial comparing two strategies for control of systolic blood pressure: the Systolic Blood Pressure Intervention Trial (SPRINT) [published correction appears in Clin Trials. 2017 Apr;14(2):222]. Clin Trials. 2014;11(5):532-546.

†: Clinical Trial Registration: NCT01206062. Available at https://clinicaltrials.gov/ct2/show/NCT01206062?term=NCT01206062&draw=2&rank=1.

| ***Yamada, et al. Mail-Based Intervention for Sarcopenia Prevention Increased Anabolic Hormone and Skeletal Muscle Mass in Community-Dwelling Japanese Older Adults: The INE (Intervention by Nutrition and Exercise) Study. J Am Med Dir Assoc. 2015;16(8):654-660. https://pubmed.ncbi.nlm.nih.gov/25858281/*** | | | | |
| --- | --- | --- | --- | --- |
| **Study design** | **Study type** | **Country/ Region** | **Clinical setting** | **Duration** |
|  | Randomized controlled trial | Japan | Communities | NR |
|  | **Intervention vs Control** | | **Sample size** | **Female (%)** |
|  | Walking and nutrition vs walking vs control | | 222 | 64.0 |
|  | **Age, years** | **Conditions** | **Follow-up** | **Loss to follow-up (%)** |
|  | Mean: 76 (range: 65+) | Fit, prefrail and frail adults. | 6 months | 2.2% |
| **Frailty assessment** | **Measurement tool** | **Frailty assessment** | | **Frailty at baseline, Intervention vs Control** |
|  | Frailty phenotype | Cardiovascular Health Study criteria (range: 0-5 criteria): Nonfrail [0-2 criteria] (N=151) Frail [≥ 3 criteria] (N=71) | | Nonfrail: 59.7% vs 79.6% vs 66.7% Frail: 40.3% vs 21.4% vs 33.3% |
| **Main findings** | **Outcome** | **Absolute effect** | **Analytic approach** | ***P*-value for interaction** |
|  | 6-month SMI | Nonfrail: 1.02% vs 1.11% vs -0.86% Frail: 3.16% vs 0.64% vs -3.87% | Stratified analysis | not available |
|  | 6-month IGF-1 | Nonfrail: 21.4% vs 22.5% vs 8.6% Frail: 31.8% vs 14.5% vs 9.4% |  |  |
|  | 6-month DHEA-S | Nonfrail: 26.6% vs 18.1% vs 8.4% Frail: 15.9% vs 19.7% vs -0.8% |  |  |
|  | 6-month 25(OH)D | Nonfrail: 39.9% vs 32.0% vs 6.1% Frail: 45.2% vs 33.6% vs -5.6% |  |  |
| **Risk of bias** | **Random sequence generation** | **Allocation concealment** | **Blinding of participants and personnel** | **Blinding of outcome assessment** |
|  | Unclear | Unclear | Unclear | Unclear |
|  | **Incomplete outcome data** | **Selective reporting** | **Other bias** |  |
|  | Low | Low | Low |  |

Abbreviations: NR, not reported; SMI, skeletal muscle mass index; IGF-1, insulinlike growth factor; DHEA-S, Dehydroepiandrosterone sulfate; 25(OH)D, 25-hydroxy vitamin D.

Note: The relative effects were not reported.
